# Acid-Catalyzed, Metal- and Oxidant-Free C=C Bond Cleavage of Enaminones: One-Pot Synthesis of 3,4-Dihydroquinazolines

**DOI:** 10.3390/molecules30020350

**Published:** 2025-01-16

**Authors:** Ting Chen, Ting Huang, Moudan Ye, Jinhai Shen

**Affiliations:** 1 School of Environment and Public Health, Xiamen Huaxia University, Xiamen 361024, China; chent@hxxy.edu.cn (T.C.); 220273128@stu.hxxy.edu.cn (T.H.); 210473213@stu.hxxy.edu.cn (M.Y.); 2 Xiamen Key Laboratory of Food and Drug Safety, Xiamen Huaxia University, Xiamen 361024, China

**Keywords:** 3,4-dihydroquinazoline, enaminones, C–C bond cleavage, ynones, organic synthesis

## Abstract

In this study, we present the HOAc-catalyzed selective cleavage of the C=C double bond of enaminones, enabling the formation of a new C–N bond and a new C=N bond for the one-pot synthesis of 2-substituted 3,4-dihydroquinazolines directly from ynones and 2-(aminomethyl)anilines. This method operates in ethanol under transition-metal-free and oxidant-free conditions, offering a sustainable and efficient approach for the synthesis of 3,4-dihydroquinazolines with broad functional group tolerance.

## 1. Introduction

**Figure 1:**
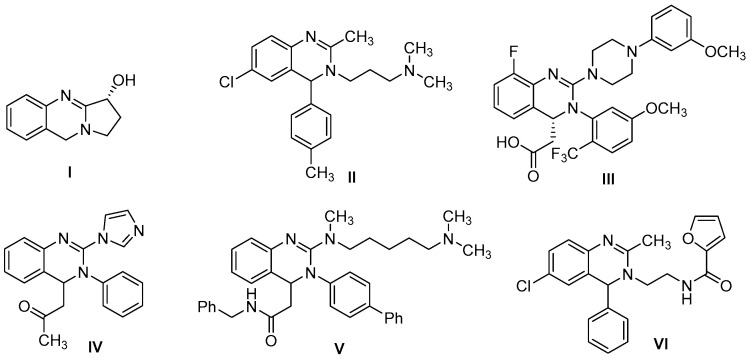
Biologically active 3,4-dihydroquinazolines.

3,4-Dihydroquinazoline is a versatile nitrogen-containing heterocyclic scaffold found in many bioactive compounds with diverse biological activities (Figure 1). For example, vasicine (**I**), a natural alkaloid from *Adhatoda vasica*, exhibits various bioactivities [1,2,3]. Synthetic derivatives of 3,4-dihydroquinazoline have been developed as antiparasitic (**II**) [4], antiviral (**III**) [5,6], and antifungal (**IV**) [7] agents, T-type calcium channel blockers (**V**) [8,9], and tryptophan reductase inhibitors (**VI**) [10]. Consequently, various synthetic methods have been established for the construction of 3,4-dihydroquinazoline skeletons [11,12,13,14,15,16,17,18,19,20,21,22]. However, most of reported reactions need harsh conditions and require metal catalysis or special reagents, such as harmful carbodiimide, isocyanate, azide, and isocyanide derivatives. The limitations of these methods for synthesizing such alkaloids heavily impede their biological studies. Hence, the discovery of novel and more direct approaches for the synthesis of such compounds from simple starting materials is highly welcome.

**Scheme 1:**
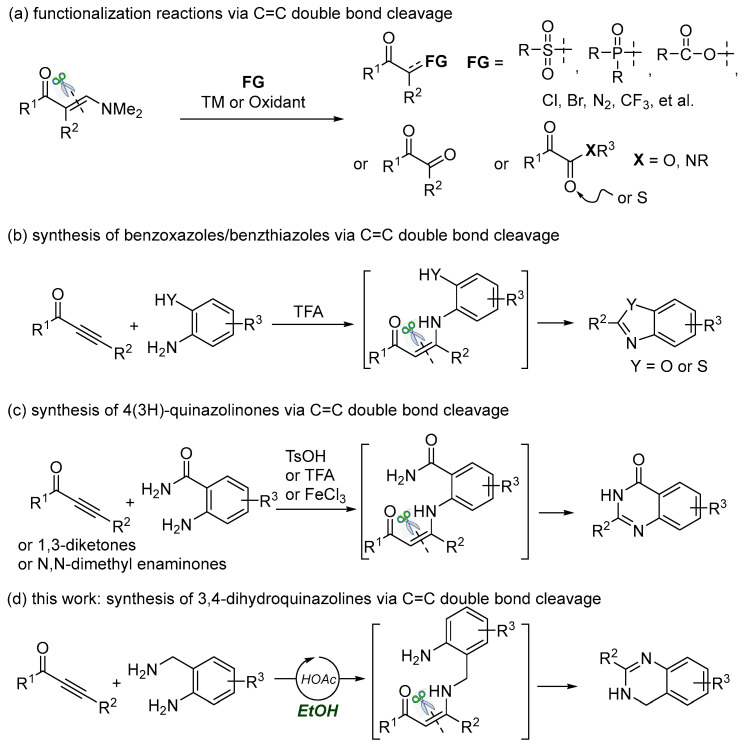
Functionalization reactions of enaminones via C=C double bond cleavage.

Enaminones, which bear an electron-donating amino group and electron-withdrawing carbonyl group(s) at both termini of a C=C double bond, respectively, are gaining increasing interest because of their unique properties and their importance in organic synthesis as versatile building blocks [23,24,25,26,27]. Particularly, the functionalization of the enaminone via vinyl C=C double bond cleavage is gaining great attention and developing rapidly [28,29,30,31,32,33,34,35,36,37,38,39,40,41,42,43]. On one hand, the construction of new chemical bonds has been identified as a highly beneficial strategy for the synthesis of many valuable chemical products, such as functionalized α-ketoamides [28], α-ketoesters [29], 1,2-diketones [30], and other non-cyclic compounds [31,32,33,34,35,36,37,38] (Scheme 1a). On the other hand, the construction of cyclic compounds via cyclization processes with the C=C double bond cleavage has also been achieved for the synthesis of benzoxazoles [39], benzthiazoles [40] (Scheme 1b), and 4(3H)-quinazolinones [41,42,43] (Scheme 1c). However, in order to avoid one or more of the limitations, such as harsh oxidative conditions, the use of noble metal catalysts, or a restricted substrate scope, a new strategy for oxidizing the olefin C=C double bond is still desired. As our successive efforts in enaminone chemistry [44,45], we report herein the acid-catalyzed selective cleavage of C=C double bond of β-enaminones, enabling the formation of a new C–N bond and a new C=N bond for the one-pot synthesis of 2-substituted 3,4-dihydroquinazolines directly from ynones and 2-(aminomethyl)anilines (Scheme 1d).

## 2. Results and Discussion

Initially, (*Z*)-3-((2-aminobenzyl)amino)-1,3-diphenylprop-2-en-1-one **1aa** was chosen as a model substrate to begin our exploration of the reaction conditions (Table 1). Under additive-free conditions, the reaction of β-enaminone **1aa** in ethanol at 100 °C for 4 h afforded the desired 2-phenyl-3,4-dihydroquinazoline **2aa** with a yield of 43%, with 48% of the starting material remaining unreacted (Entry 2). To improve the reaction efficiency, we systematically screened a variety of bases and acids as catalysts, including NaOH, KOH, Et_3_N, HOAc, TFA, TsOH·H_2_O, NH_4_Cl, and (NH_4_)_2_S_2_O_4_ (Entries 2–9). Among these, HOAc proved to be the most effective, delivering the target 3,4-dihydroquinazoline product with an excellent yield of 96%. The effect of the solvent on the reaction was also examined. Substituting ethanol with other solvents, such as methanol, isopropanol, DMSO, DMF, acetonitrile, toluene, or dioxane, led to a noticeable decline in efficiency (Entries 10–16, 37–91%). Additionally, temperature variation revealed that reducing the reaction temperature to 90 °C slightly lowered the yield to 92%, while increasing the temperature beyond 100 °C did not result in a further improvement (Entries 17–18). Finally, we evaluated the impact of substituent variation at the R^1^ position. Replacing the phenyl group with *p*-methoxyphenyl, *p*-fluorophenyl, or methyl groups resulted in yields of 89%, 94%, and 46%, respectively (Entries 19–21). The optimized reaction conditions were identified as follows: 20 mol% of HOAc as the additive and EtOH as the solvent at 100 °C for 4 h (Entry 5).

**Scheme 2:**
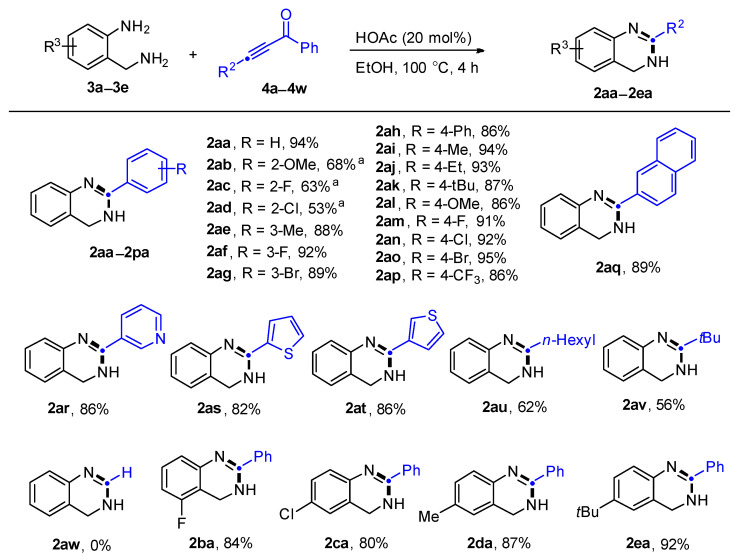
Substrate scope. ^a^ For 8 h.

As the enaminones **1** could be readily prepared from the Michael addition of the aliphatic amines **3** to ynones **4** [46], we employed a straightforward one-pot strategy to synthesize the desired 3,4-dihydroquinazoline products **2** directly from the 2-(aminomethyl)anilines **3** and ynones **4**, without isolating the enaminone intermediates **1**. To our delight, from the one-pot strategy, 2-phenyl-3,4-dihydroquinazoline **2aa** was afforded at a comparable yield to that from enaminone **1aa** (94% vs. 96%). Then, the scope of ynones **4** was explored. As illustrated in Scheme 2, When R^2^ was an aryl substituent, steric effects exert a pronounced influence on the reaction. Substrates with *ortho*-substituents, such as methoxy, fluorine, or chlorine, experience significant steric hindrance, which interferes with the nucleophilic attack of the amino group on the C=C double bond. Consequently, these substrates require longer reaction times, and the yields are generally lower compared to substrates with *para*- or *meta*-substituents (**2ab**–**2ad**, 53–68%). In contrast, substrates with *meta-* or *para-*substituents undergo the reaction smoothly, yielding the target 3,4-dihydroquinazoline products with good to excellent yields (**2ae**–**2ap**, 86–95%). The electronic effects of substituents on the phenyl ring play a lesser role in determining the reaction outcome. Both electron-donating groups (e.g., Me-, Et-, tBu-, and MeO-) and electron-withdrawing groups (e.g., F-, CF_3_-) at the *para*-position do not significantly alter the reactivity, allowing the reaction to proceed efficiently. As a result, high yields of the desired products are consistently achieved (**2ai**–**2ap**, 86–95%). Furthermore, the reaction demonstrates notable tolerance to a variety of halogen substituents, enabling the synthesis of halogenated 3,4-dihydroquinazoline derivatives (**2ac**–**2ad**, **2af**–**2ag**, **2am**–**2ao**). This tolerance is particularly advantageous for subsequent structural modifications, such as palladium-catalyzed coupling reactions, which could facilitate the further functionalization of the products.

In addition to phenyl rings, ynones substituted with other fused or heterocyclic aromatic groups (R^2^) also serve as excellent substrates for this reaction. Ynones substituted with a 2-naphthyl, 3-pyridyl, 2-thienyl, or 3-thienyl group undergo the reaction smoothly, yielding the target products at good to excellent yields (**2aq**–**2at**, 82–89%). Ynones bearing alkyl-substituted R^2^ groups, such as hexyl and tert-butyl, are also viable substrates in this reaction, albeit with relatively lower yields of 62% and 56%, respectively (**2au**–**2av**). This may be attributed to the absence of conjugation disruption in the corresponding products. When R^2^ was H (1-phenyl-prop-2-yn-1-one **4w** as substrate), the reaction failed to yield the targeted product **2aw**.

**Figure 2:**
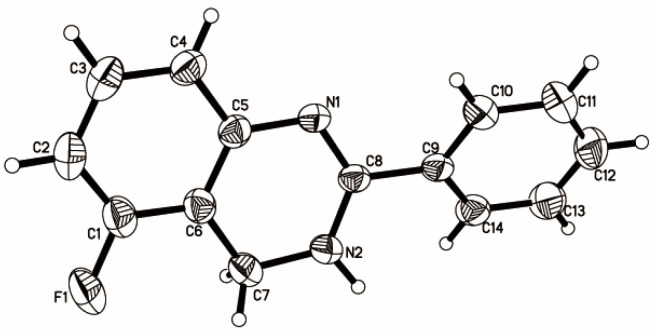
X-ray structure of **2ba**.

For 2-(aminomethyl)anilines, various functional groups are compatible, including electron-withdrawing groups, such as fluorine (**3b**) and chlorine (**3c**), as well as electron-donating groups, like methyl (**3d**) and *tert*-butyl (**3e**). The reaction with these substrates proceeded successfully, yielding the desired products at 84–92% yields (**2ba**–**2ea**). Additionally, we obtained single crystals of product **2ba**, and the structure of the target compound was further confirmed via single-crystal X-ray diffraction analysis (Figure 2). Based on the X-ray crystallographic data (see Supplementary Materials), the double bond is fixed rather than undergoing facile tautomerization.

**Scheme 3:**
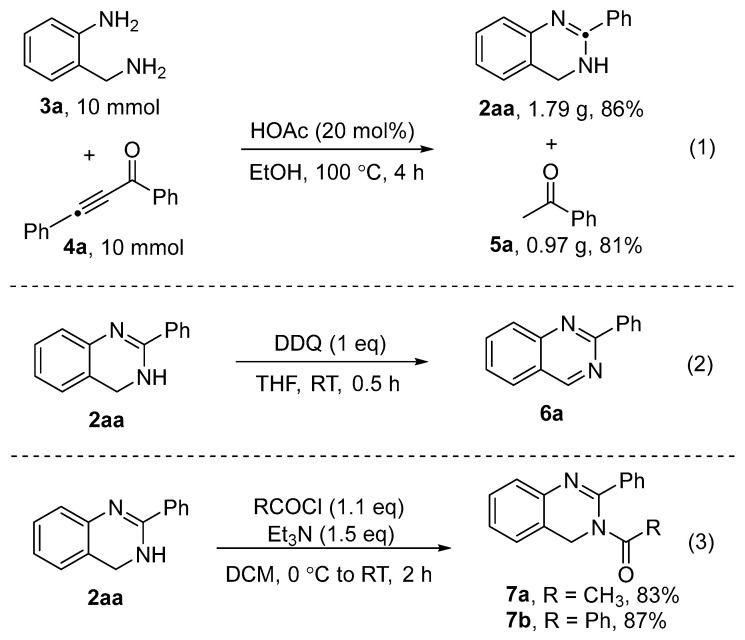
Gram-scale reaction and synthetic application.

To validate the practicality of this acid-catalyzed cascade transformation, a gram-scale one-pot synthesis of 2-phenyl-3,4-dihydroquinazoline **2aa** was carried out. Starting from 1.22 g of **3a** (10 mmol) and 2.06 g of **4a** (10 mmol), the reaction afforded 1.79 g of **2aa** with an overall yield of 86% (Scheme 3, Equation (1)). Notably, the reaction also produced acetophenone **5a** as a byproduct, which was isolated at an 81% yield. To further demonstrate the synthetic versatility of the obtained product, we explored its application in the preparation of quinazoline and acylated 3,4-dihydroquinazoline derivatives. The oxidation of **2aa** using DDQ as an oxidant at room temperature resulted in the efficient formation of 2-phenylquinazoline **6a** with a yield of 88% (Equation (2)). Additionally, **2aa** underwent smooth acylation using acetyl chloride and benzoyl chloride as acylating agents to furnish the corresponding acylated products, **7a** and **7b**, with yields of 83% and 87%, respectively (Equation (3)).

**Scheme 4:**
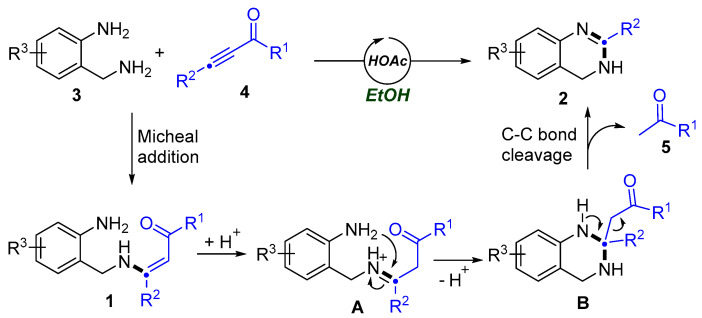
Proposed reaction mechanism.

Based on our study and the previous literature [39,40,41,42,43], a plausible reaction mechanism was proposed (Scheme 4). With the help of HOAc, the Michael addition of 2-(aminomethyl)anilines **3** to ynones **4** would generate the enaminone intermediates **1**. Iminoketone intermediates **A** may exist though the tautomerization of enaminones **1** in the presence of HOAc. Then, the intramolecular nucleophilic addition of **1** produced the intermediates **B**. After that, the C–C bond cleavage reaction occurred to generate the 3,4-dihydroquinazoline products **2** and byproduct **5**.

## 3. Experimental Sections

### 3.1. General Information

Unless otherwise stated, all commercial materials and solvents were used directly without further purification. ^1^H NMR spectra were recorded on 400 MHz spectrometers (Bruker, Karlsruhe, Germany) and ^13^C NMR spectra were recorded on a 100 MHz spectrometer. Chemical shifts (in ppm) were referenced to tetramethylsilane (δ = 0 ppm) in CDCl_3_ as an internal standard at room temperature. ^13^C NMR spectra were obtained by using the same NMR spectrometers and were calibrated with CDCl_3_ (δ = 77.00 ppm). High-resolution mass spectra (HRMS) were equipped with an ESI source and a TOF detector (Agilent, Santa Clara, CA, USA). Column chromatography was performed on a silica gel (70–230 mesh ASTM, Adamas-beta, Shanghai, China) using the reported eluents. Thin-layer chromatography (TLC) was carried out on 4 × 15 cm plates with a layer thickness of 0.2 mm (silica gel 60 F254). The ynone substrates were prepared according to the literature [47]. Enaminone **1aa** was prepared according to the literature [46].

### 3.2. General Procedure for the Synthesis of 3,4-Dihydroquinazolines ***2***

A mixture of ynones **4** (0.3 mmol), 2-(aminomethyl)anilines **3** (0.3 mmol, 1.0 equiv.), and HOAc (0.06 mmol, 20 mol%) in EtOH (1.5 mL) was stirred at 100 °C in a sealed tube for 4 h. After completion of the reaction (monitored by TLC), the reaction was quenched by 10 mL of water and made alkaline with 10% NaOH. The mixture was extracted with dichloromethane (3 × 10 mL). The organic layer was washed with water (20 mL), dried over sodium sulfate, and filtered. The solvent was removed in vacuo, and the crude products was purified via chromatography (silica gel, EtOAc/PE: 1/1 to MeOH/ EtOAc: 1/4) to give **2** (copies of ^1^H, ^13^C and ^19^F NMR spectra see Supplementary Materials). 

2-Phenyl-3,4-dihydroquinazoline **2aa**. 58.7 mg, 94% yield (one-pot operation). White solid, mp 134–136 °C. 1H NMR (400 MHz, CDCl_3_) δ 7.77 (d, *J* = 7.0 Hz, 2H), 7.40 (dq, *J* = 14.4, 7.1 Hz, 3H), 7.22–7.09 (m, 2H), 7.05 (td, *J* = 7.4, 1.0 Hz, 1H), 6.93 (d, *J* = 7.4 Hz, 1H), 4.89 (s, 1H), 4.74 (s, 2H); ^13^C NMR (100 MHz, CDCl_3_) δ 155.3, 139.9, 134.0, 131.0, 128.6, 128.2, 126.8, 125.6, 124.9, 121.1, 119.7, 44.4. HRMS (ESI) *m*/*z* calcd for C_14_H_13_N_2_ (MH+) 209.1073, found 209.1077. The NMR data are in accordance with those reported in the literature [48].

2-(2-Methoxyphenyl)-3,4-dihydroquinazoline **2ab**. 48.6 mg, 68% yield. White solid, mp 128–130 °C. ^1^H NMR (400 MHz, CDCl_3_) δ 7.99 (d, *J* = 7.7 Hz, 1H), 7.36 (t, *J* = 7.8 Hz, 1H), 7.15 (t, *J* = 7.6 Hz, 1H), 7.00 (dt, *J* = 15.3, 7.6 Hz, 3H), 6.95–6.84 (m, 2H), 6.26 (s, 1H), 4.71 (s, 2H), 3.86 (s, 3H); ^13^C NMR (100 MHz, CDCl_3_) δ 157.2, 154.9, 140.0, 131.9, 130.8, 127.9, 125.5, 124.3, 121.8, 121.1, 120.2, 119.8, 111.4, 55.8, 44.5. HRMS (ESI) *m*/*z* calcd for C_15_H_15_N_2_O (MH+) 239.1179, found 239.1180.

2-(2-Fluorophenyl)-3,4-dihydroquinazoline **2ac**. 42.7 mg, 63% yield. Pale yellow oil. ^1^H NMR (400 MHz, CDCl_3_) δ 8.01 (td, *J* = 7.9, 1.8 Hz, 1H), 7.42–7.35 (m, 1H), 7.22–7.14 (m, 2H), 7.12–6.98 (m, 3H), 6.93 (d, *J* = 7.2 Hz, 1H), 5.57 (s, 1H), 4.75 (s, 2H); ^13^C NMR (100 MHz, CDCl_3_) δ 160.5 (d, *J* = 248.0 Hz), 151.9, 140.7, 131.9 (d, *J* = 9.0 Hz), 130.9 (d, *J* = 2.6 Hz), 128.0, 125.5, 124.6 (d, *J* = 3.0 Hz), 124.6, 122.3 (d, *J* = 10.4 Hz), 121.1, 120.0, 116.0 (d, *J* = 23.4 Hz), 44.8; ^19^F NMR (376 MHz, CDCl_3_) δ −116.06 (s). The characterization data are in accordance with that reported in the literature [49].

2-(2-Chlorophenyl)-3,4-dihydroquinazoline **2ad**. 38.5 mg, 53% yield. Pale yellow solid, mp 126–128 °C. ^1^H NMR (400 MHz, CDCl_3_) δ 7.56 (dd, *J* = 7.3, 1.7 Hz, 1H), 7.41–7.26 (m, 3H), 7.16 (t, *J* = 7.5 Hz, 1H), 7.03 (t, *J* = 7.4 Hz, 1H), 6.95 (dd, *J* = 14.1, 7.6 Hz, 2H), 5.03 (s, 1H), 4.72 (s, 2H); ^13^C NMR (100 MHz, CDCl_3_) δ 154.5, 140.6, 135.1, 131.7, 130.8, 130.7, 129.9, 128.0, 127.1, 125.6, 124.6, 120.9, 119.9, 45.0; HRMS (ESI) *m*/*z* calcd for C_14_H_12_ClN_2_ (MH+) 243.0684, found 243.0684.

2-(*m*-Tolyl)-3,4-dihydroquinazoline **2ae**. 58.6 mg, 88% yield. White solid, mp 117–118 °C. ^1^H NMR (400 MHz, CDCl_3_) δ 7.64 (s, 1H), 7.53 (d, *J* = 7.3 Hz, 1H), 7.28 (dd, *J* = 11.7, 6.1 Hz, 2H), 7.18 (t, *J* = 7.4 Hz, 1H), 7.02 (t, *J* = 7.4 Hz, 2H), 6.94 (d, *J* = 7.3 Hz, 1H), 5.26 (dd, *J* = 304.8, 230.4 Hz, 1H), 4.77 (s, 2H), 2.39 (s, 3H); ^13^C NMR (101 MHz, CDCl_3_) δ 160.4, 155.0, 138.4, 135.4, 131.3, 128.6, 128.4, 128.0, 127.2, 125.5, 124.6, 123.3, 120.2, 44.4, 21.4. HRMS (ESI) *m*/*z* calcd for C_15_H_15_N_2_ (MH+) 223.1230, found 223.1235.

2-(3-Fluorophenyl)-3,4-dihydroquinazoline **2af**. 62.4 mg, 92% yield. White solid, mp 124–125 °C. ^1^H NMR (400 MHz, CDCl_3_) δ 7.54 (d, *J* = 8.1 Hz, 2H), 7.38 (dd, *J* = 13.9, 7.8 Hz, 1H), 7.16 (ddd, *J* = 11.7, 9.8, 4.5 Hz, 2H), 7.05 (dd, *J* = 12.6, 5.3 Hz, 2H), 6.94 (d, *J* = 7.3 Hz, 1H), 4.78 (s, 2H), 4.27 (s, 1H); ^13^C NMR (100 MHz, CDCl_3_) δ 162.8 (d, *J* = 246.9 Hz), 153.6, 140.9, 137.6 (d, *J* = 7.5 Hz), 130.2 (d, *J* = 8.1 Hz), 128.2, 125.5, 124.8, 122.1, 121.9 (d, *J* = 2.9 Hz), 120.1, 117.5 (d, *J* = 21.2 Hz), 113.8 (d, *J* = 23.2 Hz), 44.7; ^19^F NMR (376 MHz, CDCl_3_) δ −112.11 (s). HRMS (ESI) *m*/*z* calcd for C_14_H_12_FN_2_ (MH+) 227.0979, found 227.0978.

2-(3-Bromophenyl)-3,4-dihydroquinazoline **2ag**. 76.4 mg, 89% yield. White solid, mp 132–133 °C. ^1^H NMR (400 MHz, CDCl_3_) δ 7.93 (s, 1H), 7.67 (d, *J* = 7.7 Hz, 1H), 7.56 (d, *J* = 7.9 Hz, 1H), 7.26 (t, *J* = 7.8 Hz, 1H), 7.19 (t, *J* = 7.5 Hz, 1H), 7.05 (t, *J* = 8.3 Hz, 2H), 6.94 (d, *J* = 7.3 Hz, 1H), 4.94 (s, 1H), 4.77 (s, 2H); ^13^C NMR (100 MHz, CDCl_3_) δ 153.4, 140.9, 137.3, 133.5, 130.1, 129.7, 128.2, 125.6, 125.0, 124.9, 122.7, 121.8, 120.1, 44.7. HRMS (ESI) *m*/*z* calcd for C_14_H_12_BrN_2_ (MH+) 287.0178, found 287.0178.

2-([1,1′-Biphenyl]-4-yl)-3,4-dihydroquinazoline **2ah**. 73.3 mg, 86% yield. Grey solid, mp 193–195 °C. ^1^H NMR (400 MHz, CDCl_3_) δ 7.87 (d, *J* = 8.1 Hz, 2H), 7.61 (dd, *J* = 18.2, 7.7 Hz, 4H), 7.44 (t, *J* = 7.5 Hz, 2H), 7.37 (t, *J* = 7.2 Hz, 1H), 7.20 (t, *J* = 7.5 Hz, 1H), 7.11 (d, *J* = 7.6 Hz, 1H), 7.03 (t, *J* = 7.3 Hz, 1H), 6.94 (d, *J* = 7.0 Hz, 1H), 4.78 (s, 2H), 4.24 (s, 1H); ^13^C NMR (100 MHz, CDCl_3_) δ 154.5, 143.4, 140.1, 133.8, 128.9, 128.1, 127.8, 127.2, 127.1, 127.0, 127.0, 125.5, 124.6, 121.6, 120.2, 44.67. HRMS (ESI) *m*/*z* calcd for C_20_H_17_N_2_ (MH+) 285.1386, found 285.1389.

2-(*p*-Tolyl)-3,4-dihydroquinazoline **2ai**. 62.6 mg, 94% yield. White solid, mp 159–161 °C. ^1^H NMR (400 MHz, CDCl_3_) δ 7.72 (d, *J* = 8.0 Hz, 2H), 7.23–7.08 (m, 4H), 7.03 (t, *J* = 7.3 Hz, 1H), 6.90 (d, *J* = 7.4 Hz, 1H), 5.07 (s, 1H), 4.70 (s, 2H), 2.32 (s, 3H); ^13^C NMR (100 MHz, CDCl_3_) δ 155.1, 141.5, 139.8, 130.9, 129.3, 128.2, 126.7, 125.5, 124.7, 120.9, 119.8, 44.3, 21.4. HRMS (ESI) *m*/*z* calcd for C_15_H_15_N_2_ (MH+) 223.1230, found 223.1235.

2-(4-Ethylphenyl)-3,4-dihydroquinazoline **2aj**. 65.8 mg, 93% yield. White solid, mp 126–128 °C. ^1^H NMR (400 MHz, CDCl_3_) δ 7.70 (d, *J* = 7.8 Hz, 2H), 7.24 (t, *J* = 6.5 Hz, 2H), 7.18 (t, *J* = 7.5 Hz, 1H), 7.11–6.97 (m, 2H), 6.92 (d, *J* = 7.2 Hz, 1H), 4.82 (s, 1H), 4.74 (s, 2H), 2.67 (q, *J* = 7.5 Hz, 2H), 1.23 (t, *J* = 7.6 Hz, 3H); ^13^C NMR (100 MHz, CDCl_3_) δ 154.9, 147.1, 141.4, 132.7, 128.0, 128.0, 126.4, 125.5, 124.3, 121.6, 120.3, 44.7, 28.7, 15.4. HRMS (ESI) *m*/*z* calcd for C_26_H_17_N_2_ (MH+) 237.1386, found 237.1389.

2-(4-(*tert*-Butyl)phenyl)-3,4-dihydroquinazoline **2ak**. 76.8 mg, 87% yield. White solid, mp 148–150 °C. ^1^H NMR (400 MHz, CDCl_3_) δ 7.72 (d, *J* = 8.5 Hz, 2H), 7.43 (d, *J* = 8.5 Hz, 2H), 7.18 (t, *J* = 7.2 Hz, 1H), 7.12–6.98 (m, 2H), 6.94 (d, *J* = 7.3 Hz, 1H), 4.89 (d, *J* = 37.4 Hz, 1H), 4.76 (s, 2H), 1.33 (s, 9H); ^13^C NMR (10 MHz, CDCl_3_) δ 154.7, 153.9, 141.5, 132.6, 128.0, 126.1, 125.5, 125.5, 124.3, 120.3, 44.8, 34.8, 31.2. HRMS (ESI) *m*/*z* calcd for C_18_H_21_N_2_ (MH+) 265.1699, found 265.1703.

2-(4-Methoxyphenyl)-3,4-dihydroquinazoline **2al**. 61.4 mg, 86% yield. White solid, mp 151–153 °C. ^1^H NMR (400 MHz, CDCl_3_) δ 7.74 (d, *J* = 8.7 Hz, 2H), 7.18 (t, *J* = 7.5 Hz, 1H), 7.07 (d, *J* = 7.7 Hz, 1H), 7.02 (t, *J* = 7.3 Hz, 1H), 6.91 (t, *J* = 8.4 Hz, 3H), 4.72 (s, 2H), 4.48 (s, 1H), 3.82 (s, 3H); ^13^C NMR (100 MHz, CDCl_3_) δ 161.6, 154.6, 141.2, 128.1, 128.0, 127.3, 125.5, 124.3, 121.5, 120.2, 113.8, 55.4, 44.5. HRMS (ESI) *m*/*z* calcd for C_15_H_15_N_2_O (MH+) 239.1179, found 239.1179.

2-(4-Fluorophenyl)-3,4-dihydroquinazoline **2am**. 61.7 mg, 91% yield. White solid, mp 162–163 °C. ^1^H NMR (400 MHz, CDCl_3_) δ 7.85–7.77 (m, 2H), 7.22 (t, *J* = 7.5 Hz, 1H), 7.09 (dt, *J* = 13.5, 8.0 Hz, 4H), 6.97 (d, *J* = 7.3 Hz, 1H), 4.80 (s, 2H), 3.99 (s, 1H); ^13^C NMR (100 MHz, CDCl_3_) δ 164.2 (d, *J* = 250.6 Hz), 153.8, 141.2, 131.5 (d, *J* = 3.1 Hz), 128.6 (d, *J* = 8.6 Hz), 128.1, 125.5, 124.6, 121.8, 120.1, 115.6 (d, *J* = 21.8 Hz), 44.7; ^19^F NMR (376 MHz, CDCl_3_) δ −109.73 (s). HRMS (ESI) *m*/*z* calcd for C_14_H_12_FN_2_ (MH+) 227.0979, found 227.0979.

2-(4-Chlorophenyl)-3,4-dihydroquinazoline **2an**. 66.8 mg, 92% yield. White solid, mp 168–170 °C. ^1^H NMR (400 MHz, CDCl_3_) δ 7.77–7.69 (m, 2H), 7.43–7.34 (m, 2H), 7.19 (t, *J* = 7.6 Hz, 1H), 7.04 (dd, *J* = 11.5, 4.1 Hz, 2H), 6.94 (d, *J* = 7.4 Hz, 1H), 4.77 (s, 2H), 3.93 (s, 1H); ^13^C NMR (100 MHz, CDCl_3_) δ 153.7, 141.2, 136.6, 133.8, 128.8, 128.1, 127.8, 125.5, 124.7, 121.9, 120.1, 44.7. HRMS (ESI) *m*/*z* calcd for C_14_H_12_ClN_2_ (MH+) 243.0684, found 243.0683.

2-(4-Bromophenyl)-3,4-dihydroquinazoline **2ao**. 81.5 mg 95% yield. White solid, mp 173–175 °C. ^1^H NMR (400 MHz, CDCl_3_) δ 7.68 (d, *J* = 8.4 Hz, 2H), 7.56 (d, *J* = 8.5 Hz, 2H), 7.20 (t, *J* = 7.4 Hz, 1H), 7.05 (t, *J* = 7.3 Hz, 2H), 6.95 (d, *J* = 7.2 Hz, 1H), 5.27 (s, 1H), 4.79 (s, 2H); ^13^C NMR (100 MHz, CDCl_3_) δ 153.7, 141.2, 134.3, 131.7, 128.1, 128.0, 125.5, 124.9, 124.7, 120.1, 44.7. HRMS (ESI) *m*/*z* calcd for C_14_H_12_BrN_2_ (MH+) 287.0178, found 287.0178.

2-(4-(Trifluoromethyl)phenyl)-3,4-dihydroquinazoline **2ap**. 71.2 mg, 86% yield. White solid, mp 181–183 °C. ^1^H NMR (400 MHz, CDCl_3_) δ 7.89 (d, *J* = 8.1 Hz, 2H), 7.66 (d, *J* = 8.2 Hz, 2H), 7.21 (t, *J* = 7.5 Hz, 1H), 7.06 (t, *J* = 7.3 Hz, 2H), 6.96 (d, *J* = 7.4 Hz, 1H), 5.40 (s, 1H), 4.81 (s, 2H); ^13^C NMR (100 MHz, CDCl_3_) δ 153.4, 138.8, 132.3 (q, *J* = 32.6 Hz), 128.2, 126.8, 125.6, 125.6, 125.5, 125.2, 125.0, 122.4, 120.0, 44.7; ^19^F NMR (376 MHz, CDCl_3_) δ −62.78 (s). HRMS (ESI) *m*/*z* calcd for C_15_H_12_F_3_N_2_ (MH+) 277.0947, found 277.0946.

2-(Naphthalen-2-yl)-3,4-dihydroquinazoline **2aq**. 68.9 mg, 89% yield. Pale yellow solid, mp 156–158 °C. ^1^H NMR (400 MHz, CDCl_3_) δ 8.23 (s, 1H), 7.90 (dt, *J* = 17.8, 8.9 Hz, 4H), 7.52 (p, *J* = 7.2 Hz, 2H), 7.21 (t, *J* = 7.5 Hz, 1H), 7.13 (s, 1H), 7.05 (t, *J* = 7.4 Hz, 1H), 6.97 (d, *J* = 7.4 Hz, 1H), 4.83 (s, 2H), 4.82–4.77 (m, 1H); ^13^C NMR (100 MHz, CDCl_3_) δ 154.8, 134.3, 132.8, 132.7, 128.6, 128.4, 128.1, 127.7, 127.1, 126.6, 126.0, 125.5, 124.5, 123.8, 120.3, 44.7. HRMS (ESI) *m*/*z* calcd for C_18_H_15_N_2_ (MH+) 259.1230, found 259.1228.

2-(Pyridin-3-yl)-3,4-dihydroquinazoline **2ar**. 53.9 mg, 86% yield. Pale yellow solid, mp 126–128 °C. ^1^H NMR (400 MHz, CDCl_3_) δ 8.96 (d, *J* = 1.7 Hz, 1H), 8.57 (dd, *J* = 4.8, 1.3 Hz, 1H), 8.15 (dt, *J* = 8.0, 1.8 Hz, 1H), 7.27 (dd, *J* = 7.3, 5.4 Hz, 1H), 7.16 (d, *J* = 7.1 Hz, 1H), 7.06 (ddd, *J* = 10.1, 8.4, 4.1 Hz, 2H), 6.91 (d, *J* = 7.4 Hz, 1H), 6.18 (s, 1H), 4.74 (s, 2H); ^13^C NMR (100 MHz, CDCl_3_) δ 153.2, 151.4, 147.6, 139.5, 135.0, 130.0, 128.2, 125.6, 125.2, 123.4, 121.1, 119.5, 44.2. HRMS (ESI) *m*/*z* calcd for C_13_H_12_N_3_ (MH+) 210.1026, found 210.1028.

2-(Thiophen-2-yl)-3,4-dihydroquinazoline **2as**. 52.6 mg, 82% yield. White solid, mp 98–99 °C. ^1^H NMR (400 MHz, CDCl_3_) δ 7.44–7.37 (m, 2H), 7.18 (t, *J* = 7.5 Hz, 1H), 7.04 (dt, *J* = 14.7, 5.8 Hz, 3H), 6.94 (d, *J* = 7.4 Hz, 1H), 4.74 (s, 2H), 4.13 (s, 1H); ^13^C NMR (100 MHz, CDCl_3_) δ 149.8, 139.8, 128.8, 128.4, 128.1, 127.3, 126.4, 125.5, 125.3, 124.5, 120.5, 44.7. HRMS (ESI) *m*/*z* calcd for C_12_H_11_N_2_S (MH+) 215.0637, found 215.0638.

2-(Thiophen-3-yl)-3,4-dihydroquinazoline **2at**. 55.2 mg, 86% yield. White solid, mp 139–141 °C. ^1^H NMR (400 MHz, CDCl_3_) δ 7.77 (dd, *J* = 2.8, 1.1 Hz, 1H), 7.50 (dd, *J* = 5.1, 1.0 Hz, 1H), 7.33 (dd, *J* = 5.0, 3.0 Hz, 1H), 7.18 (t, *J* = 7.3 Hz, 1H), 7.03 (dd, *J* = 15.2, 7.7 Hz, 2H), 6.93 (d, *J* = 7.4 Hz, 1H), 4.74 (s, 2H), 4.25 (s, 1H); ^13^C NMR (100 MHz, CDCl_3_) δ 150.9, 141.0, 137.6, 128.1, 126.4, 126.1, 125.6, 125.2, 124.5, 121.5, 120.2, 44.5. HRMS (ESI) *m*/*z* calcd for C_12_H_11_N_2_S (MH+) 215.0637, found 215.0638.

2-Hexyl-3,4-dihydroquinazoline **2au**. 34.9 mg, 62% yield. Pale yellow oil. ^1^H NMR (400 MHz, CDCl_3_) δ 7.11 (d, *J* = 6.9 Hz, 1H), 7.00–6.92 (m, 1H), 6.88 (d, *J* = 6.3 Hz, 2H), 4.95 (s, 1H), 4.62 (d, *J* = 2.5 Hz, 2H), 2.32–2.13 (m, 2H), 1.63 (dt, *J* = 15.6, 7.9 Hz, 2H), 1.34 (d, *J* = 5.2 Hz, 2H), 1.28 (d, *J* = 3.1 Hz, 4H), 0.86 (d, *J* = 2.8 Hz, 3H); ^13^C NMR (100 MHz, CDCl_3_) δ 158.5, 140.7, 134.0, 127.8, 125.5, 123.7, 119.7, 44.8, 36.4, 31.5, 29.0, 27.2, 22.5, 14.0; HRMS (ESI) *m*/*z* calcd for C_14_H_21_N_2_ (MH+) 217.1699, found 217.1703. The characterization data are in accordance with those reported in the literature [50].

2-(*tert*-Butyl)-3,4-dihydroquinazoline **2av**. 36.3 mg, 56% yield. Pale yellow oil. ^1^H NMR (400 MHz, CDCl_3_) δ 7.42 (d, *J* = 7.4 Hz, 1H), 7.16 (t, *J* = 7.6 Hz, 1H), 7.08 (t, *J* = 7.4 Hz, 1H), 6.94 (d, *J* = 7.4 Hz, 1H), 4.75 (s, 2H), 4.21–3.99 (m, 1H), 1.42 (s, 9H); ^13^C NMR (100 MHz, CDCl_3_) δ 166.9, 134.7, 128.5, 125.8, 125.7, 118.2, 117.8, 44.0, 37.2, 27.7; HRMS (ESI) *m*/*z* calcd for C_12_H_17_N_2_ (MH+) 189.1386, found 189.1389.

5-Fluoro-2-phenyl-3,4-dihydroquinazoline **2ba**. 57.0 mg, 84% yield. White solid, mp 142–144 °C. ^1^H NMR (400 MHz, CDCl_3_) δ 7.75 (d, *J* = 7.4 Hz, 2H), 7.56–7.30 (m, 3H), 7.12 (dd, *J* = 14.7, 7.5 Hz, 1H), 6.86 (d, *J* = 7.7 Hz, 1H), 6.73 (t, *J* = 8.5 Hz, 1H), 4.79 (s, 3H); ^13^C NMR (100 MHz, CDCl_3_) δ 158.9 (d, *J* = 245.1 Hz), 155.3, 143.6, 135.2, 130.7, 128.6 (d, *J* = 9.3 Hz), 128.6, 126.4, 118.2 (d, *J* = 3.7 Hz), 110.8 (d, *J* = 21.0 Hz), 107.7 (d, *J* = 17.7 Hz), 38.9. HRMS (ESI) *m*/*z* calcd for C_14_H_12_FN_2_ (MH+) 227.0979, found 227.0979.

6-Chloro-2-phenyl-3,4-dihydroquinazoline **2ca**. 58.1 mg, 80% yield. White solid, mp 152–154 °C. ^1^H NMR (400 MHz, CDCl_3_) δ 7.80–7.73 (m, 2H), 7.49–7.37 (m, 3H), 7.14 (d, *J* = 8.4 Hz, 1H), 7.02 (d, *J* = 8.3 Hz, 1H), 6.91 (s, 1H), 4.72 (d, *J* = 2.6 Hz, 2H), 4.42 (s, 1H); ^13^C NMR (100 MHz, CDCl_3_) δ 155.1, 140.6, 135.1, 130.8, 129.2, 128.6, 128.1, 126.4, 125.4, 123.9, 121.9, 44.0. HRMS (ESI) *m*/*z* calcd for C_18_H_18_NO_3_ (MH+) 296.1281, found 296.1282.

6-Methyl-2-phenyl-3,4-dihydroquinazoline **2da**. 57.9 mg, 87% yield. Pale yellow oil. ^1^H NMR (400 MHz, CDCl_3_) δ 7.79 (d, *J* = 7.5 Hz, 2H), 7.37 (dt, *J* = 14.5, 7.1 Hz, 3H), 7.01 (dd, *J* = 28.8, 7.9 Hz, 2H), 6.72 (s, 1H), 5.60 (s, 1H), 4.68 (s, 2H), 2.29 (s, 3H); ^13^C NMR (100 MHz, CDCl_3_) δ 155.0, 137.0, 134.8, 133.6, 131.0, 128.8, 128.6, 126.8, 126.1, 121.2, 119.4, 44.1, 21.0. HRMS (ESI) *m*/*z* calcd for C_15_H_15_N_2_ (MH+) 223.1230, found 223.1233.

6-(*tert*-Butyl)-2-phenyl-3,4-dihydroquinazoline **2ea**. 72.9 mg, 92% yield. White solid, mp 152–154 °C. ^1^H NMR (400 MHz, CDCl_3_) δ 7.78 (d, *J* = 7.1 Hz, 2H), 7.39 (dd, *J* = 15.6, 8.1 Hz, 3H), 7.22 (d, *J* = 8.0 Hz, 1H), 7.05 (d, *J* = 8.2 Hz, 1H), 6.95 (s, 1H), 4.92 (s, 1H), 4.75 (s, 2H), 1.30 (s, 9H); ^13^C NMR (100 MHz, CDCl_3_) δ 154.8, 147.9, 138.1, 134.8, 130.7, 128.6, 126.6, 125.0, 122.4, 121.2, 119.3, 44.7, 34.4, 31.4. HRMS (ESI) *m*/*z* calcd for C_18_H_21_N_2_ (MH+) 265.1699, found 265.1703.

### 3.3. Procedure for Synthesis of ***6a***

2-Phenyl-3,4-dihydroquinazoline **2aa** (0.3 mmol) was dissolved in anhydrous THF (1 mL) and a solution of DDQ (0.3 mmol, 1 equiv.) in anhydrous THF (1 mL) was added. The reaction was stirred at room temperature for 30 min. The reaction was then quenched by the addition of aqueous NaOH solution (1 M, 1 mL). CH_2_Cl_2_ (10 mL) and H_2_O (10 mL) were added to the reaction, and the organic phase was separated. The aqueous phase was extracted with CH_2_Cl_2_ (2 × 10 mL) and the combined organic phases were dried with MgSO_4_ and filtered, and all volatiles were removed in vacuo. The residue was purified via flash column chromatography on silica (EtOAc/PE: 1/20) yielding 2-phenylquinazoline **6a**.

2-Phenylquinazoline **6a**. 54.4 mg, 88% yield. White solid, mp 99–101 °C. ^1^H NMR (400 MHz, CDCl_3_) δ 9.46 (s, 1H), 8.81–8.50 (m, 2H), 8.09 (d, *J* = 8.4 Hz, 1H), 7.98–7.82 (m, 2H), 7.65–7.44 (m, 4H); ^13^C NMR (100 MHz, CDCl_3_) δ 161.0, 160.5, 150.7, 138.0, 134.1, 130.6, 128.6, 128.6, 128.5, 127.2, 127.1, 123.6. The characterization data are in accordance with those reported in the literature [19] (copies of ^1^H and ^13^C NMR spectra see Supplementary Materials).

### 3.4. Procedure for Synthesis of ***7***

2-Phenyl-3,4-dihydroquinazoline **2aa** (0.3 mmol) was dissolved in anhydrous DCM (2 mL). The solution was cooled to 0 °C, and acyl chloride (0.33 mmol, 1.10 equiv.) and Et_3_N (0.45 mmol, 1.50 equiv.) were added. The mixture was stirred at RT for 2 h. The reaction was then quenched by the addition of H_2_O (10 mL). CH_2_Cl_2_ (10 mL) was added to the reaction, and the organic phase was separated. The aqueous phase was extracted with CH_2_Cl_2_ (2 × 10 mL), and the combined organic phases were dried with MgSO_4_ and filtered, and all volatiles were removed in vacuo. The residue was purified via flash column chromatography on silica (EtOAc/PE: 1/10), yielding **7a** and **7b** at 83% and 87%, respectively (copies of ^1^H and ^13^C NMR spectra see Supplementary Materials).

1-(2-Phenylquinazolin-3(4*H*)-yl)ethan-1-one **7a**. 62.3 mg, 83% yield. White solid, mp 86–88 °C. ^1^H NMR (400 MHz, CDCl_3_) δ 7.85 (dd, *J* = 7.8, 1.7 Hz, 2H), 7.55–7.44 (m, 4H), 7.35 (s, 1H), 7.22 (d, *J* = 8.1 Hz, 2H), 4.97 (s, 2H), 1.74 (s, 3H); ^13^C NMR (100 MHz, CDCl_3_) δ 170.5, 154.0, 142.1, 136.5, 131.3, 129.0, 128.5, 128.5, 127.1, 126.5, 125.3, 125.1, 42.9, 25.2; HRMS (ESI) *m*/*z* calcd for C_16_H_15_N_2_O (MH+) 251.1179, found 251.1177.

Phenyl(2-phenylquinazolin-3(4*H*)-yl)methanone **7b**. 81.4 mg, 87% yield. White solid, mp 130–132 °C. ^1^H NMR (400 MHz, CDCl_3_) δ 7.59–7.54 (m, 3H), 7.46–7.40 (m, 1H), 7.29 (dd, *J* = 11.8, 7.2 Hz, 4H), 7.20 (tt, *J* = 13.3, 6.6 Hz, 4H), 7.12 (t, *J* = 7.6 Hz, 2H), 5.10 (s, 2H); ^13^C NMR (100 MHz, CDCl_3_) δ 171.0, 155.0, 142.2, 136.5, 135.6, 131.3, 130.4, 128.6, 128.6, 128.2, 128.0, 127.1, 125.7, 125.3, 125.2, 45.0; HRMS (ESI) *m*/*z* calcd for C_21_H_17_N_2_O (MH+) 313.1335, found 313.1336.

## 4. Conclusions

In conclusion, we have successfully developed a transition-metal-free method for the synthesis of substituted 3,4-dihydroquinazolines via selective cleavage of the C=C bond of enaminones. This protocol utilizes only 0.2 equiv. of HOAc as an additive and employs ethanol as a green and sustainable solvent, making the process both efficient and environmentally friendly. Given the accessibility of the starting materials, the broad substrate scope, and the high reaction efficiencies, this acid-catalyzed cascade reaction holds significant potential for wide-ranging applications in synthetic organic chemistry.

## Figures and Tables

**Table 1:** Optimization of reaction conditions ^a^.

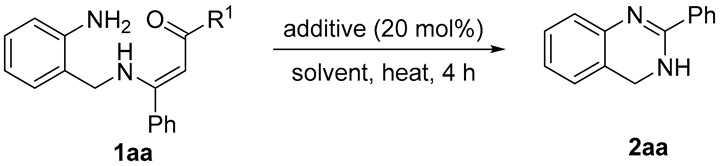					
Entry	Additive	Solvent	T (°C)	R^1^	Yield(%) ^b^
**1**	no	EtOH	100	Ph	43
**2**	NaOH	EtOH	100	Ph	52
**3**	KOH	EtOH	100	Ph	46
**4**	Et_3_N	EtOH	100	Ph	53
**5**	HOAc	EtOH	100	Ph	96
**6**	TFA	EtOH	100	Ph	88
**7**	TsOH·H_2_O	EtOH	100	Ph	91
**8**	NH_4_Cl	EtOH	100	Ph	94
**9**	(NH_4_)_2_S_2_O_4_	EtOH	100	Ph	93
**10**	HOAc	MeOH	100	Ph	88
**11**	HOAc	*i*PrOH	100	Ph	91
**12**	HOAc	DMSO	100	Ph	38
**13**	HOAc	DMF	100	Ph	88
**14**	HOAc	CH_3_CN	100	Ph	55
**15**	HOAc	PhMe	100	Ph	37
**16**	HOAc	dioxane	100	Ph	70
**17**	HOAc	EtOH	90	Ph	92
**18**	HOAc	EtOH	110	Ph	96
**19**	HOAc	EtOH	100	4–OMeC_6_H_4_–	89
**20**	HOAc	EtOH	100	4–FC_6_H_4_–	94
**21**	HOAc	EtOH	100	Me	46

^a^ Reaction conditions: **1aa** (0.2 mmol), additive (0.04 mmol), in 1 mL of solvent at 100 °C for 4 h. ^b^ Isolated yields. TFA = trifluoroacetic acid. TsOH = *p*-toluenesulfonic acid. DMF = *N*,*N*–dimethylformamide. DMSO = dimethylsulfoxide.

## Data Availability

Data are contained within the article and Supplementary Materials.

## Supplementary Materials

The following supporting information can be downloaded at: https://www.mdpi.com/article/10.3390/molecules30020350/s1, The crystallographic data of **2ba** and copies of the NMR (^1^H, ^13^C, ^19^F) spectra of all products are available in the online Supplementary Materials.
