# Supplementary material for: Acid-Catalyzed, Metal- and Oxidant-Free C=C Bond Cleavage of Enaminones: One-Pot Synthesis of 3,4-Dihydroquinazolines
Source: Molecules. 2025 Jan 16;30(2):350. doi: 10.3390/molecules30020350 (PMC11767836; doi:10.3390/molecules30020350)

Acid-Catalyzed, Metal- and Oxidant-Free C=C Bond Cleavage of Enaminones: One-Pot Syntheses of 3,4-Dihydroquinazolines

Ting Chen [1,2], Ting Huang [1], Moudan Ye [1], Jinhai Shen [1,2,*]

[1] School of Environment and Public Health, Xiamen Huaxia University, Xiamen 361024, China.

[2] Xiamen Key Laboratory of Food and Drug Safety, Xiamen Huaxia University, Xiamen 361024, China.

* Correspondence: E-mail: shenjh@hxxy.edu.cn; Tel.: (+86)592-6276260.

Contents

1. Single crystal structure and crystallographic data for **2ba** --S2

2. ^{1}H NMR, $^{13}C\{^{1}H\}$ NMR and ^{19}F NMR spectra of products --S6

1. Single crystal structure and crystallographic data for 2ba

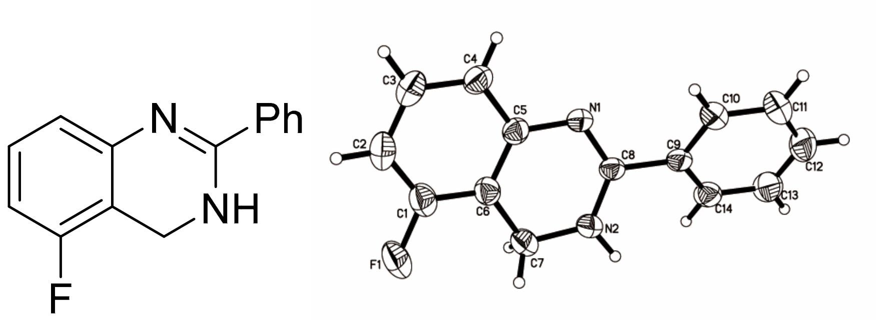

2ba (CCDC: 2403844, the ellipsoid contour probability level is 50%)

Table S1 Crystal data and structure refinement for 2ba.

Identification code	**2ba**
Empirical formula	$C_{14}H_{11}FN_2$
Formula weight	226.25
Temperature/K	293(2)
Crystal system	monoclinic
Space group	$P2_1/c$
a/Å	8.6627(4)
b/Å	14.2162(7)
c/Å	9.5015(3)
α/°	90
β/°	93.211(4)
γ/°	90
Volume/Å^3	1168.28(9)
Z	4
ρ_{calc}g/cm^3	1.286
μ/mm^{-1}	0.722
F(000)	472.0
Crystal size/mm^3	0.14 × 0.11 × 0.1
Radiation	CuKα (λ = 1.54184)
2Θ range for data collection/°	10.228 to 134.118
Index ranges	$-10 \le h \le 10$, $-16 \le k \le 16$, $-6 \le l \le 11$

Reflections collected	4162
Independent reflections	2084 [R_{int} = 0.0257, R_{sigma} = 0.0342]
Data/restraints/parameters	2084/0/159
Goodness-of-fit on F^2	1.036
Final R indexes [I>=2σ (I)]	R_1 = 0.0483, wR_2 = 0.1414
Final R indexes [all data]	R_1 = 0.0620, wR_2 = 0.1584
Largest diff. peak/hole / e Å^{-3}	0.13/-0.16

Table S2 Fractional Atomic Coordinates (×10^4) and Equivalent Isotropic Displacement Parameters (Å^2×10^3) for 2ba. U_{eq} is defined as 1/3 of of the trace of the orthogonalised U_{IJ} tensor.

Atom	*x*	*y*	*z*	U(eq)
F1	8240.5(18)	4490.7(14)	3627.6(17)	106.1(6)
N1	4094.8(18)	3253.7(11)	6194.6(13)	55.8(4)
N2	4349.1(19)	2757.1(12)	3864.6(15)	58.7(4)
C1	7461(2)	4461.4(17)	4831(2)	71.0(6)
C2	7917(2)	5047.2(17)	5918(3)	76.6(6)
C3	7104(3)	5012.0(16)	7124(2)	73.7(6)
C4	5860(2)	4409.0(15)	7209.2(19)	65.6(5)
C5	5410(2)	3821.0(13)	6090.1(17)	54.5(4)
C6	6251(2)	3838.1(14)	4878.7(18)	58.1(5)
C7	5840(2)	3185.6(17)	3679(2)	69.0(5)
C8	3618(2)	2792.1(12)	5066.7(15)	49.1(4)
C9	2115(2)	2290.4(13)	5091.4(15)	53.9(4)
C10	976(3)	2652.8(17)	5903(2)	70.8(6)
C11	-455(3)	2218(2)	5921(3)	88.7(8)
C12	-760(3)	1435(2)	5107(3)	93.2(8)
C13	358(3)	1072.0(18)	4286(2)	83.6(7)
C14	1796(3)	1492.7(15)	4282.2(18)	65.3(5)

Table S3 Anisotropic Displacement Parameters (Å^2×10^3) for 2ba. The Anisotropic displacement factor exponent takes the form: $-2\pi^2[h^2a^{*2}U_{11}+2hka^*b^*U_{12}+\ldots]$.

Atom	U_{11}	U_{22}	U_{33}	U_{23}	U_{13}	U_{12}
F1	75.7(9)	134.8(14)	111.6(11)	-4.6(10)	37.7(8)	-32.3(9)
N1	60.1(8)	67.6(9)	39.9(6)	1.3(6)	3.1(5)	-10.2(7)
N2	61.6(9)	68.3(10)	47.1(7)	-6.2(7)	10.1(6)	-9.3(7)
C1	51.7(10)	79.5(14)	82.2(12)	8.4(10)	7.3(9)	-2.4(9)
C2	55.5(10)	72.2(13)	100.7(15)	7.8(11)	-7.4(10)	-13.8(9)
C3	70.4(12)	68.1(13)	79.7(13)	-1.7(10)	-21.4(10)	-6.0(10)
C4	68.4(12)	72.7(12)	54.4(9)	0.2(8)	-7.5(8)	-6.5(9)
C5	53.4(9)	58.3(10)	50.9(8)	5.2(7)	-4.4(7)	-1.2(8)
C6	48.6(8)	65.0(11)	60.5(9)	3.8(8)	2.7(7)	0.5(8)
C7	61.3(10)	84.3(13)	63.2(10)	-6.9(10)	18.9(8)	-7.3(10)
C8	55.1(9)	52.7(9)	39.5(7)	5.2(6)	1.7(6)	-0.9(7)
C9	60.3(10)	61.6(10)	39.7(7)	7.7(7)	2.1(6)	-8.7(8)
C10	66.4(12)	85.4(14)	61.3(10)	-4.1(9)	12.0(8)	-13.4(11)
C11	65.0(12)	112(2)	90.7(15)	1.7(14)	20.4(11)	-14.9(13)
C12	75.3(15)	110(2)	94.1(15)	10.7(15)	1.8(12)	-41.5(15)
C13	95.5(16)	78.1(14)	76.9(12)	3.5(11)	1.9(11)	-35.1(13)
C14	77.7(12)	64.6(11)	53.7(9)	3.4(8)	4.5(8)	-15.3(10)

Table S4 Bond Lengths for 2ba.

Atom	Atom	Length/Å	Atom	Atom	Length/Å
F1	C1	1.361(3)	C5	C6	1.396(3)
N1	C5	1.404(2)	C6	C7	1.497(3)
N1	C8	1.304(2)	C8	C9	1.486(2)
N2	C7	1.448(3)	C9	C10	1.385(3)
N2	C8	1.337(2)	C9	C14	1.389(3)
C1	C2	1.367(3)	C10	C11	1.386(3)

C1	C6	1.376(3)	C11	C12	1.374(4)
C2	C3	1.380(3)	C12	C13	1.377(4)
C3	C4	1.383(3)	C13	C14	1.382(3)
C4	C5	1.391(3)			

Table S5 Bond Angles for 2ba.

**Atom**	**Atom**	**Atom**	**Angle/°**	**Atom**	**Atom**	**Atom**	**Angle/°**
C8	N1	C5	116.87(13)	C5	C6	C7	120.27(17)
C8	N2	C7	123.83(15)	N2	C7	C6	110.10(15)
F1	C1	C2	118.7(2)	N1	C8	N2	125.27(16)
F1	C1	C6	117.5(2)	N1	C8	C9	117.78(14)
C2	C1	C6	123.8(2)	N2	C8	C9	116.92(14)
C1	C2	C3	117.8(2)	C10	C9	C8	118.84(17)
C2	C3	C4	120.5(2)	C10	C9	C14	119.09(18)
C3	C4	C5	120.92(19)	C14	C9	C8	122.02(17)
C4	C5	N1	119.01(16)	C9	C10	C11	120.4(2)
C4	C5	C6	118.86(18)	C12	C11	C10	119.9(2)
C6	C5	N1	122.09(16)	C11	C12	C13	120.2(2)
C1	C6	C5	118.17(18)	C12	C13	C14	120.1(2)
C1	C6	C7	121.55(18)	C13	C14	C9	120.2(2)

2. ^{1}H NMR, ^{13}C NMR and ^{19}F NMR spectra of products:

^{1}H NMR (400 MHz, $CDCl_3$) spectrum of **2aa**

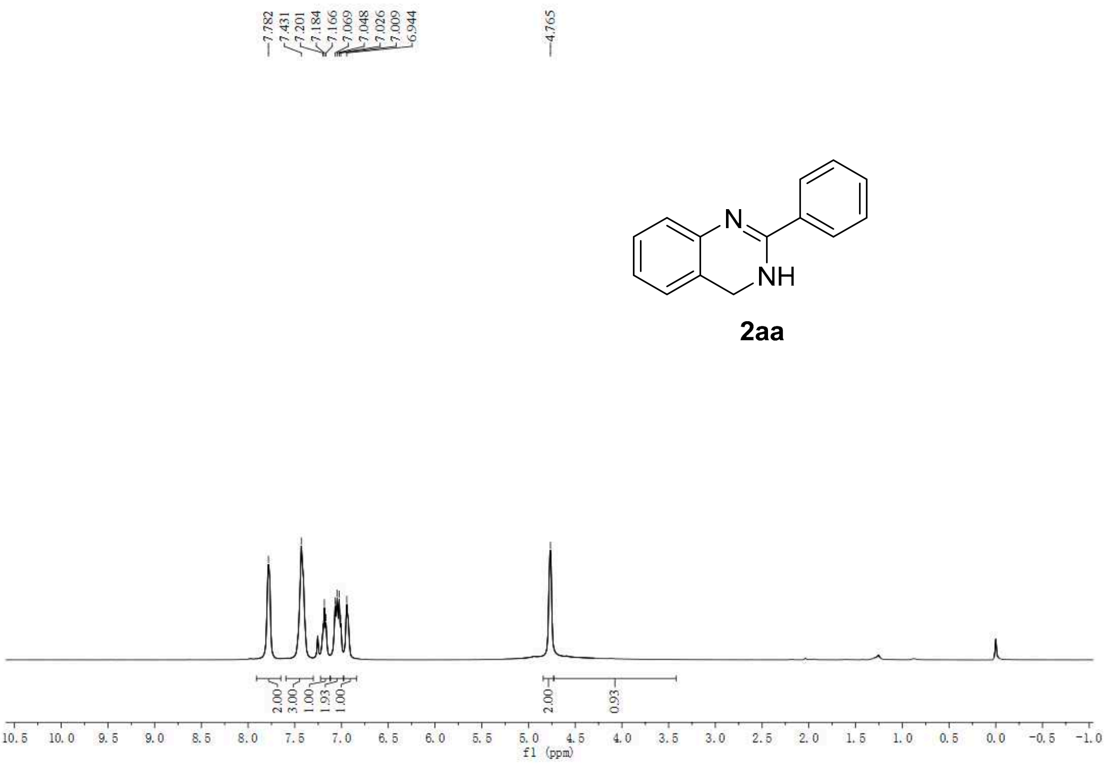

^{13}C {^{1}H} NMR (100 MHz, $CDCl_3$) spectrum of **2aa**

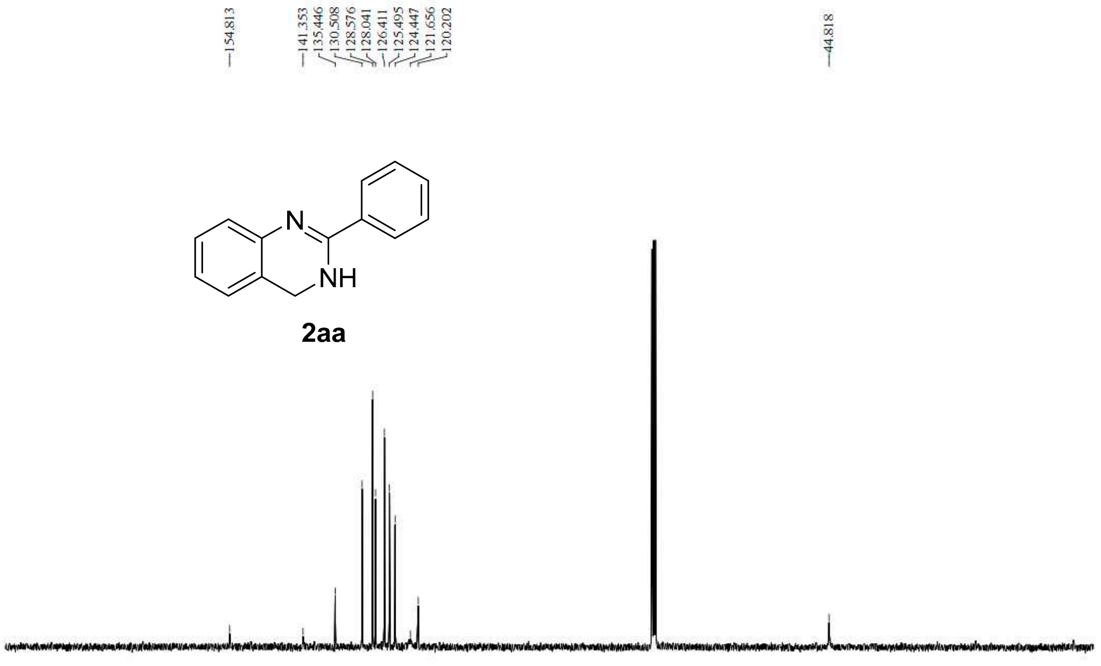

^{1}H NMR (400 MHz, $CDCl_3$) spectrum of **2ab**

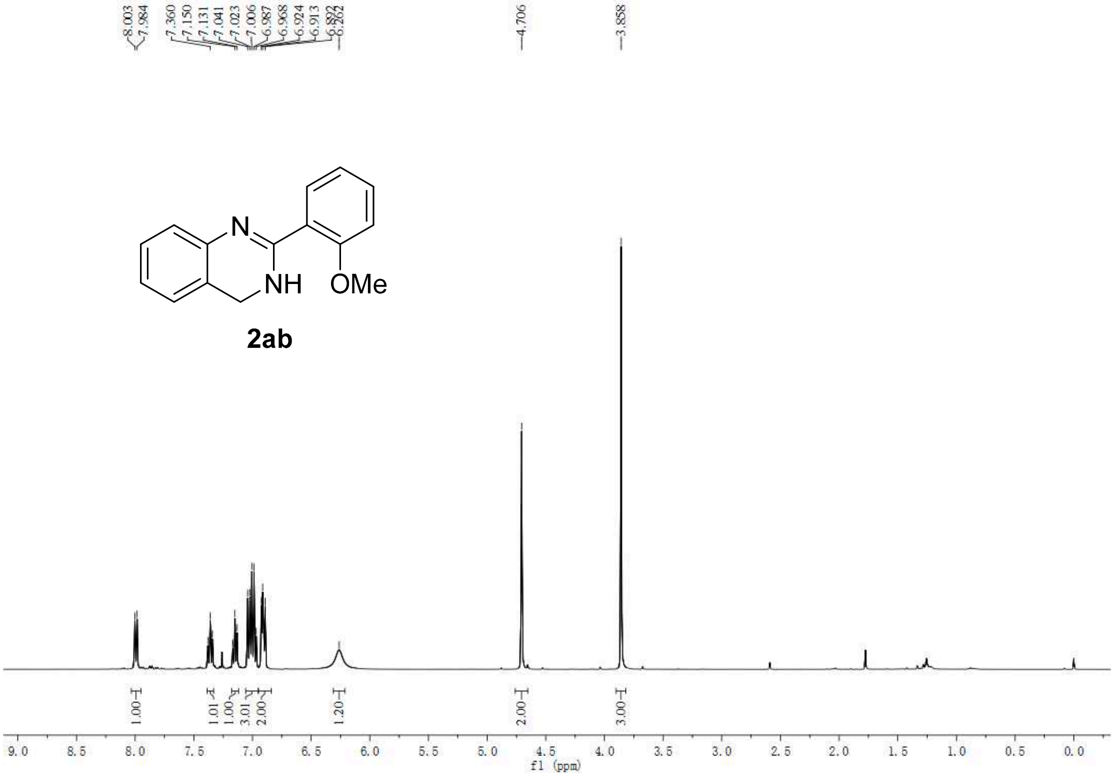

^{13}C {^{1}H} NMR (100 MHz, $CDCl_3$) spectrum of **2ab**

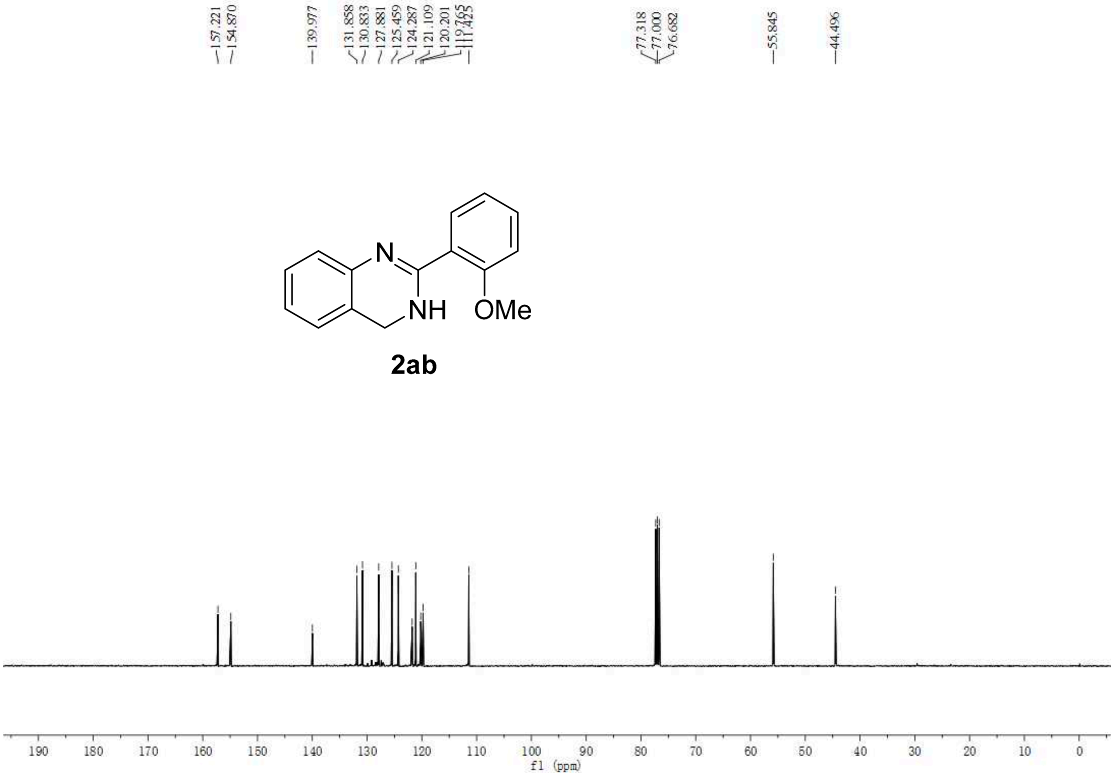

^{1}H NMR (400 MHz, $CDCl_3$) spectrum of **2ac**

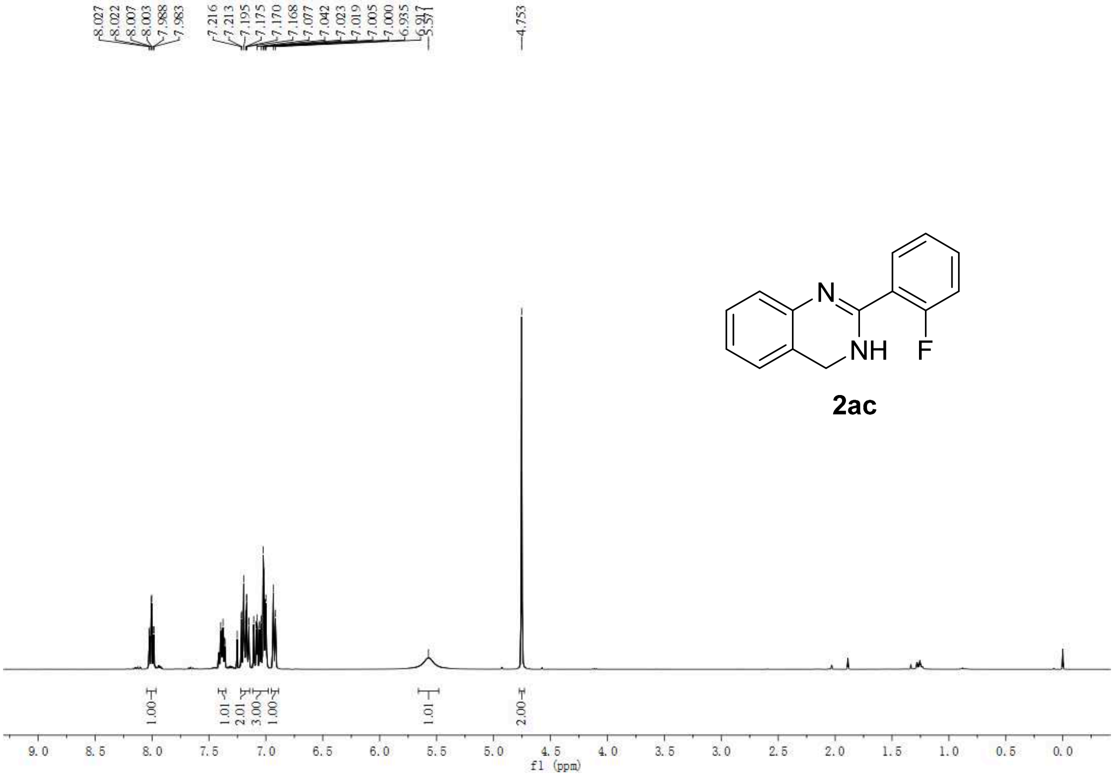

^{13}C {^{1}H} NMR (100 MHz, $CDCl_3$) spectrum of **2ac**

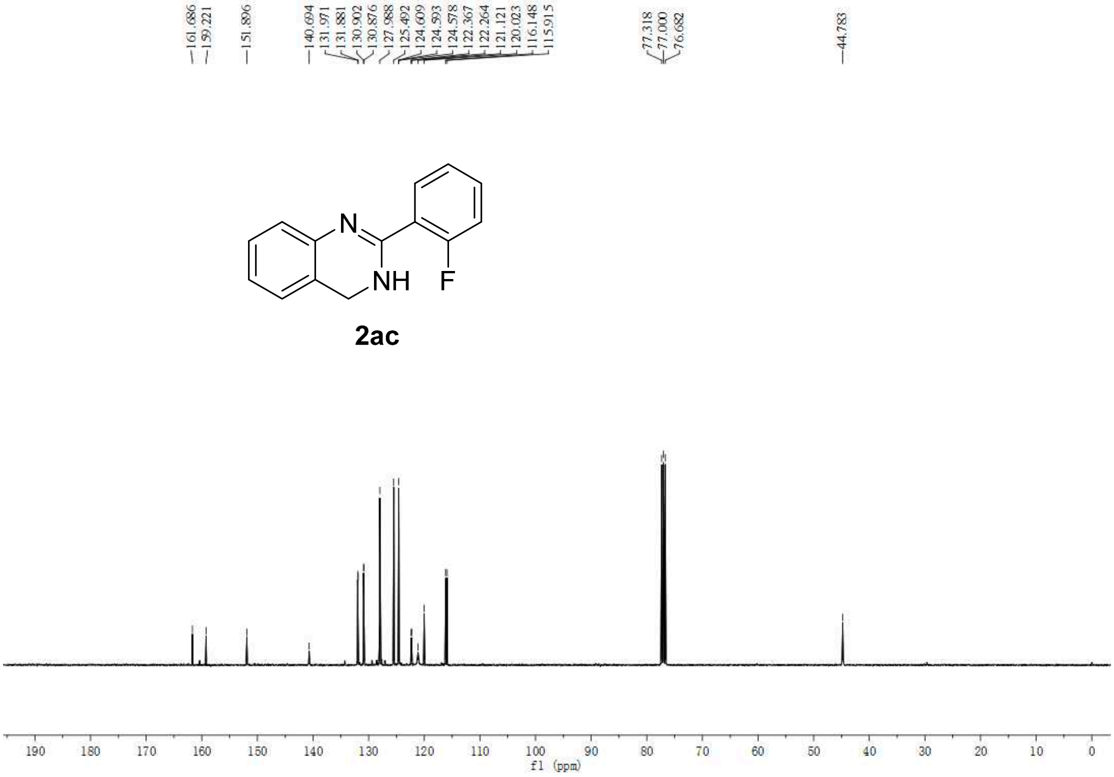

^{19}F (376 MHz, $CDCl_3$) spectrum of **2ac**

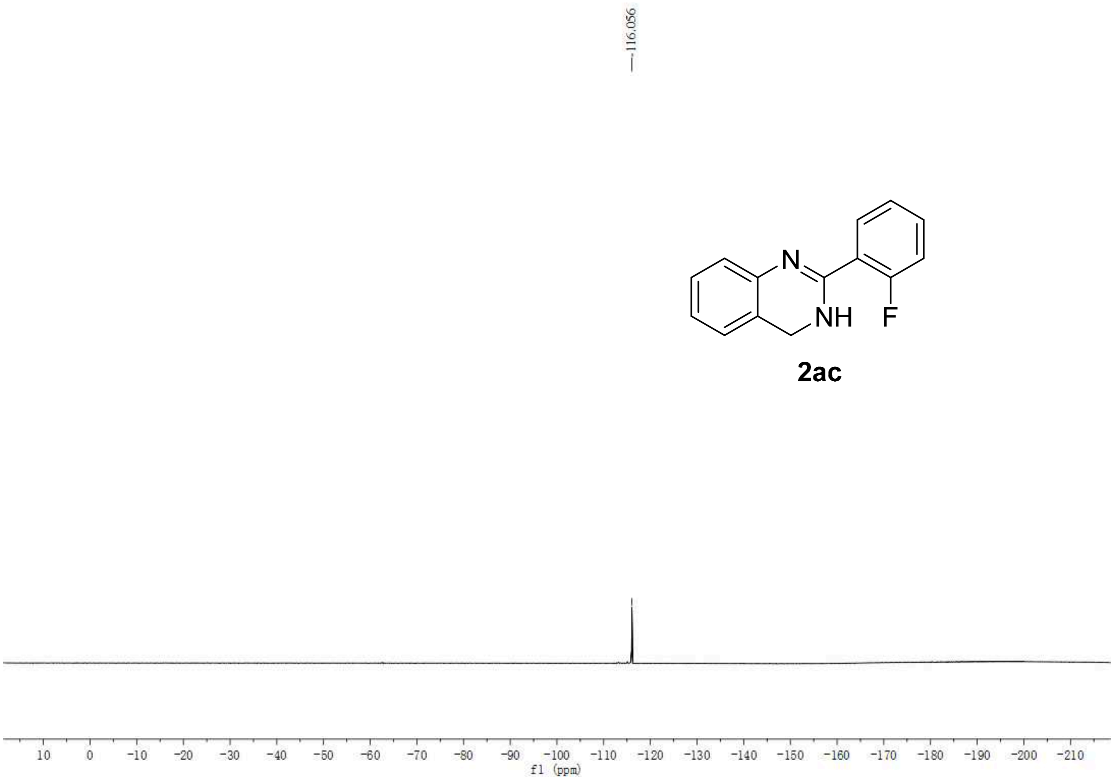

^{1}H NMR (400 MHz, $CDCl_3$) spectrum of **2ad**

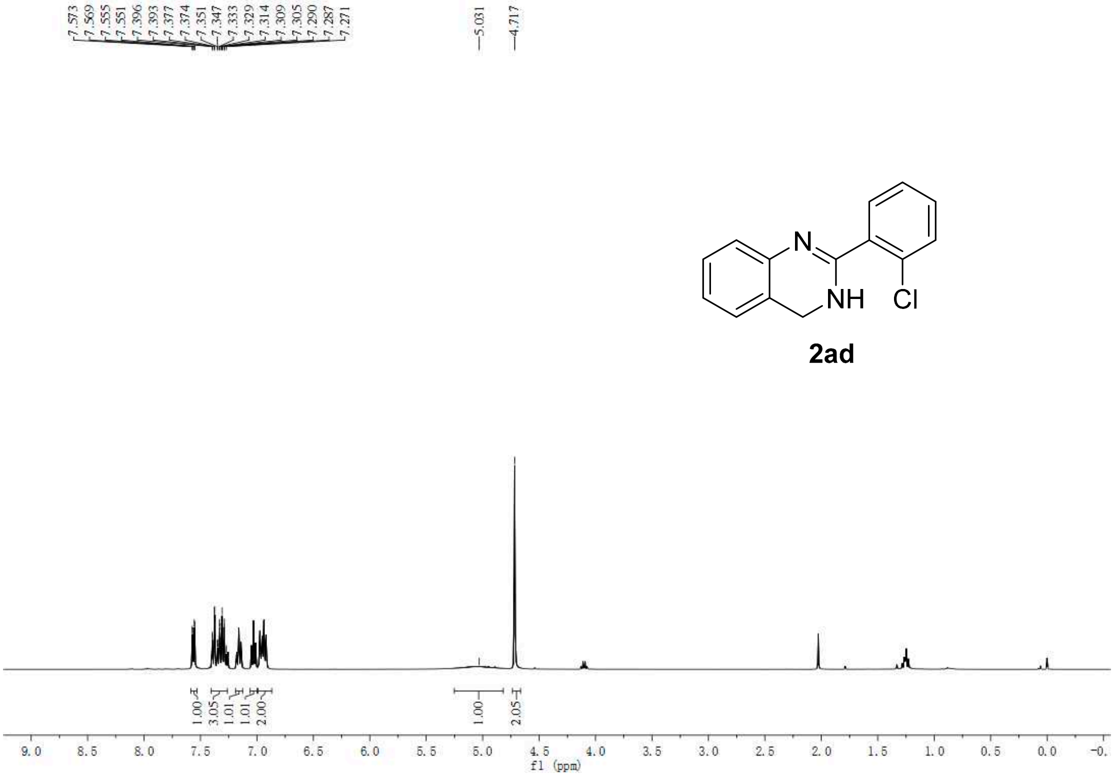

$^{13}C\ \{^{1}H\}$ NMR (100 MHz, $CDCl_3$) spectrum of **2ad**

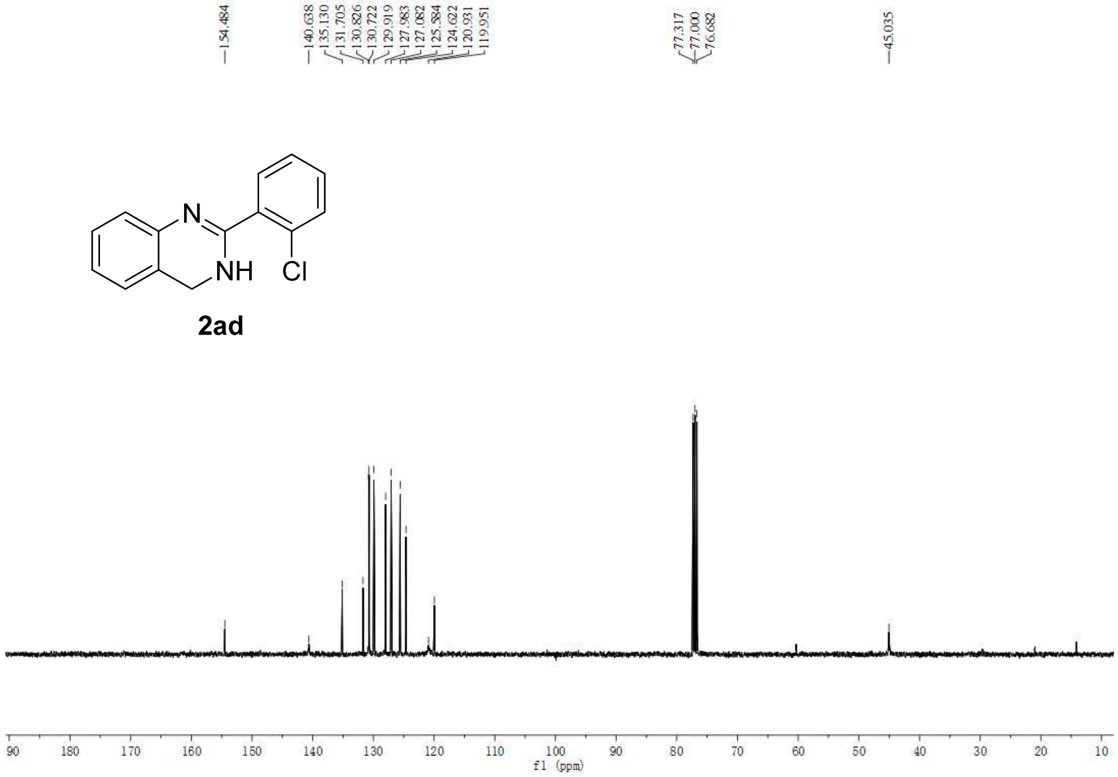

^{1}H NMR (400 MHz, $CDCl_3$) spectrum of **2ae**

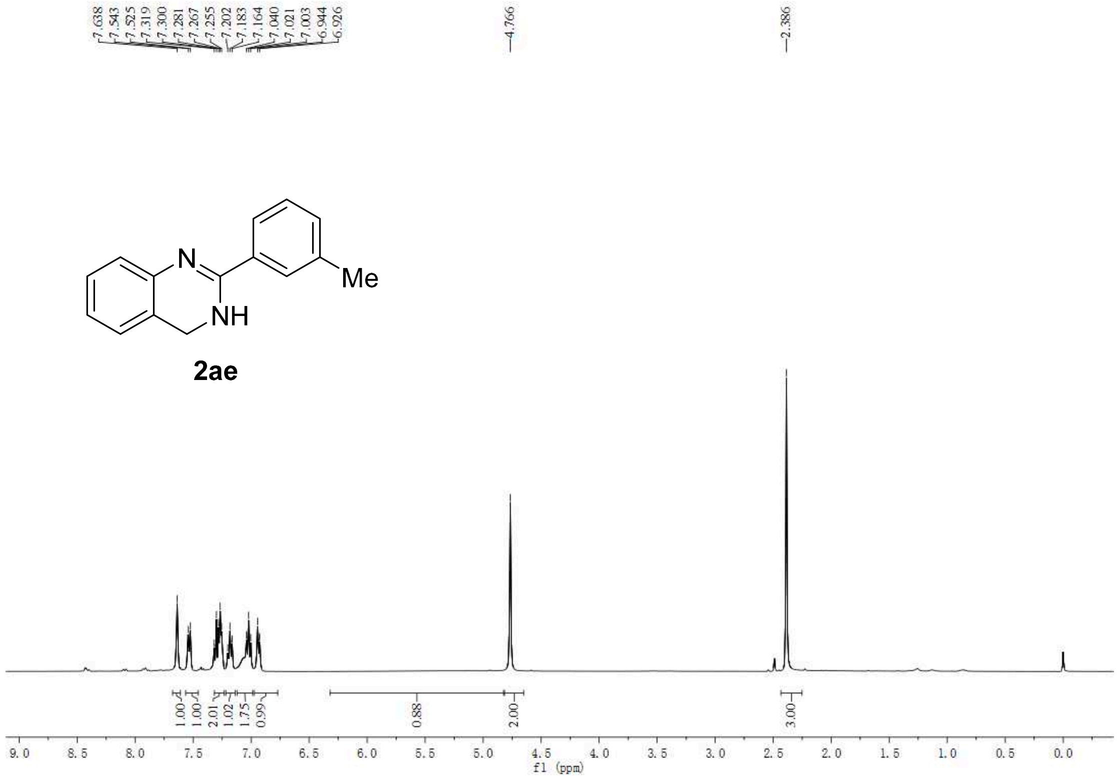

$^{13}C\ \{^{1}H\}$ NMR (100 MHz, $CDCl_3$) spectrum of **2ae**

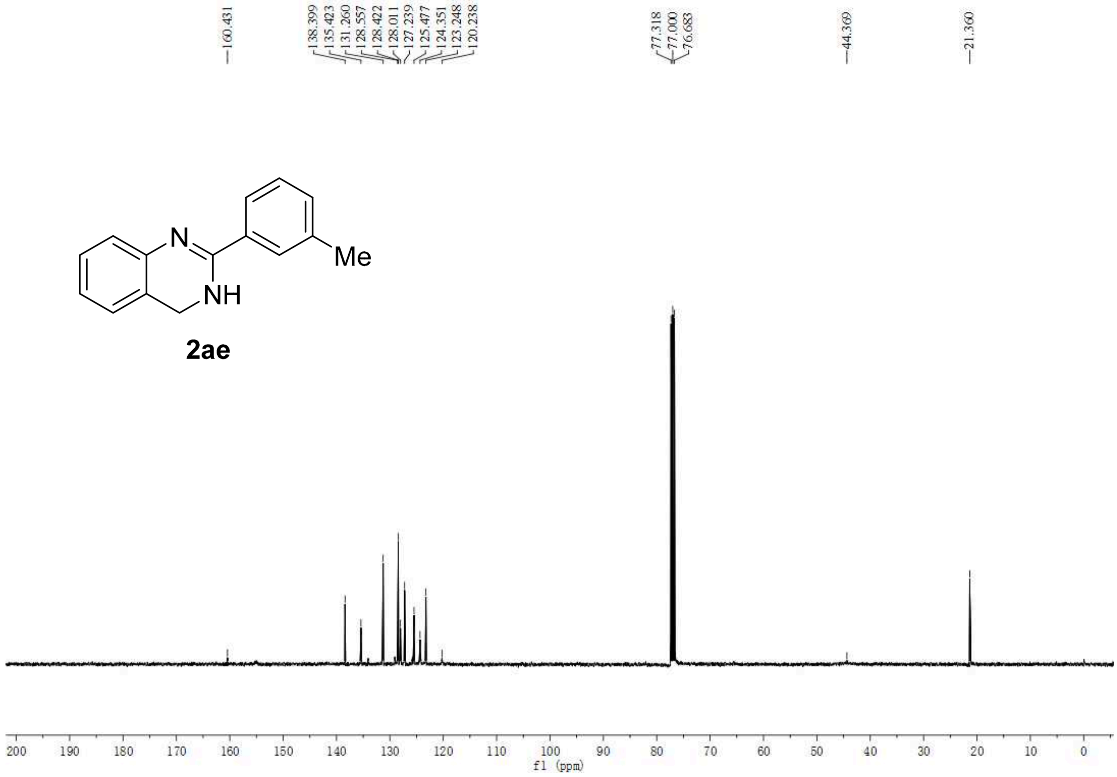

^{1}H NMR (400 MHz, $CDCl_3$) spectrum of **2af**

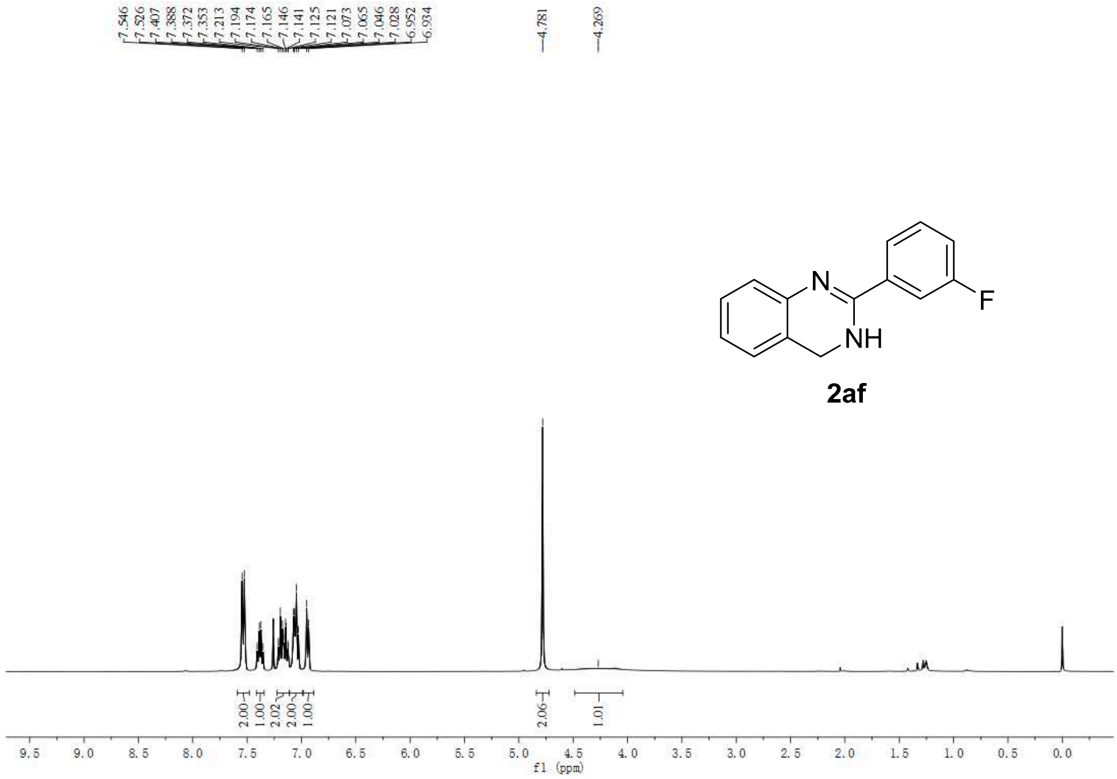

^{13}C {^{1}H} NMR (100 MHz, $CDCl_3$) spectrum of **2af**

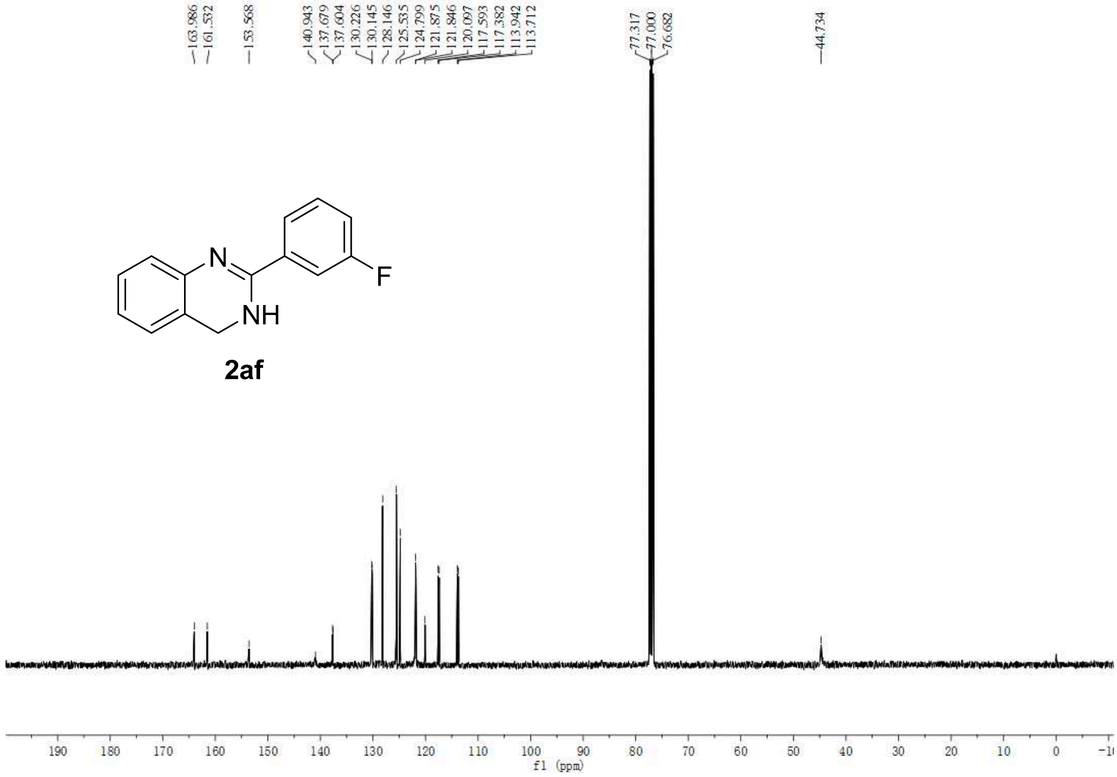

^{19}F (376 MHz, $CDCl_3$) spectrum of **2af**

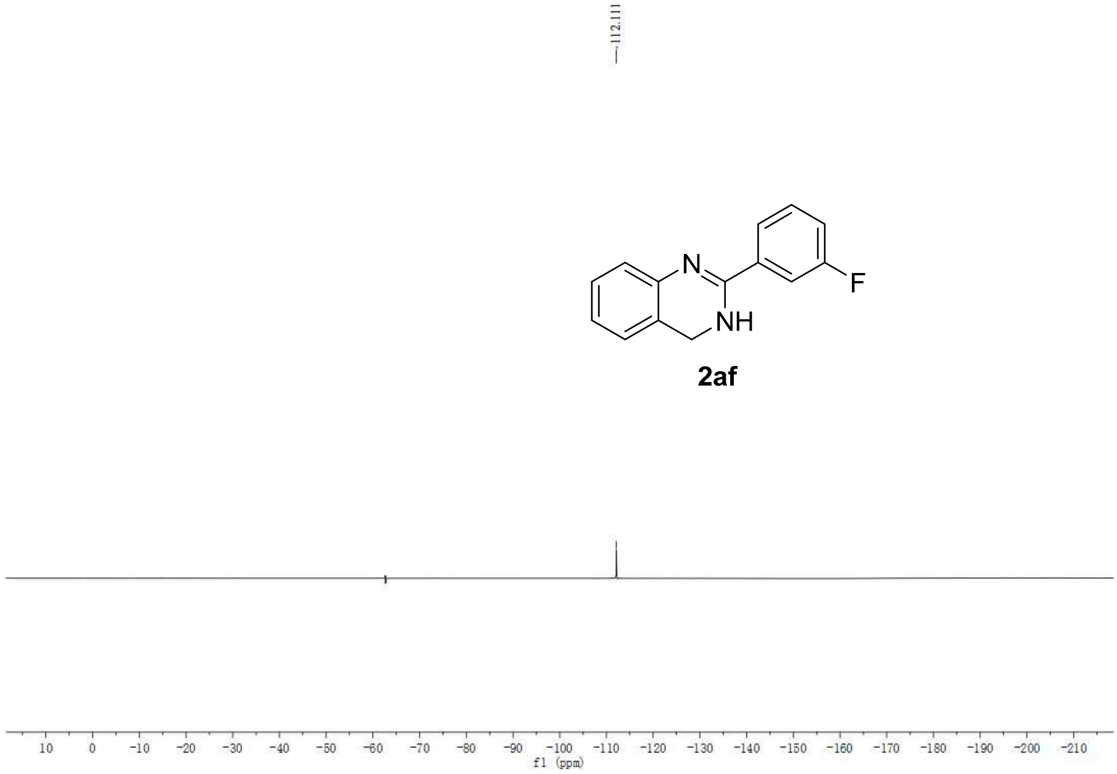

^{1}H NMR (400 MHz, $CDCl_3$) spectrum of **2ag**

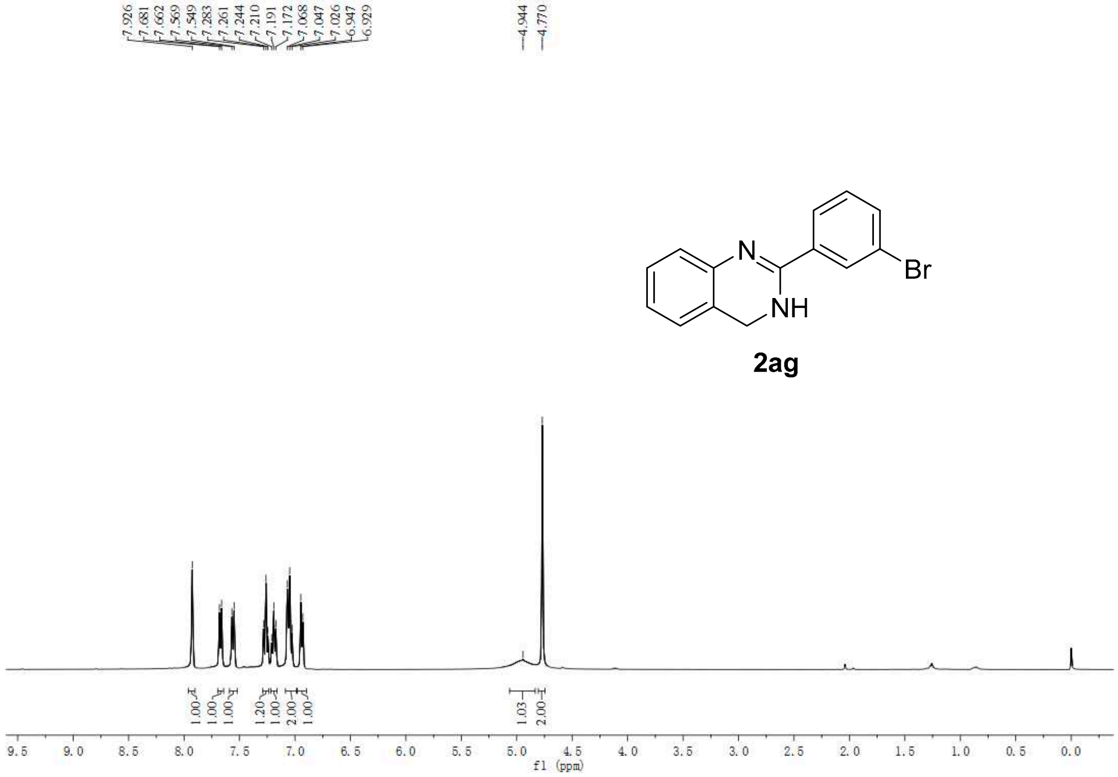

^{13}C {^{1}H} NMR (100 MHz, $CDCl_3$) spectrum of **2ag**

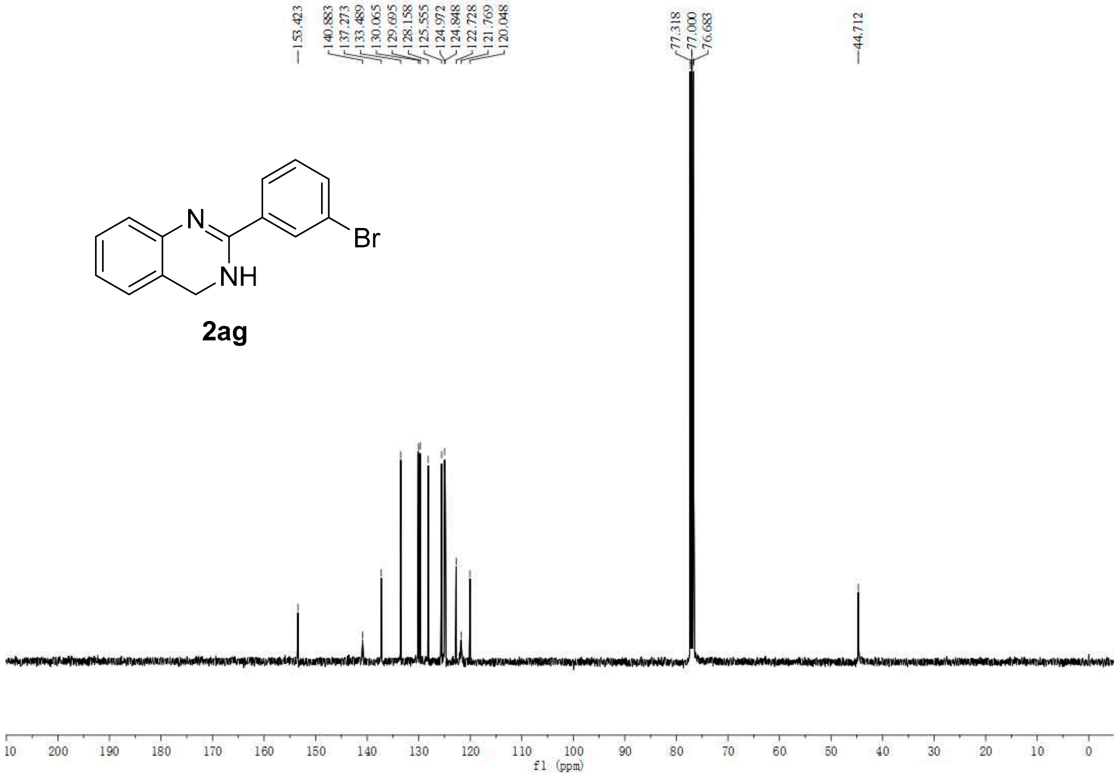

^{1}H NMR (400 MHz, $CDCl_3$) spectrum of **2ah**

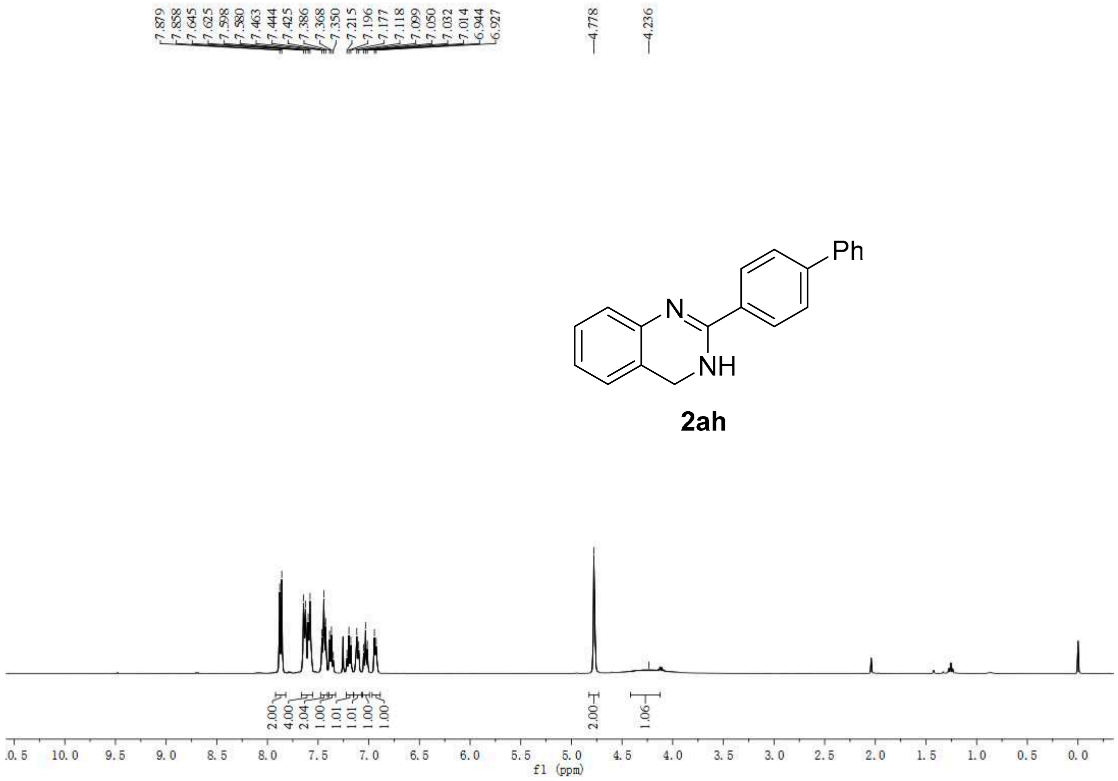

^{13}C {^{1}H} NMR (100 MHz, $CDCl_3$) spectrum of **2ah**

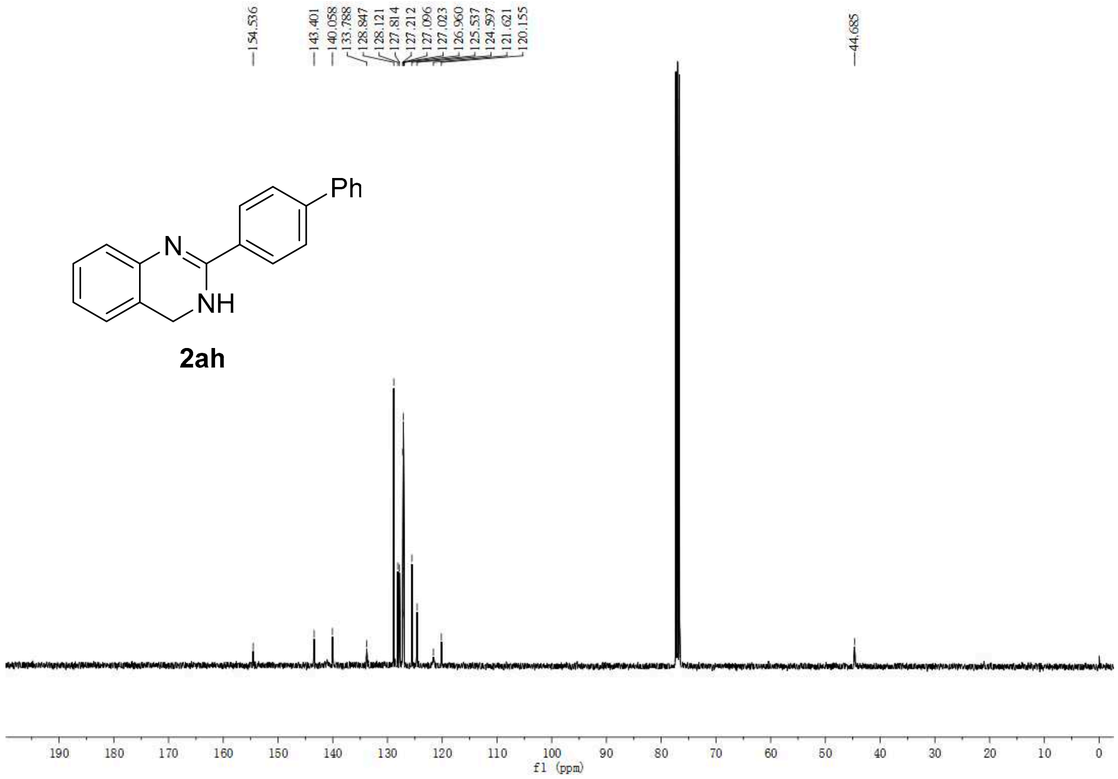

^{1}H NMR (400 MHz, $CDCl_3$) spectrum of **2ai**

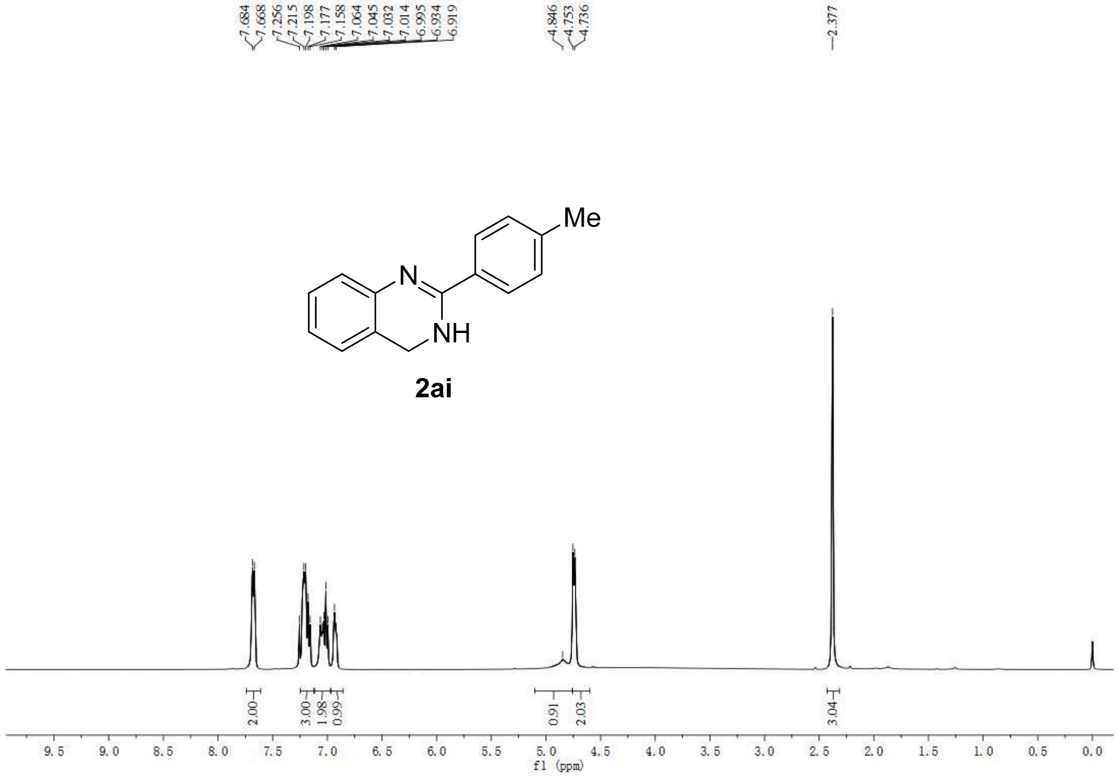

^{13}C {^{1}H} NMR (100 MHz, $CDCl_3$) spectrum of **2ai**

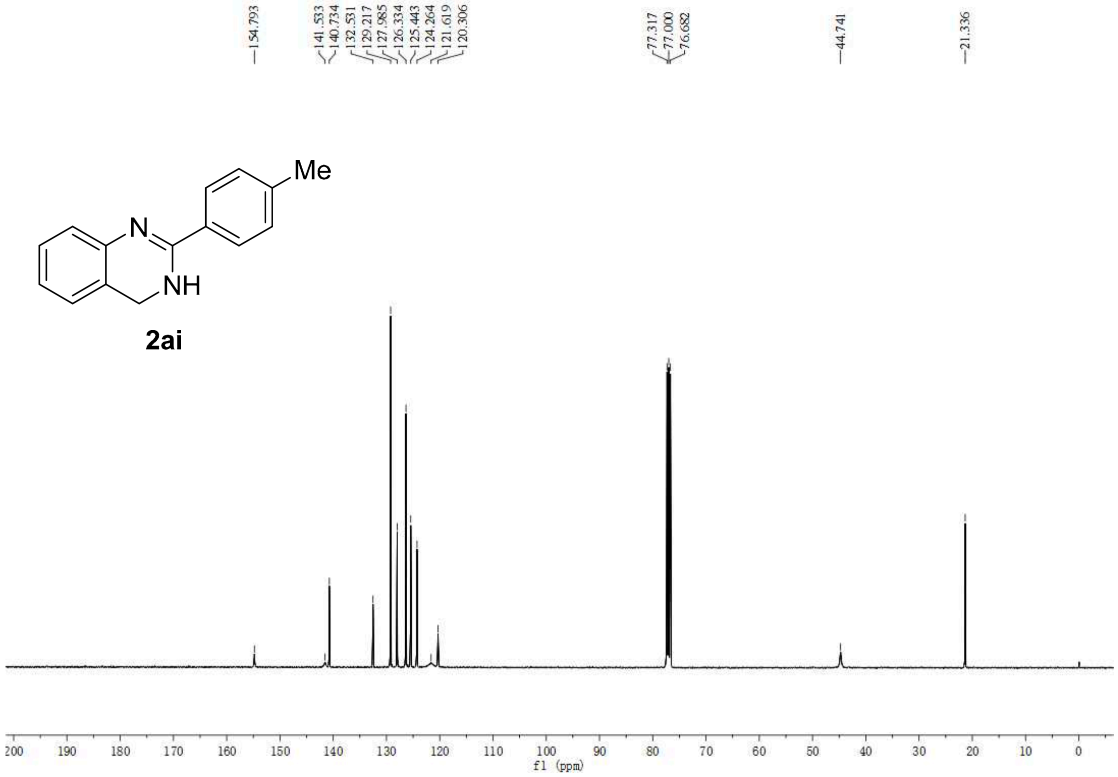

^{1}H NMR (400 MHz, $CDCl_3$) spectrum of **2aj**

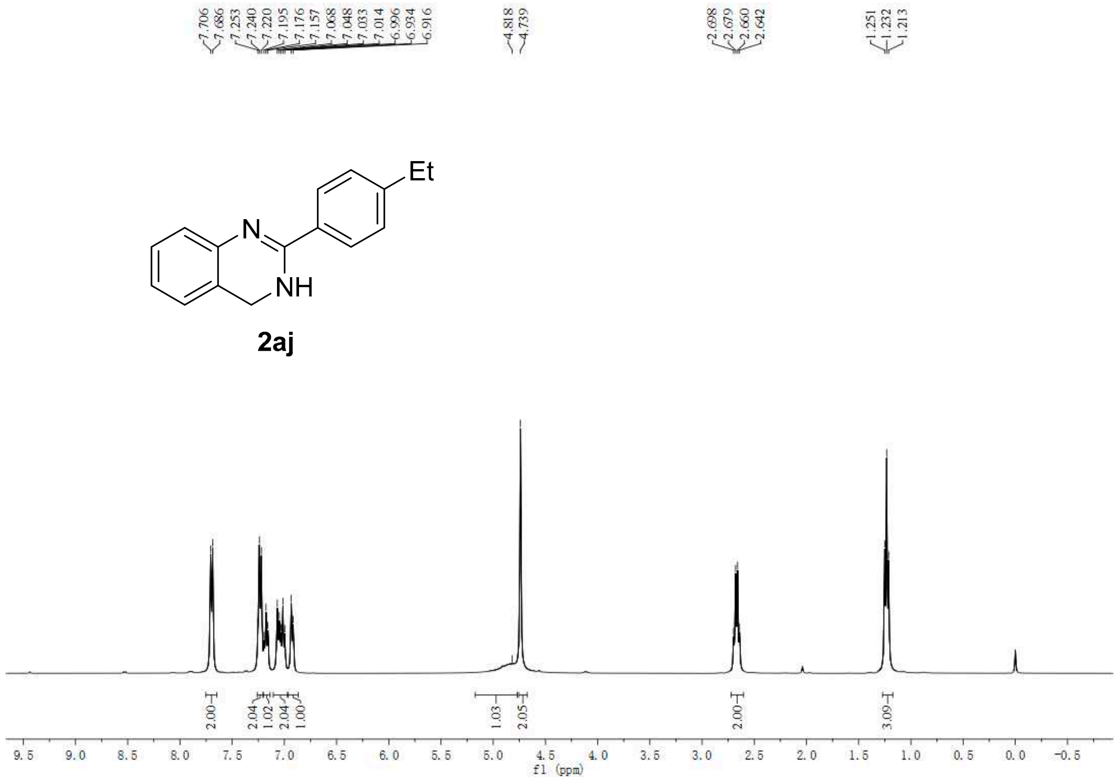

^{13}C {^{1}H} NMR (100 MHz, $CDCl_3$) spectrum of **2aj**

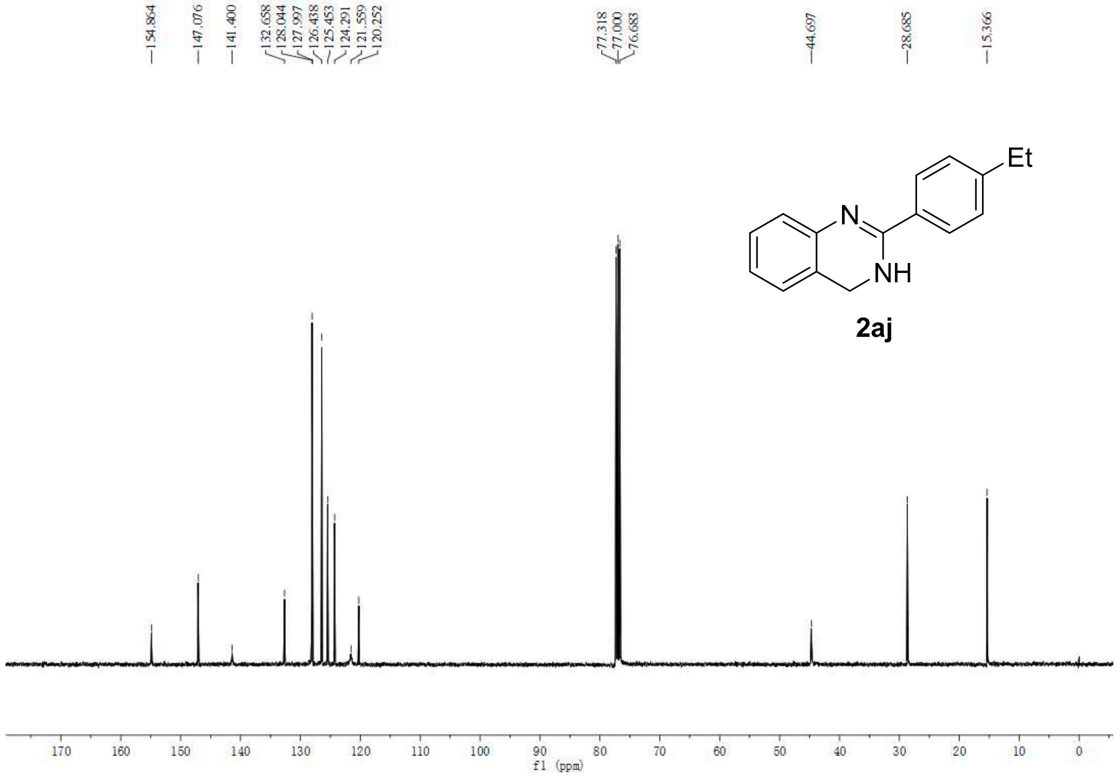

^{1}H NMR (400 MHz, $CDCl_3$) spectrum of **2ak**

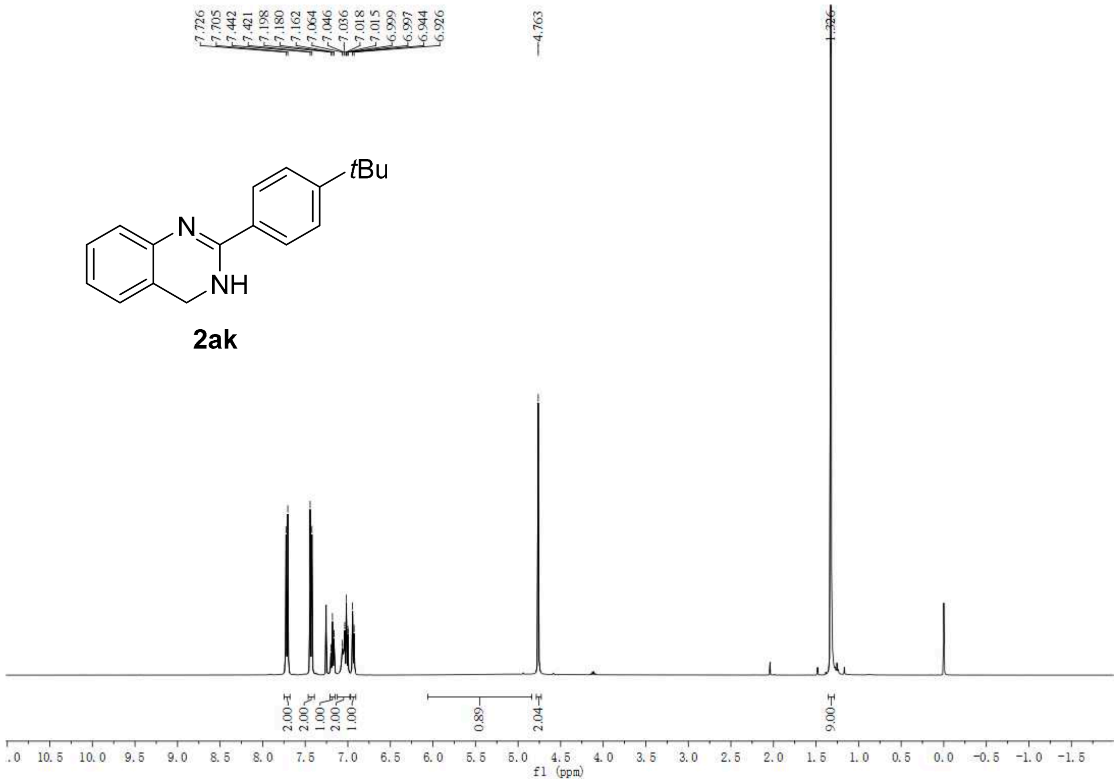

^{13}C {^{1}H} NMR (100 MHz, $CDCl_3$) spectrum of **2ak**

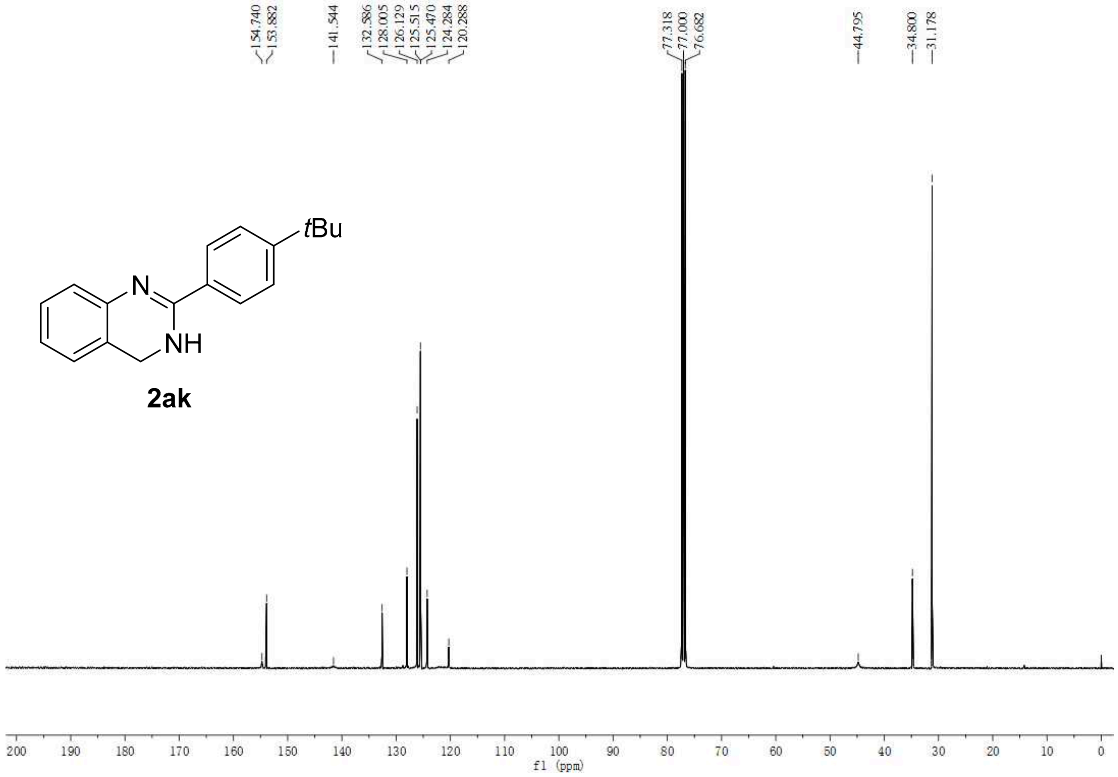

^{1}H NMR (400 MHz, $CDCl_3$) spectrum of **2al**

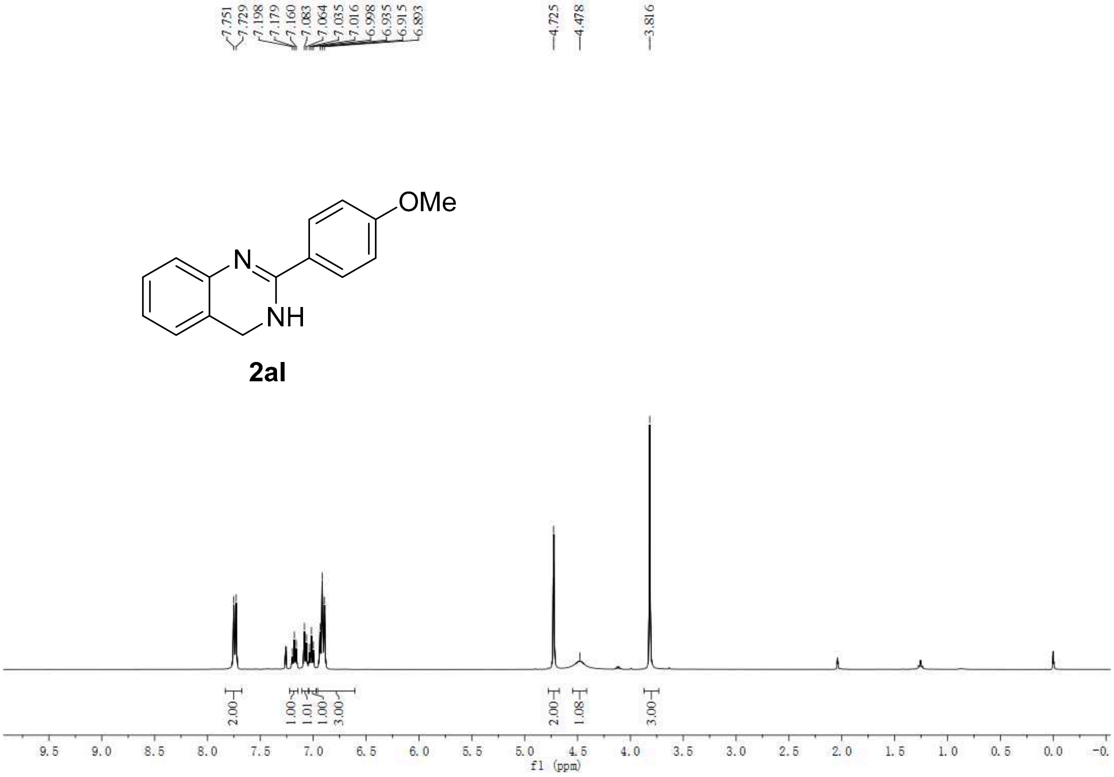

^{13}C {^{1}H} NMR (100 MHz, $CDCl_3$) spectrum of **2al**

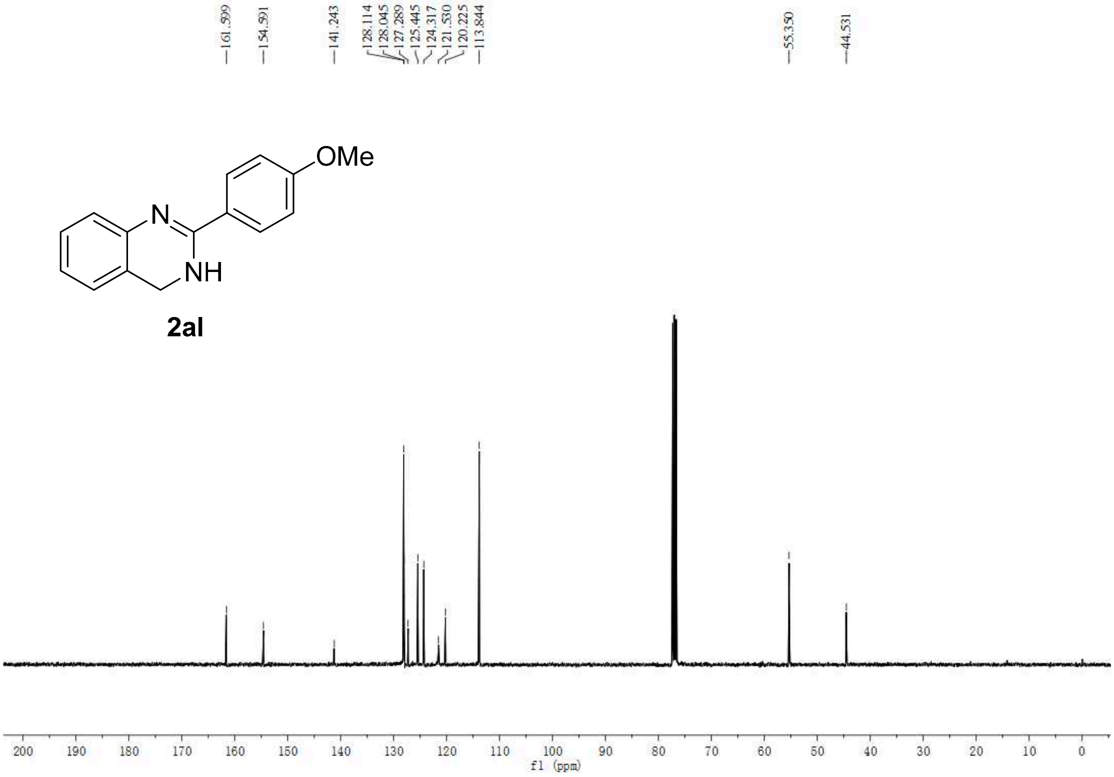

^{1}H NMR (400 MHz, $CDCl_3$) spectrum of **2am**

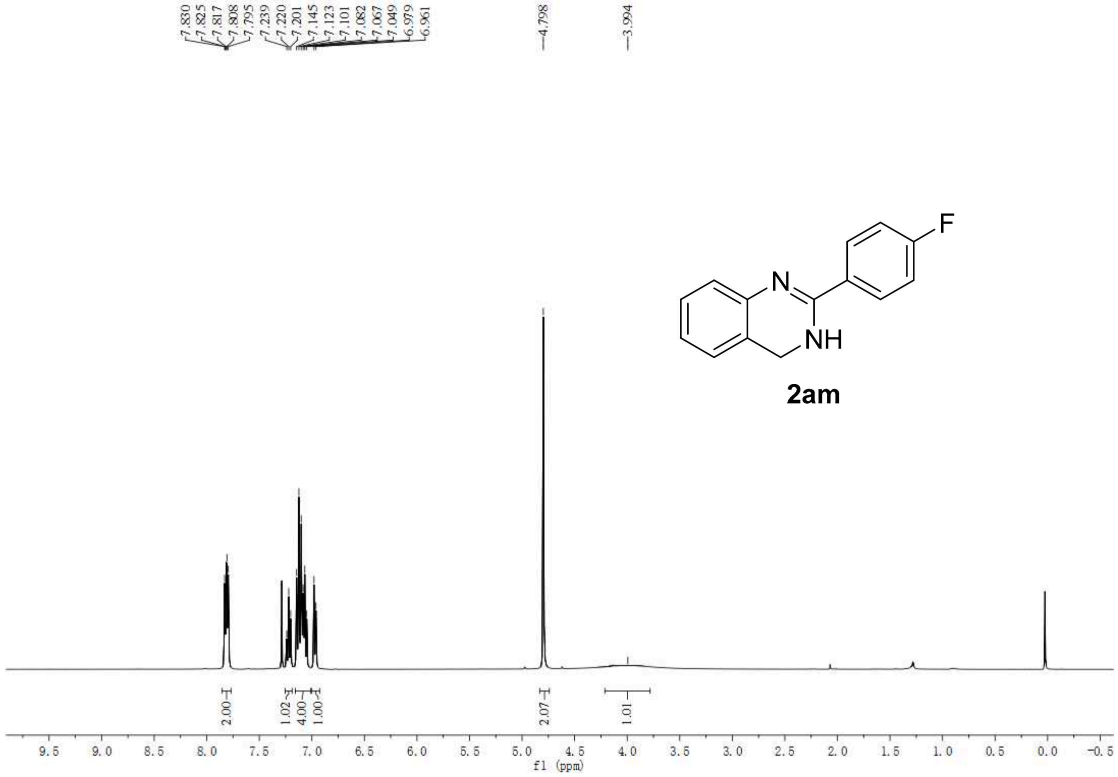

^{13}C {^{1}H} NMR (100 MHz, $CDCl_3$) spectrum of **2am**

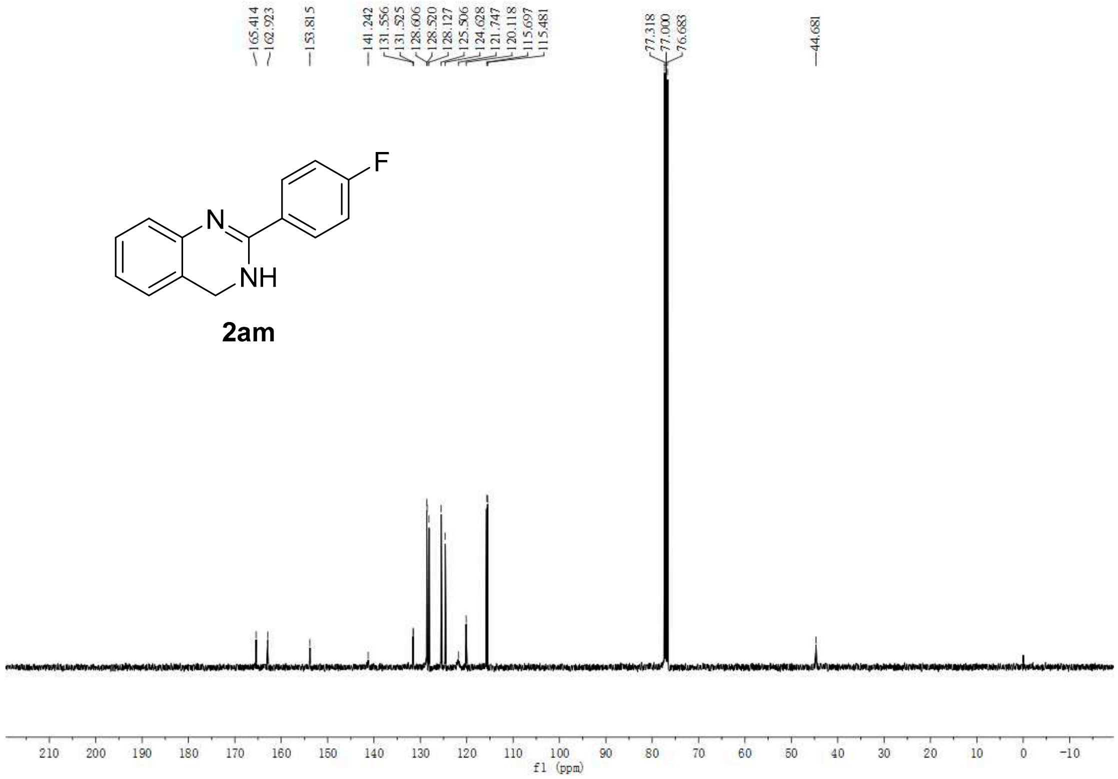

^{19}F (376 MHz, CDCl$_3$) spectrum of **2am**

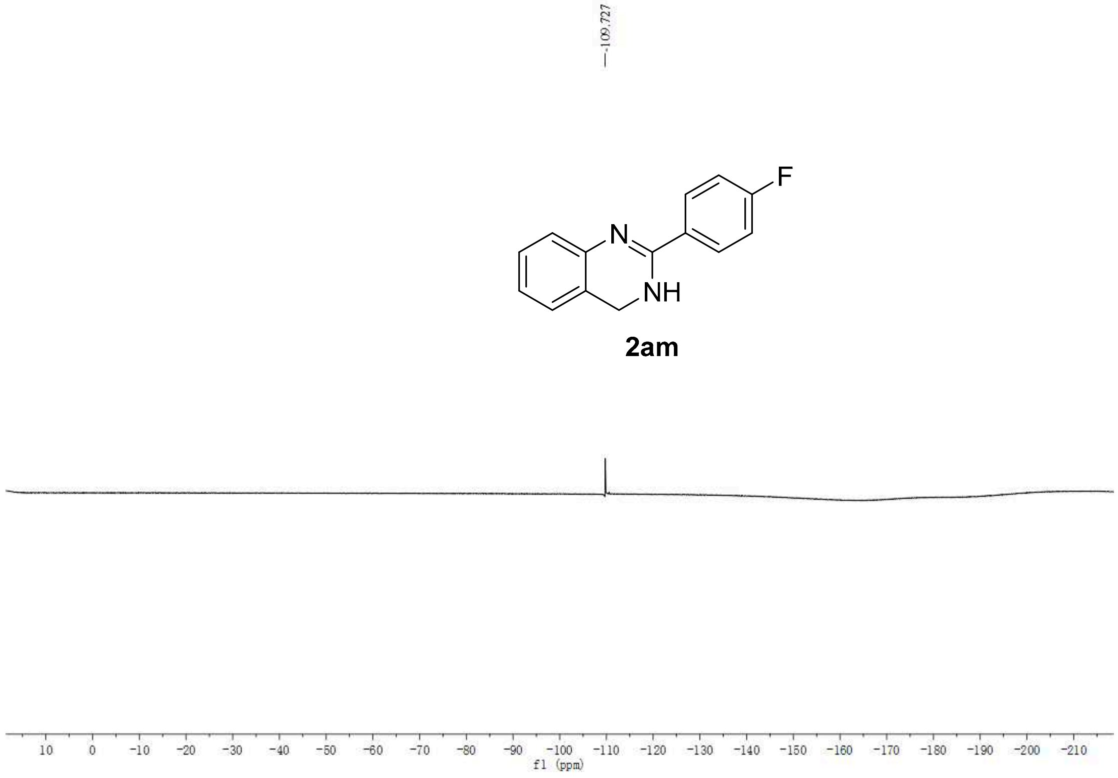

^{1}H NMR (400 MHz, CDCl$_3$) spectrum of **2an**

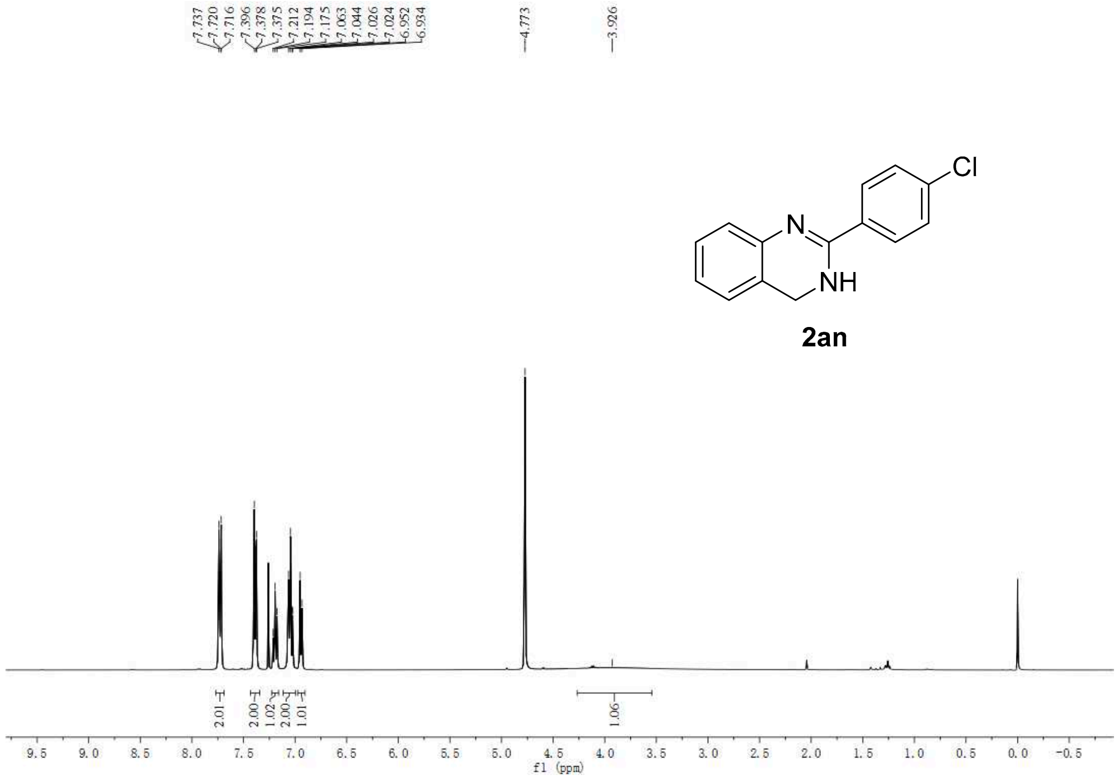

^{13}C {^{1}H} NMR (100 MHz, $CDCl_3$) spectrum of **2an**

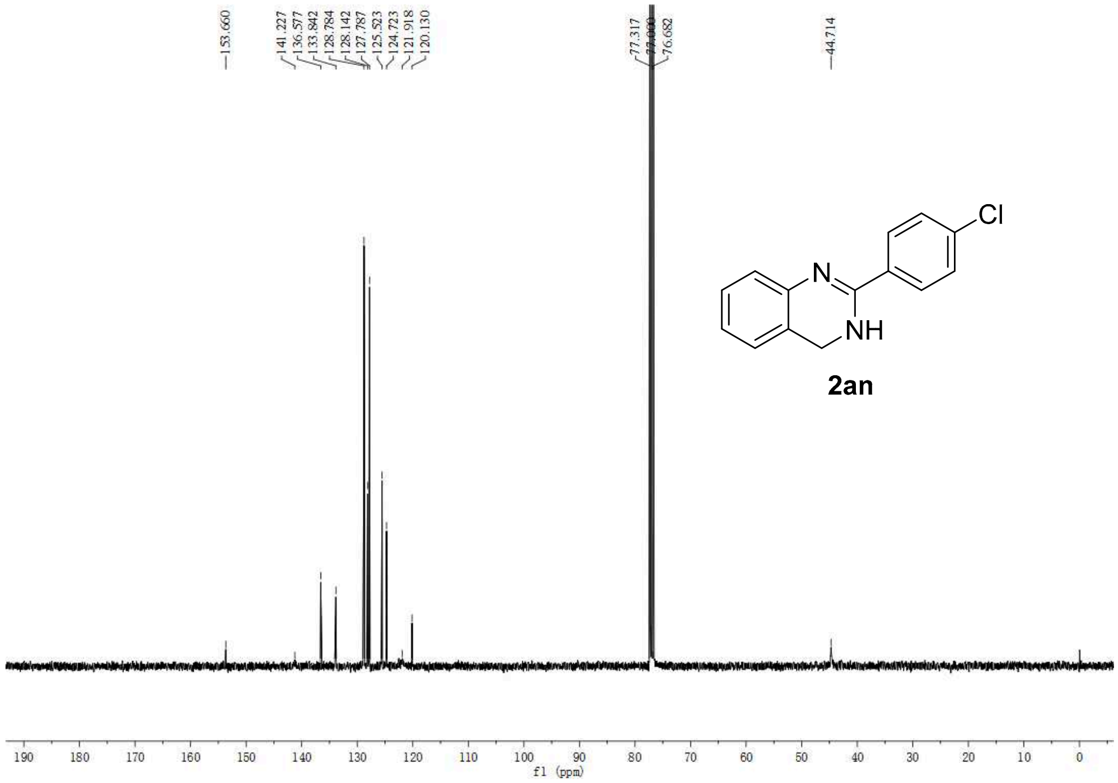

^{1}H NMR (400 MHz, $CDCl_3$) spectrum of **2ao**

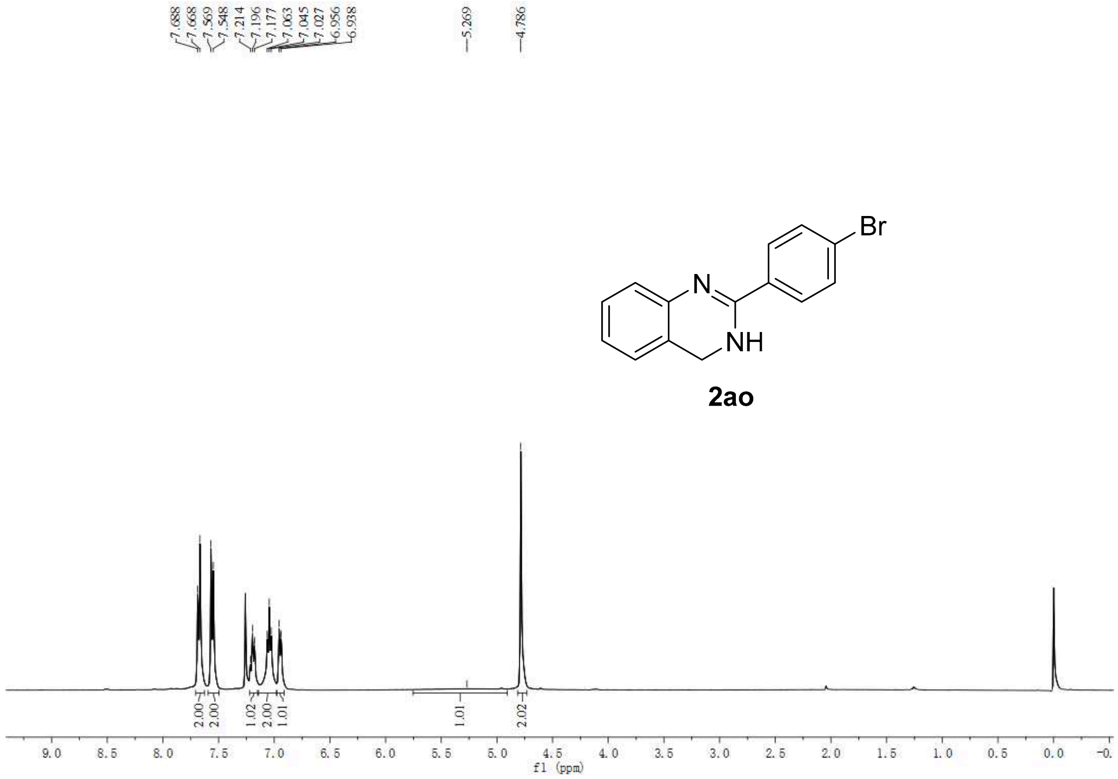

^{13}C {^{1}H} NMR (100 MHz, $CDCl_3$) spectrum of **2ao**

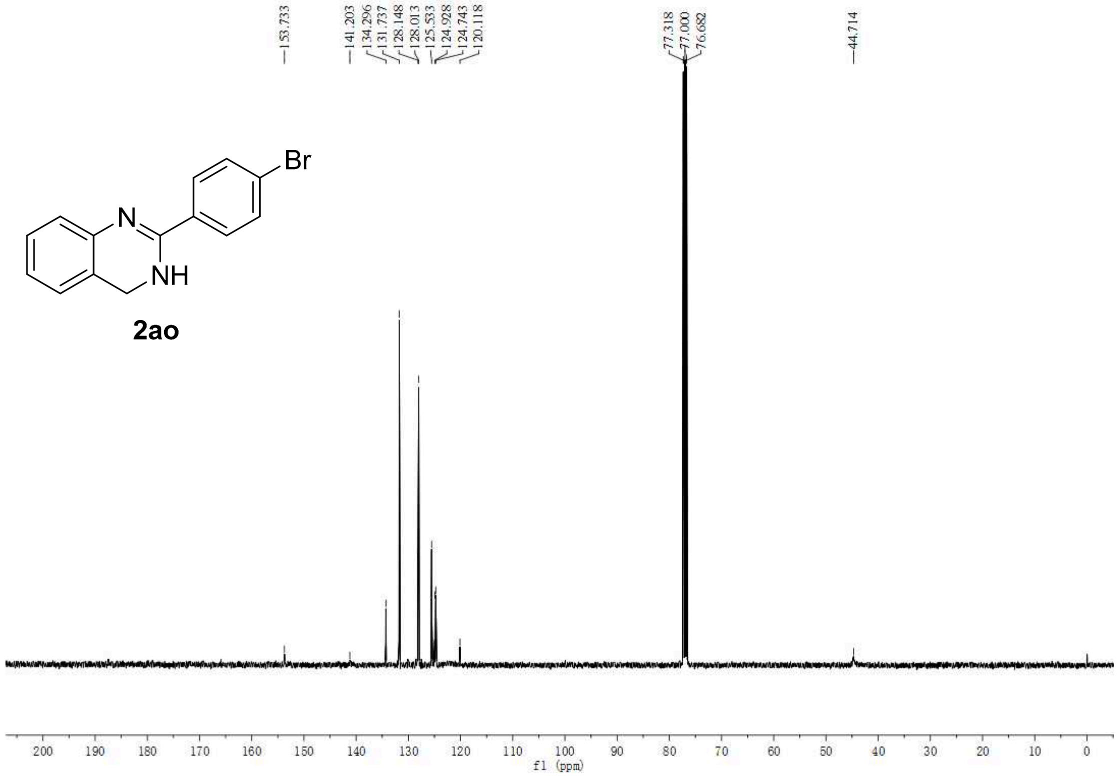

^{1}H NMR (400 MHz, $CDCl_3$) spectrum of **2ap**

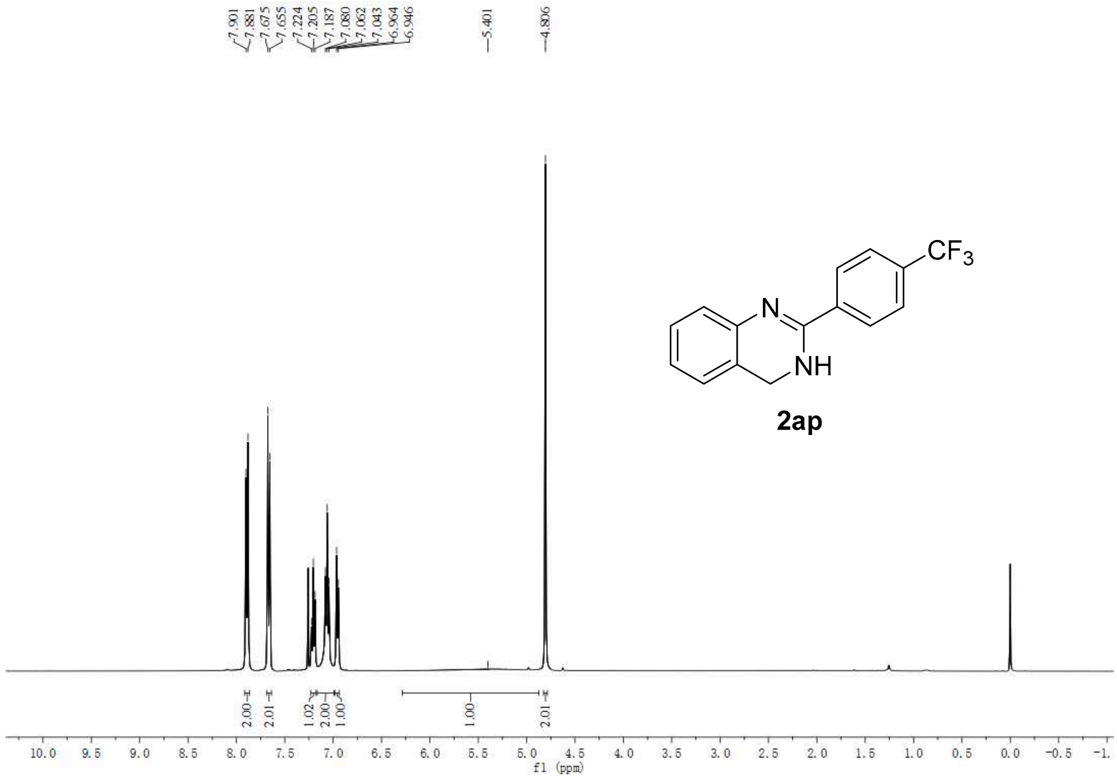

^{13}C {^{1}H} NMR (100 MHz, $CDCl_3$) spectrum of **2ap**

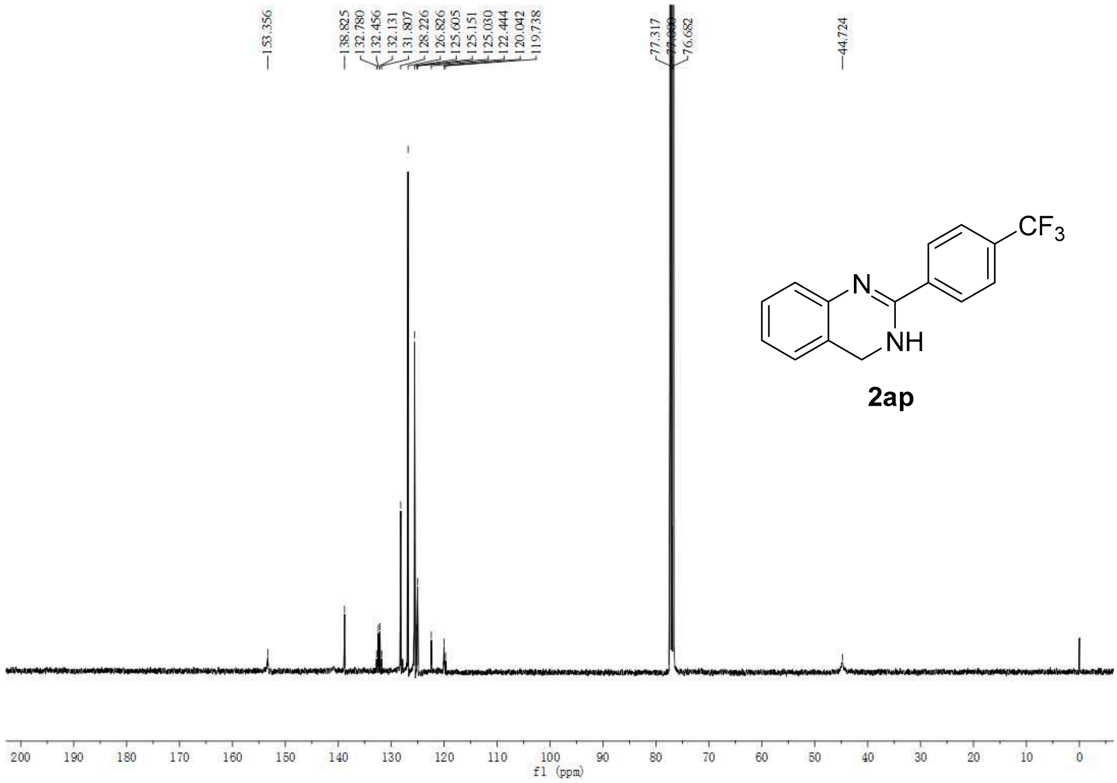

^{19}F (376 MHz, $CDCl_3$) spectrum of **2ap**

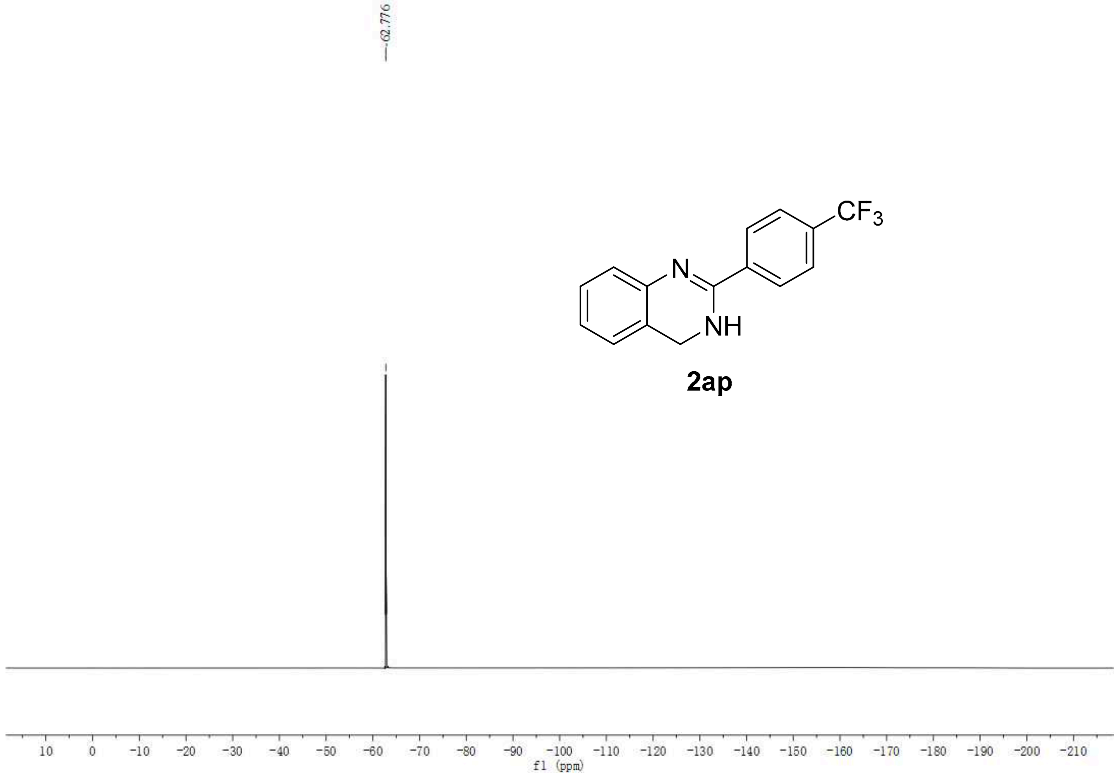

^{1}H NMR (400 MHz, $CDCl_3$) spectrum of **2aq**

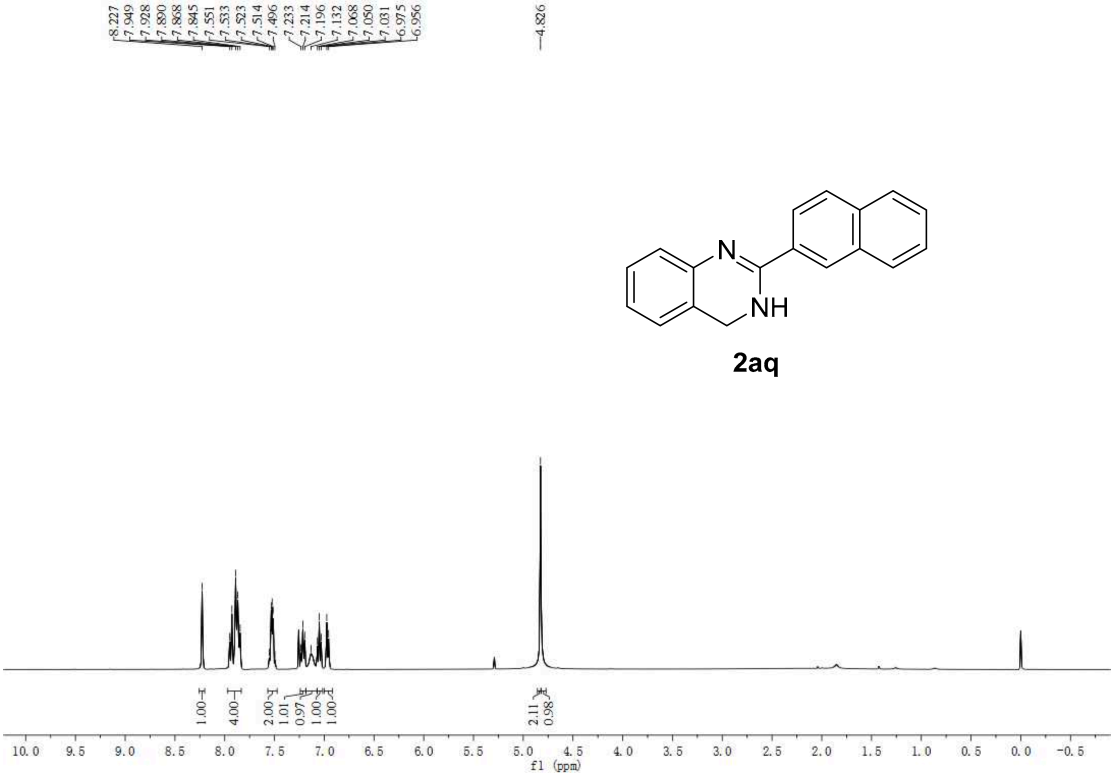

^{13}C {^{1}H} NMR (100 MHz, $CDCl_3$) spectrum of **2aq**

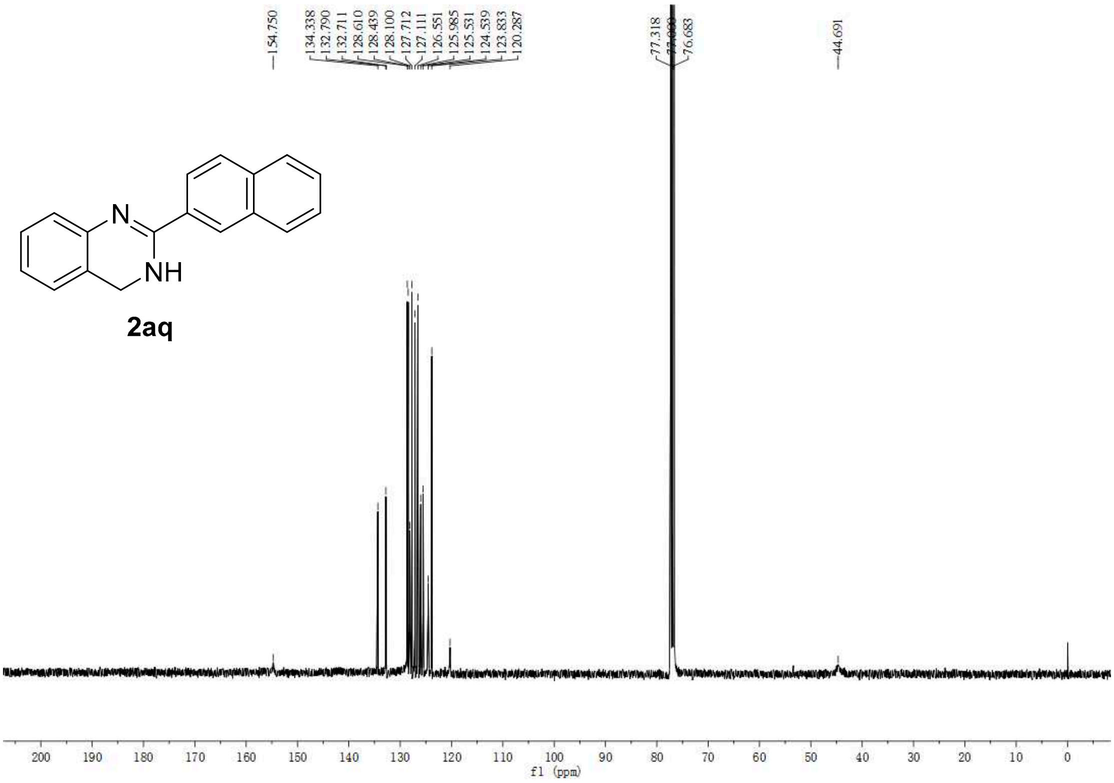

^{1}H NMR (400 MHz, $CDCl_3$) spectrum of **2ar**

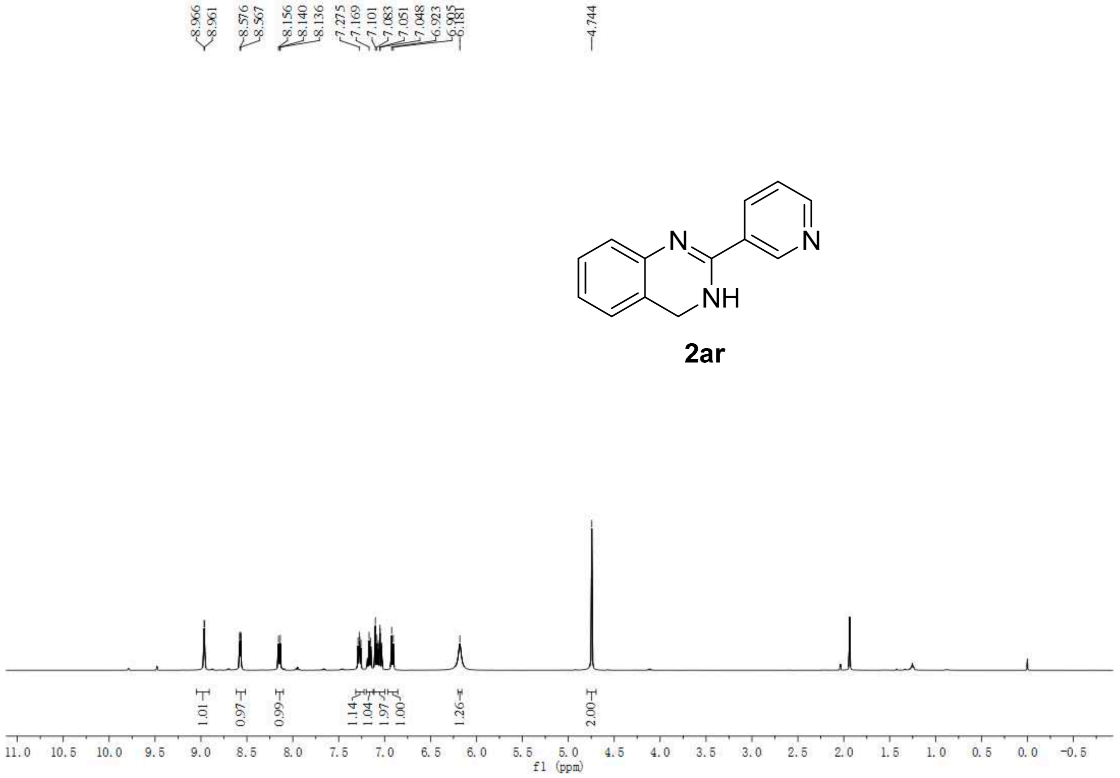

^{13}C {^{1}H} NMR (100 MHz, $CDCl_3$) spectrum of **2ar**

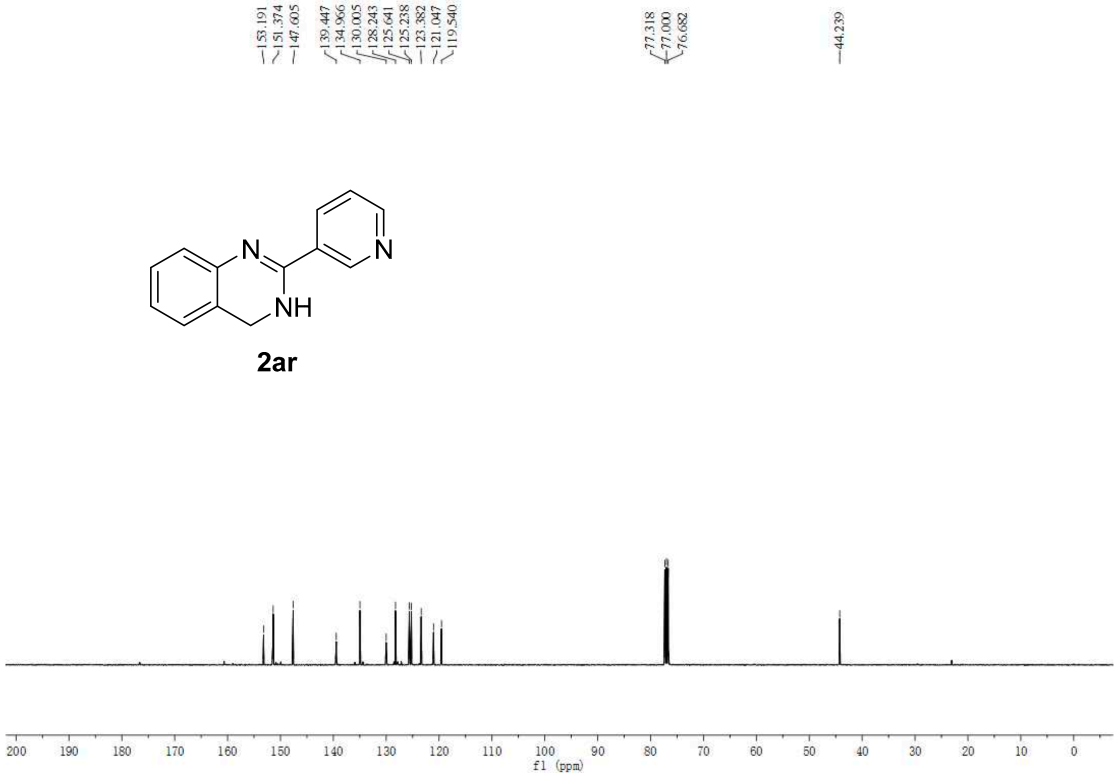

^{1}H NMR (400 MHz, $CDCl_3$) spectrum of **2as**

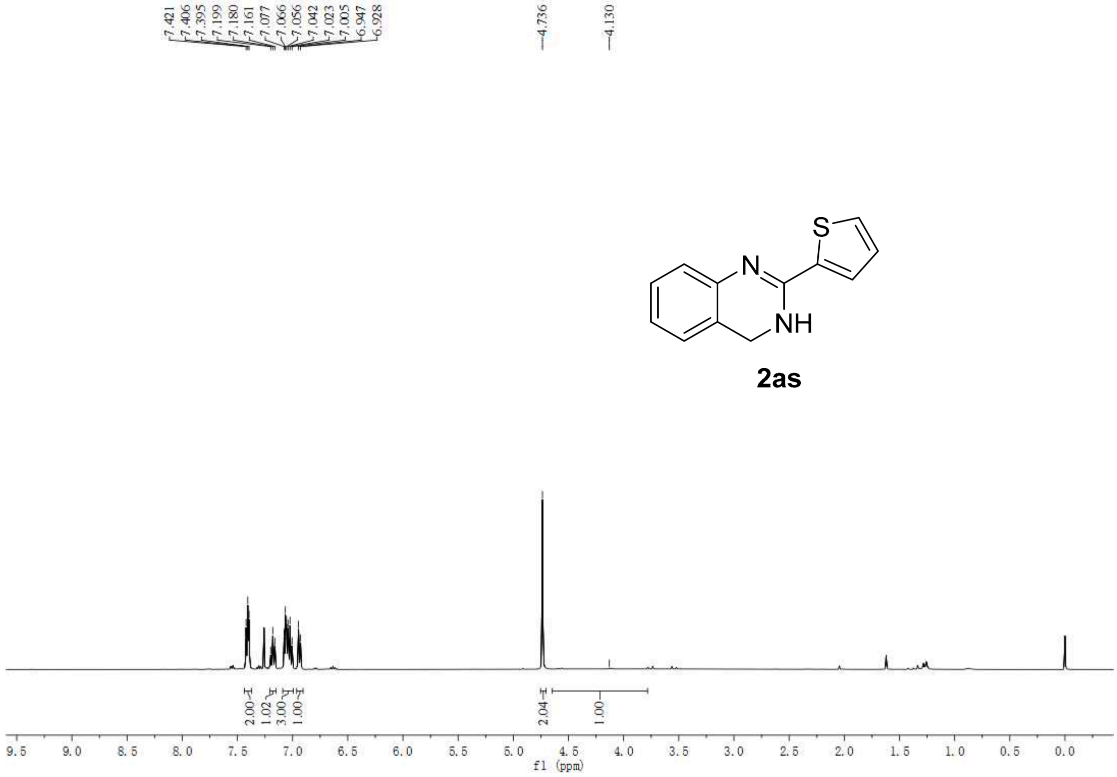

^{13}C {^{1}H} NMR (100 MHz, $CDCl_3$) spectrum of **2as**

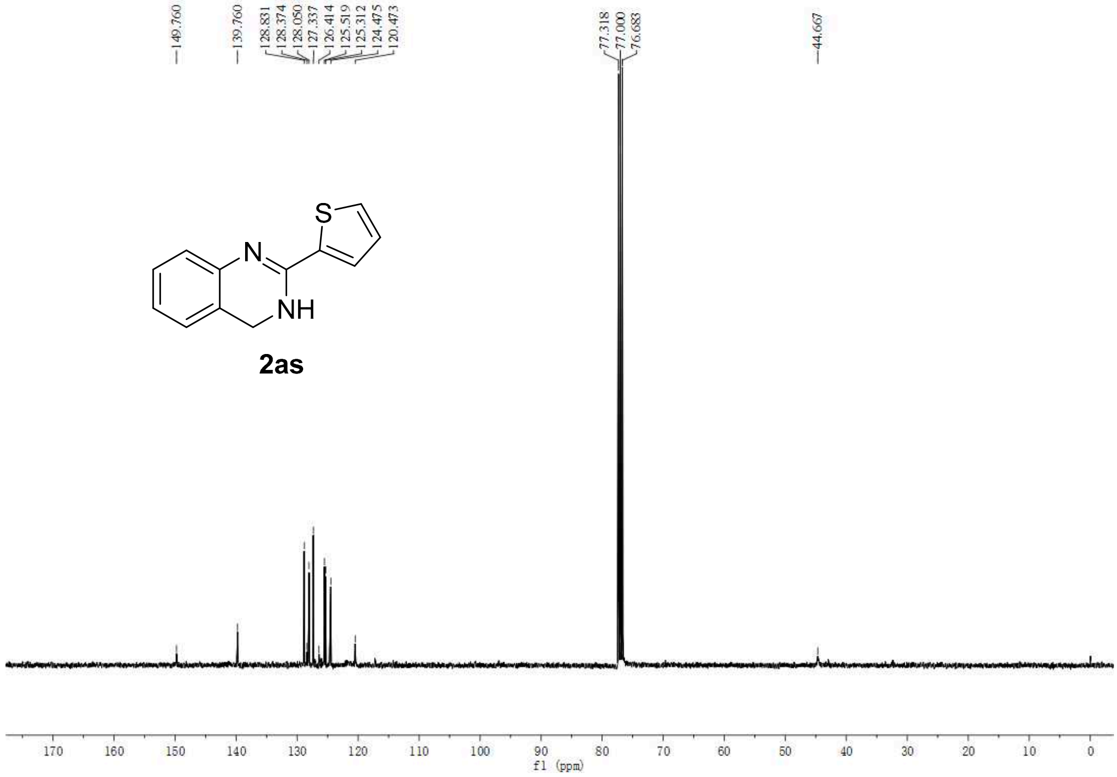

^{1}H NMR (400 MHz, $CDCl_3$) spectrum of **2at**

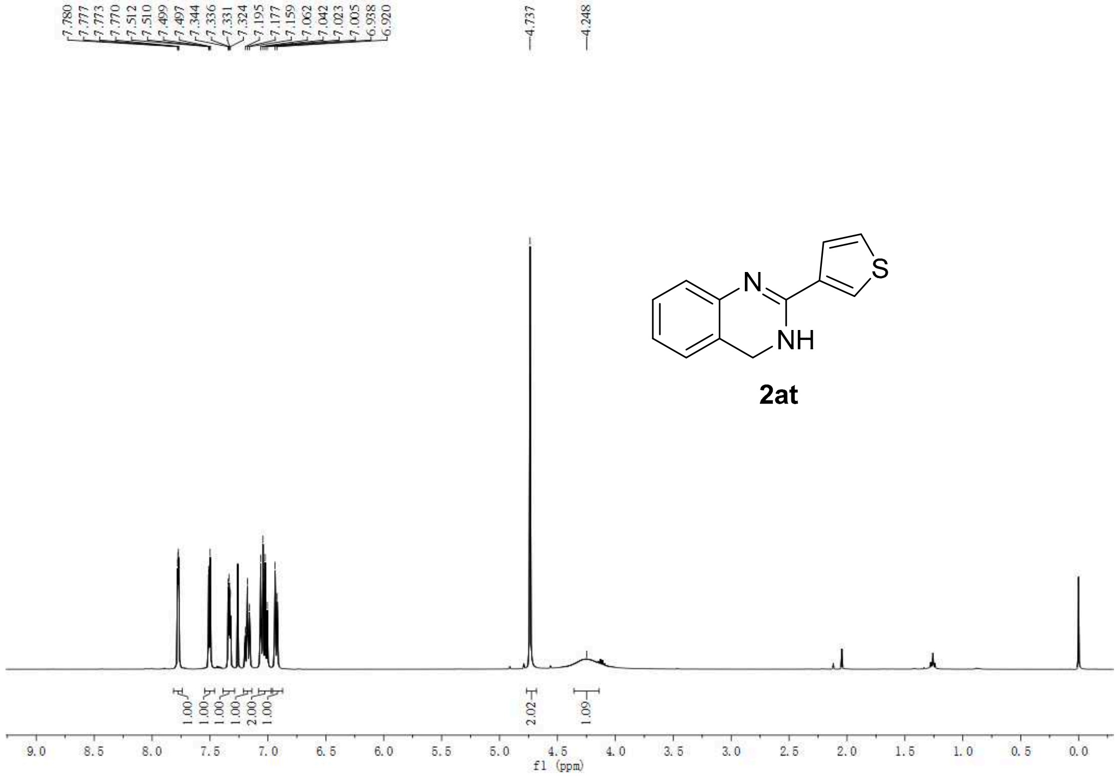

^{13}C {^{1}H} NMR (100 MHz, $CDCl_3$) spectrum of **2at**

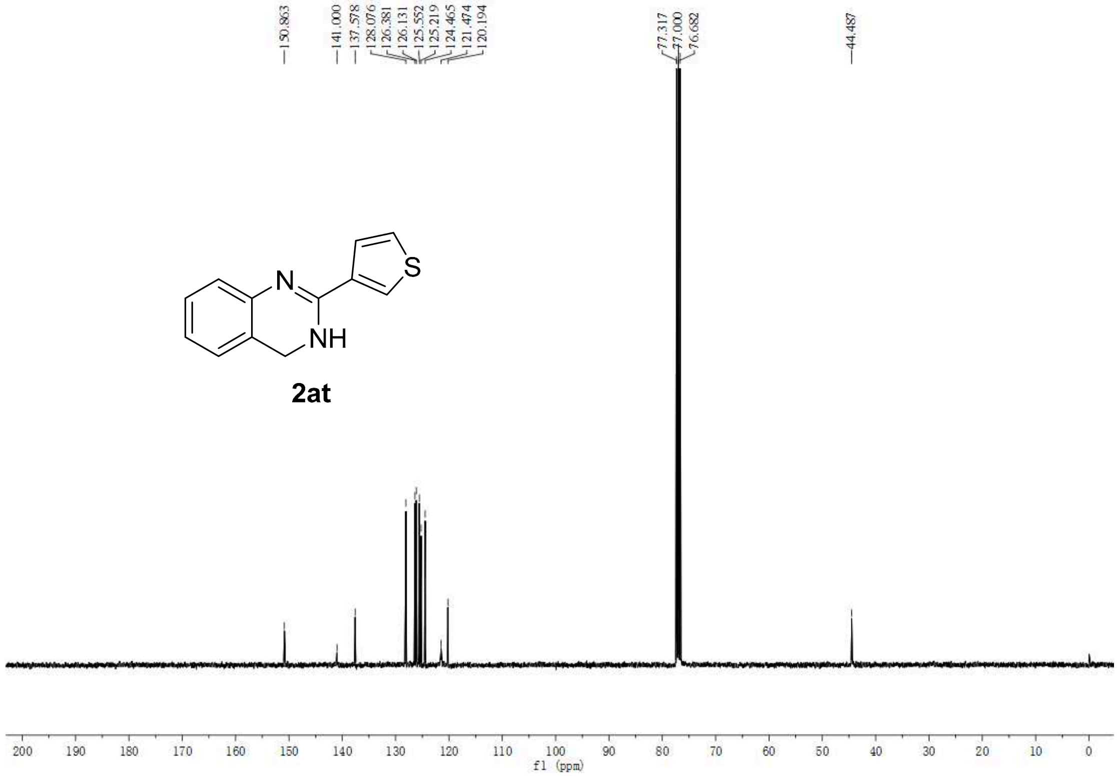

^{1}H NMR (400 MHz, $CDCl_3$) spectrum of **2au**

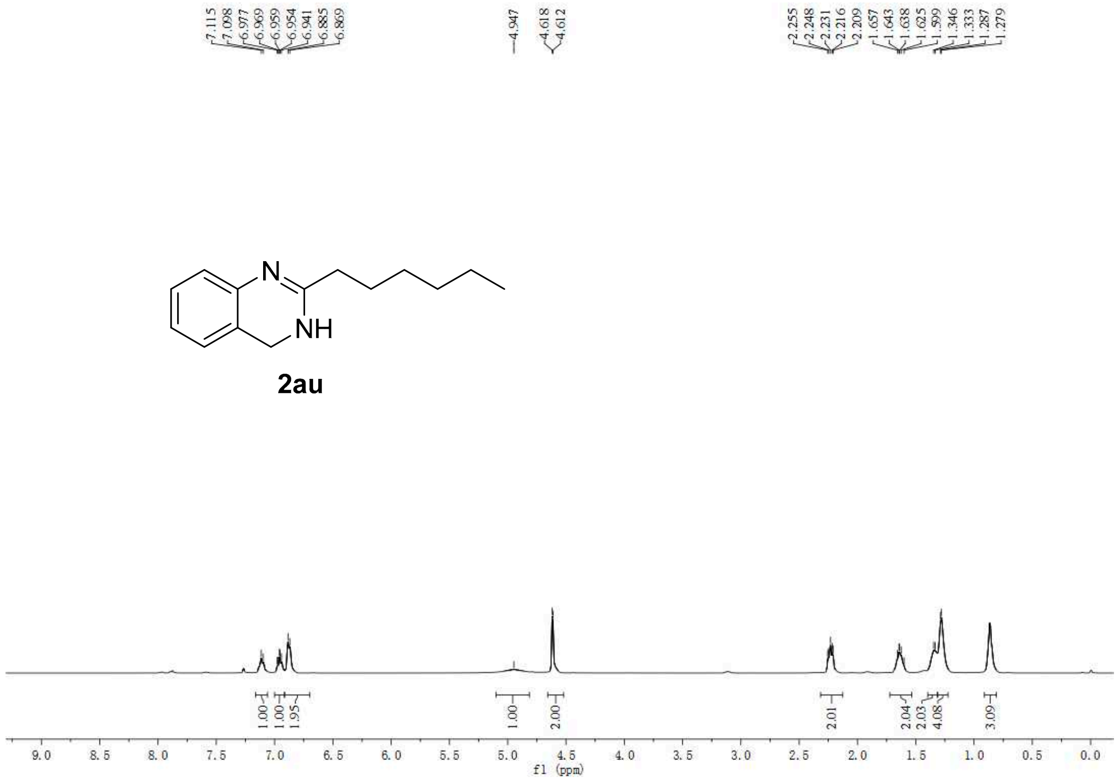

^{13}C {^{1}H} NMR (100 MHz, $CDCl_3$) spectrum of **2au**

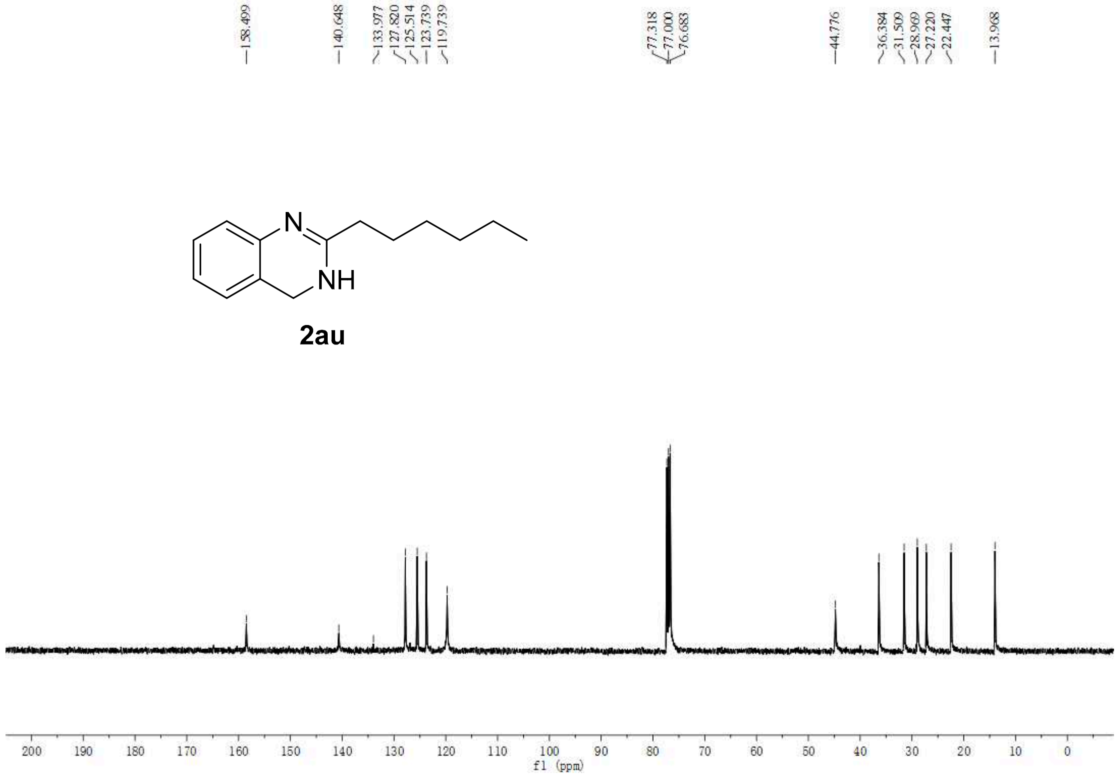

^{1}H NMR (400 MHz, $CDCl_3$) spectrum of **2av**

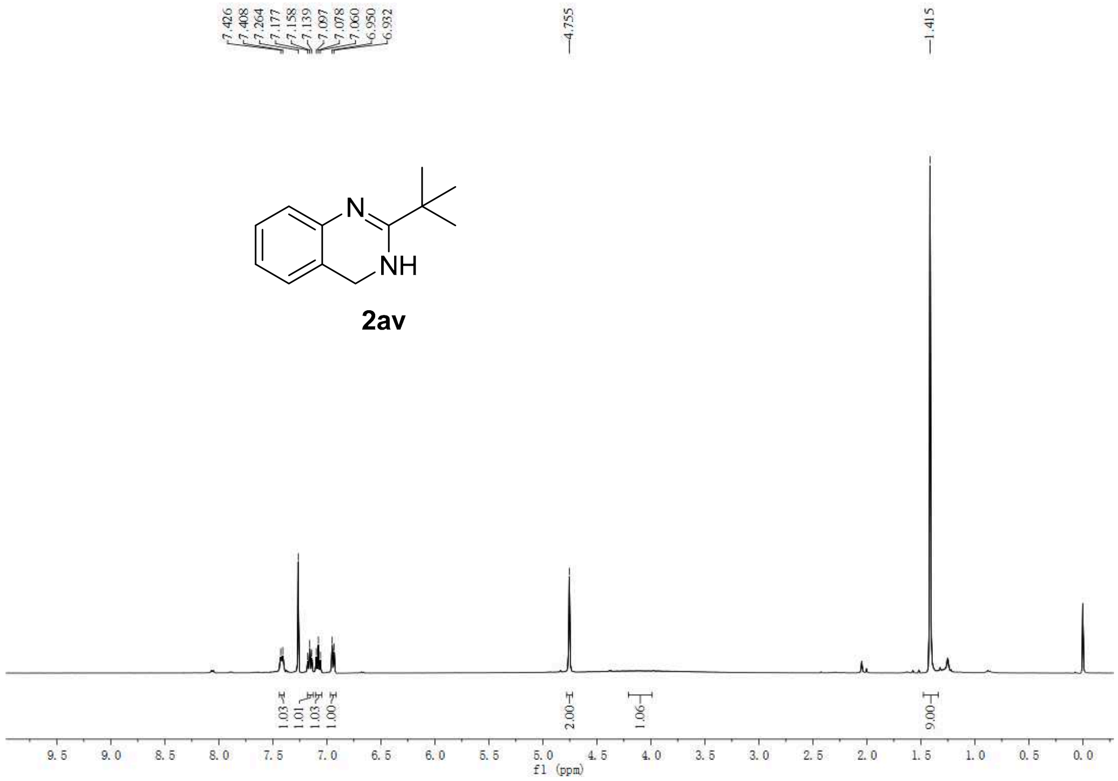

^{13}C {^{1}H} NMR (100 MHz, $CDCl_3$) spectrum of **2av**

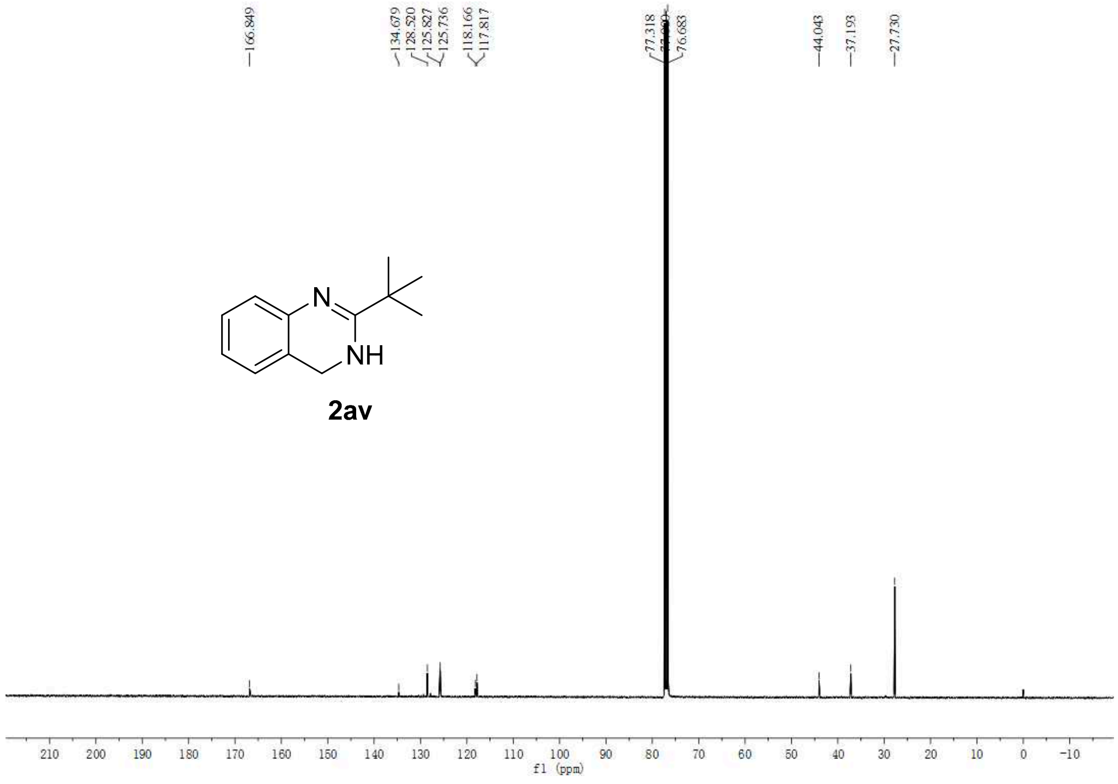

^{1}H NMR (400 MHz, $CDCl_3$) spectrum of **2ba**

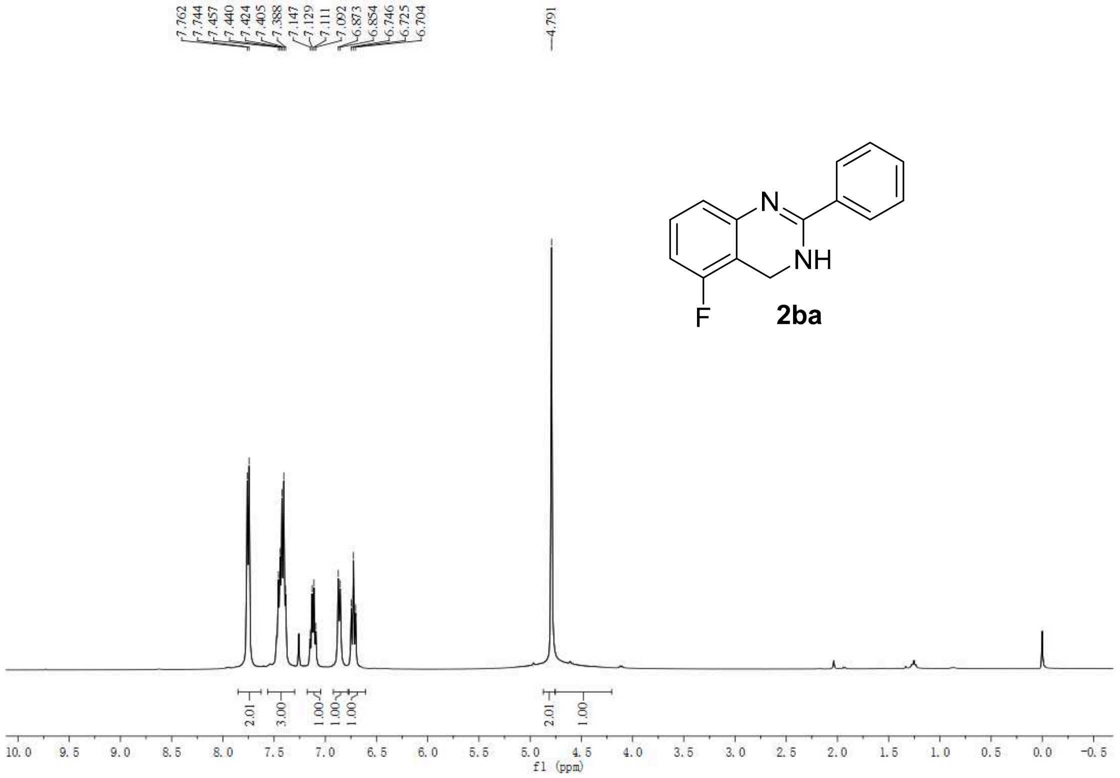

^{13}C {^{1}H} NMR (100 MHz, $CDCl_3$) spectrum of **2ba**

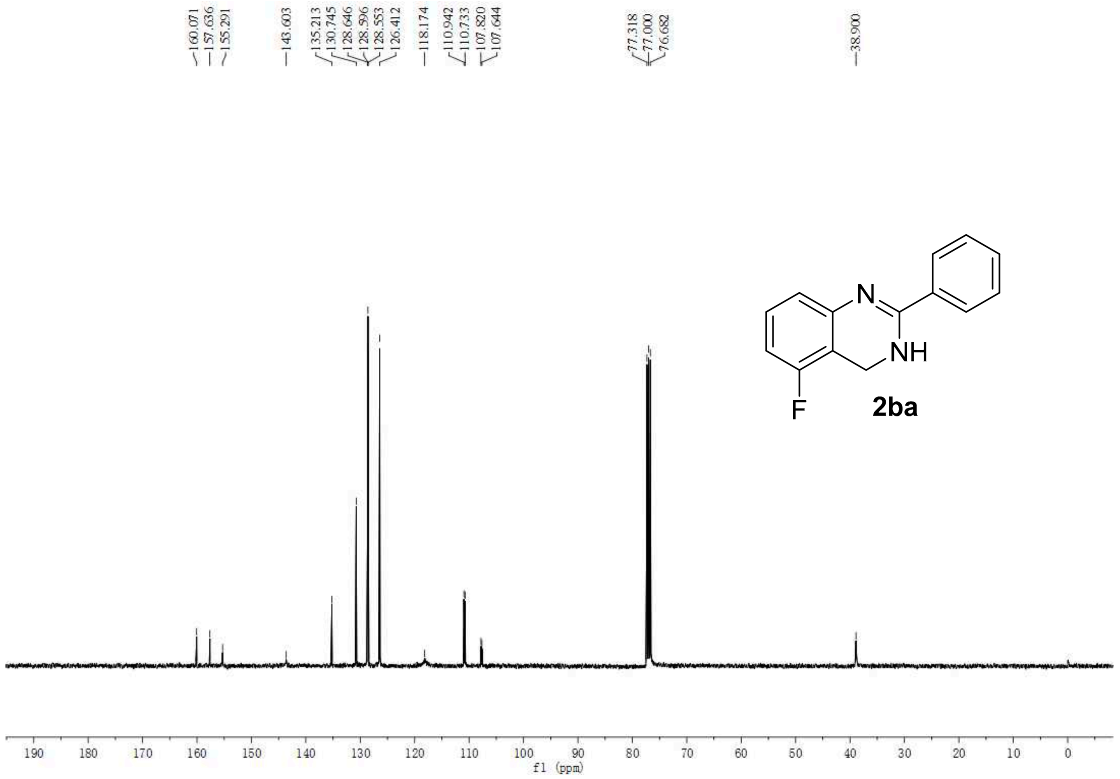

^{1}H NMR (400 MHz, $CDCl_3$) spectrum of **2ca**

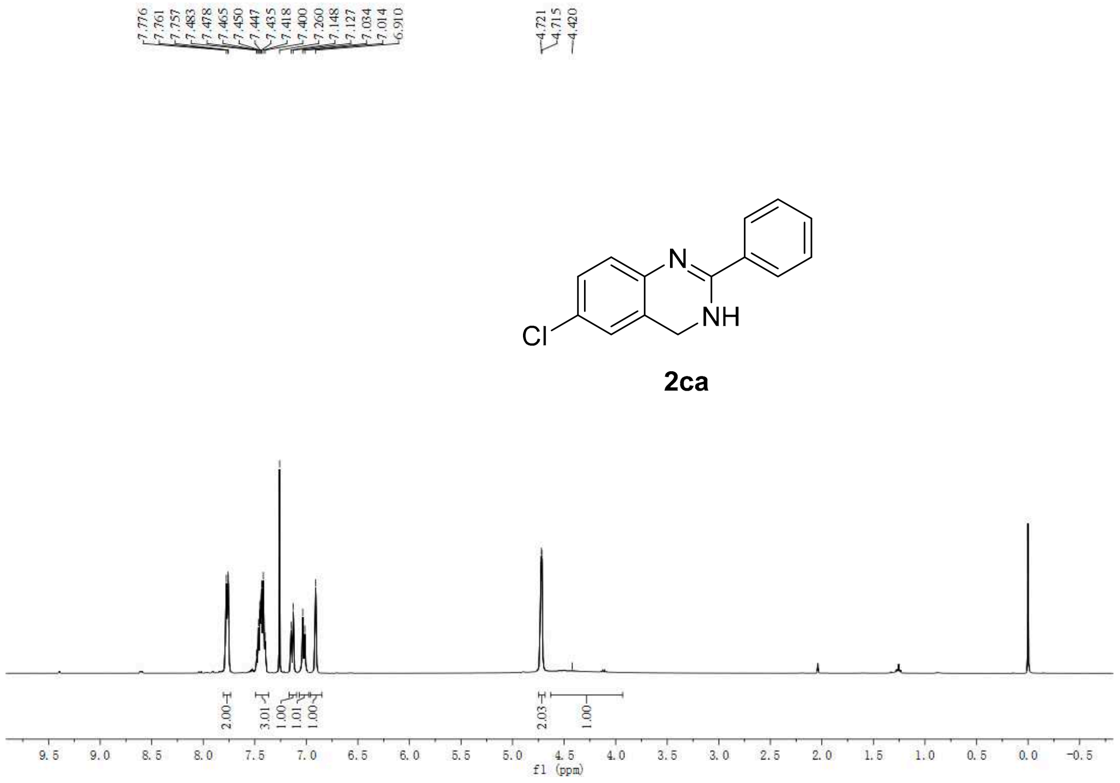

^{13}C {^{1}H} NMR (100 MHz, $CDCl_3$) spectrum of **2ca**

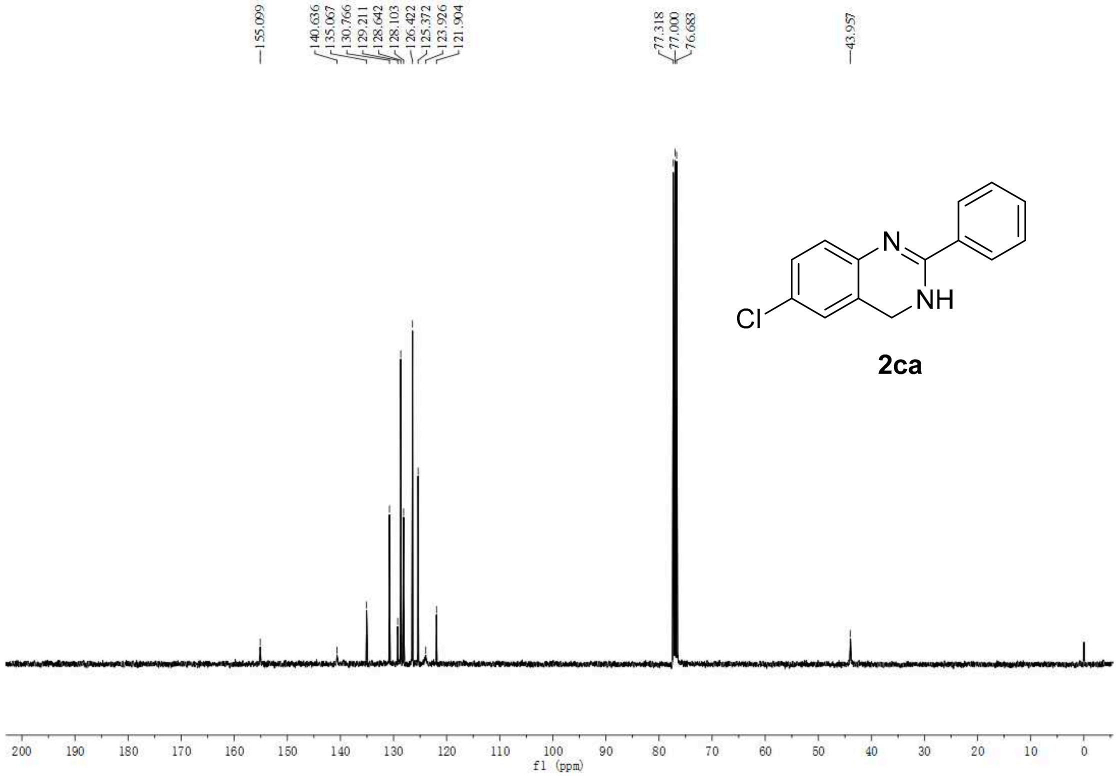

^{1}H NMR (400 MHz, $CDCl_3$) spectrum of **2da**

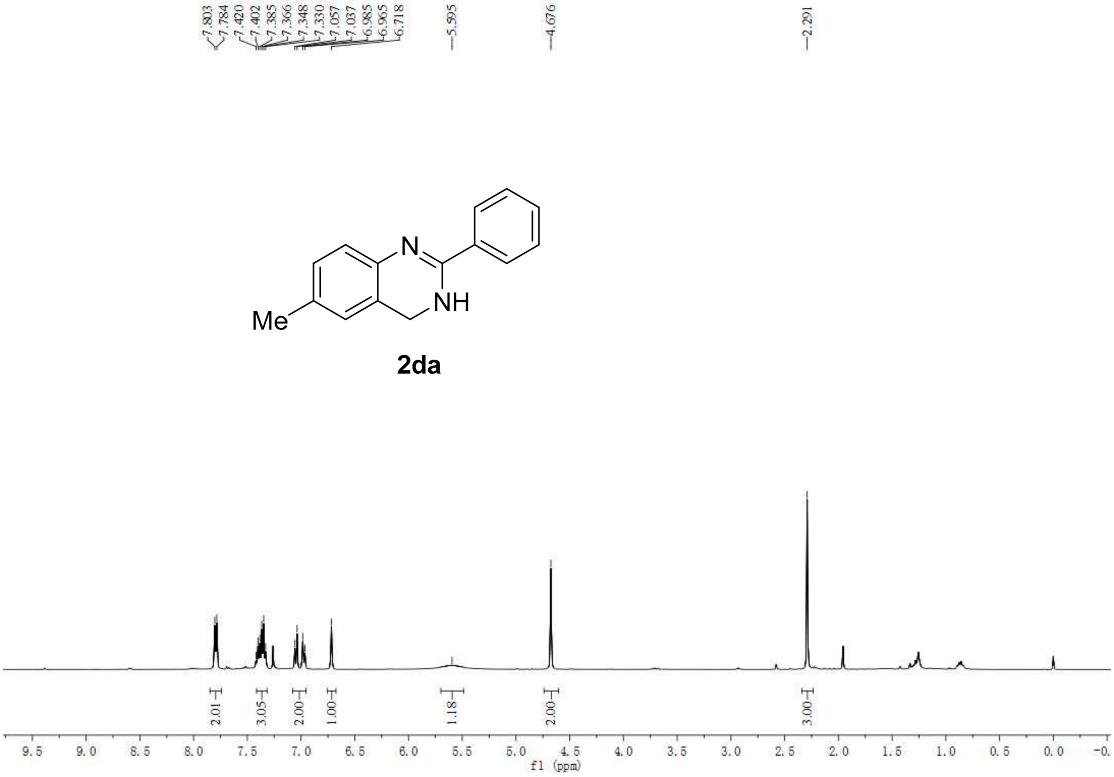

^{13}C {^{1}H} NMR (100 MHz, $CDCl_3$) spectrum of **2da**

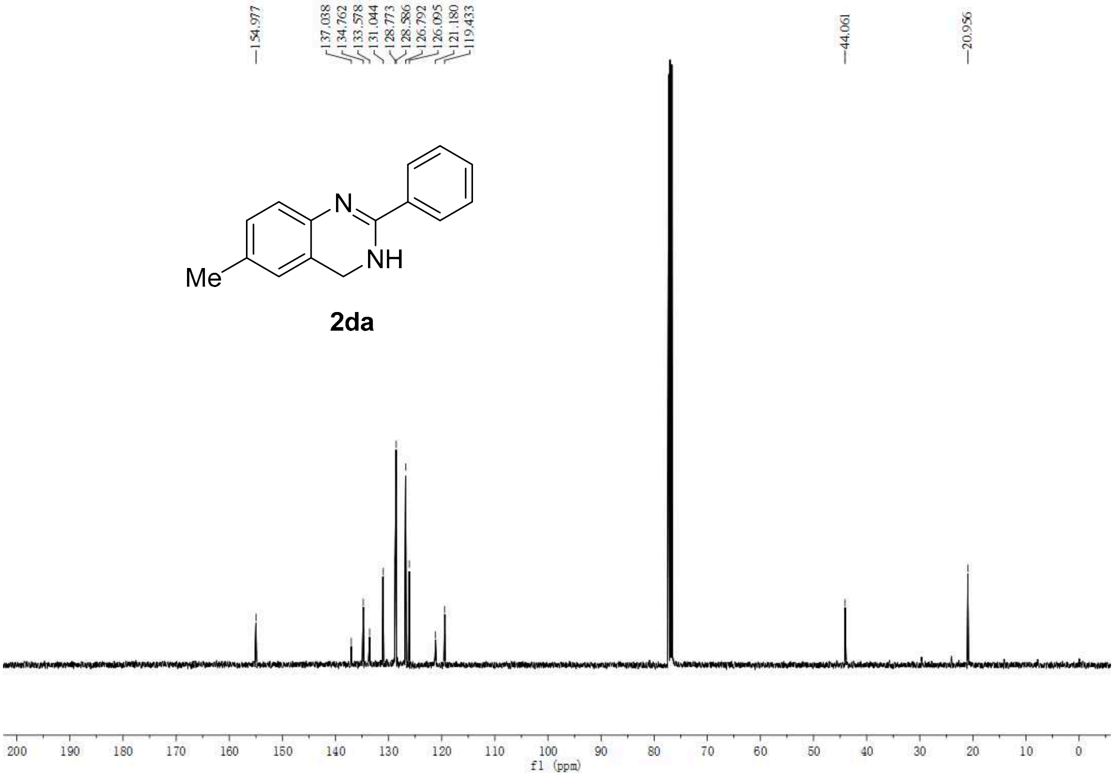

^{1}H NMR (400 MHz, $CDCl_3$) spectrum of **2ea**

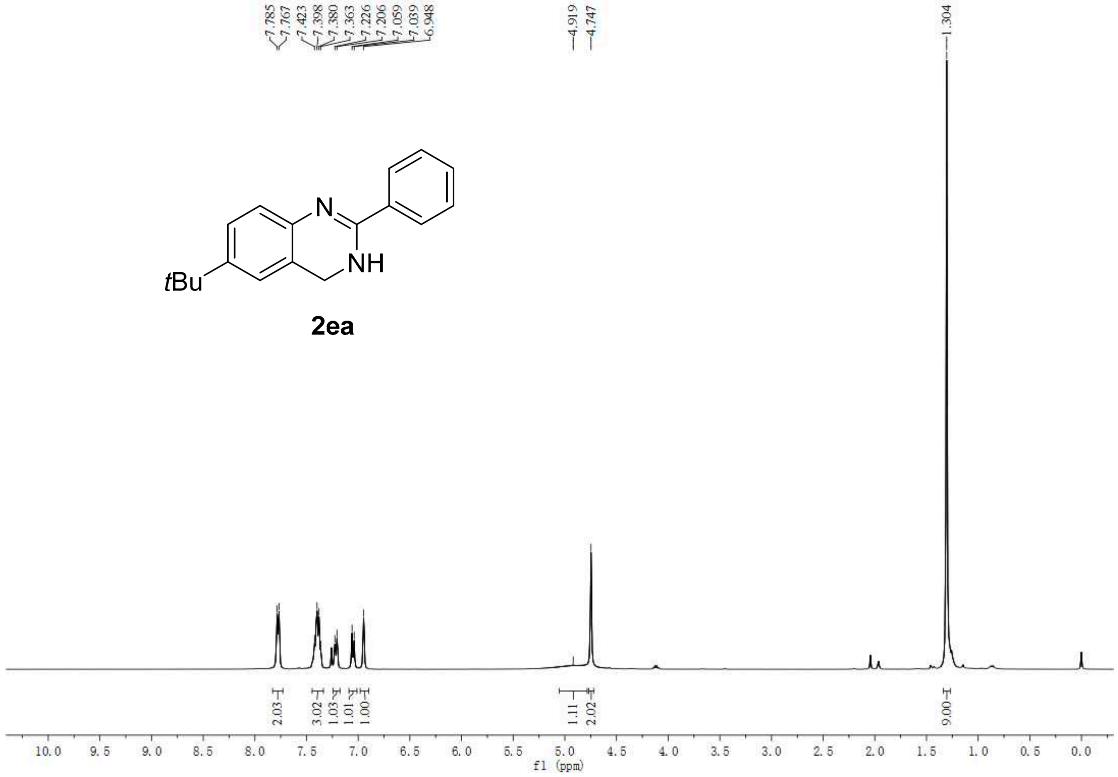

^{13}C {^{1}H} NMR (100 MHz, $CDCl_3$) spectrum of **2ea**

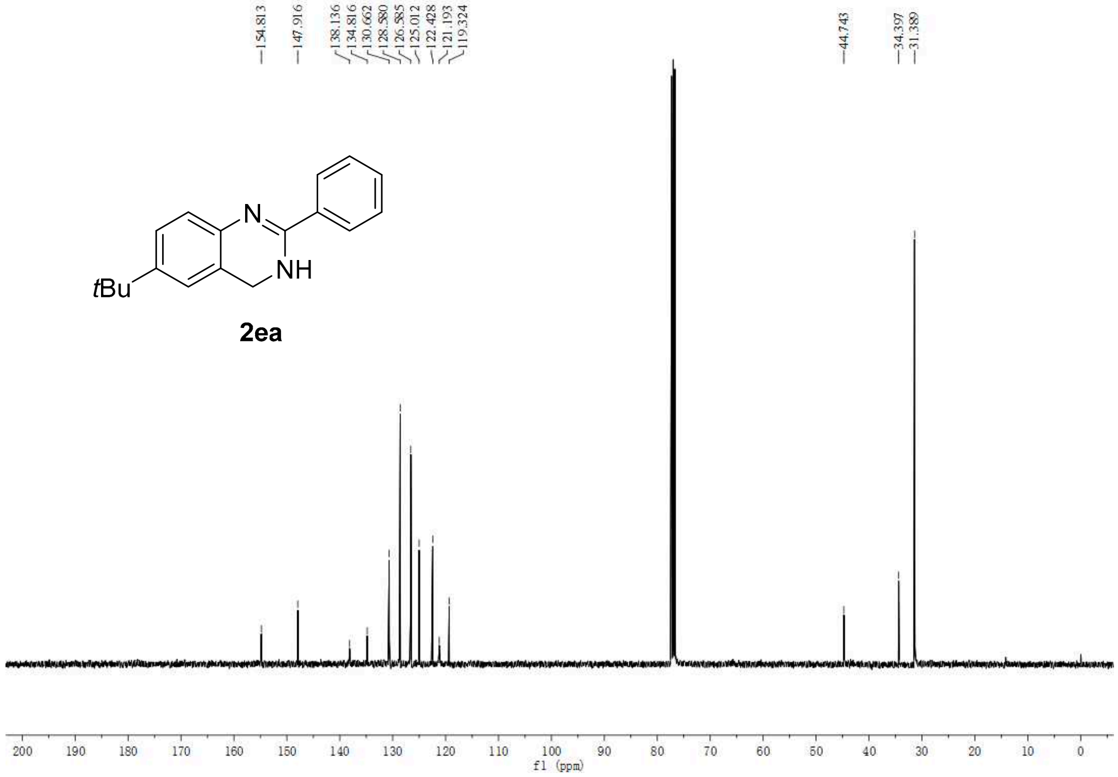

^{1}H NMR (400 MHz, $CDCl_3$) spectrum of **6a**

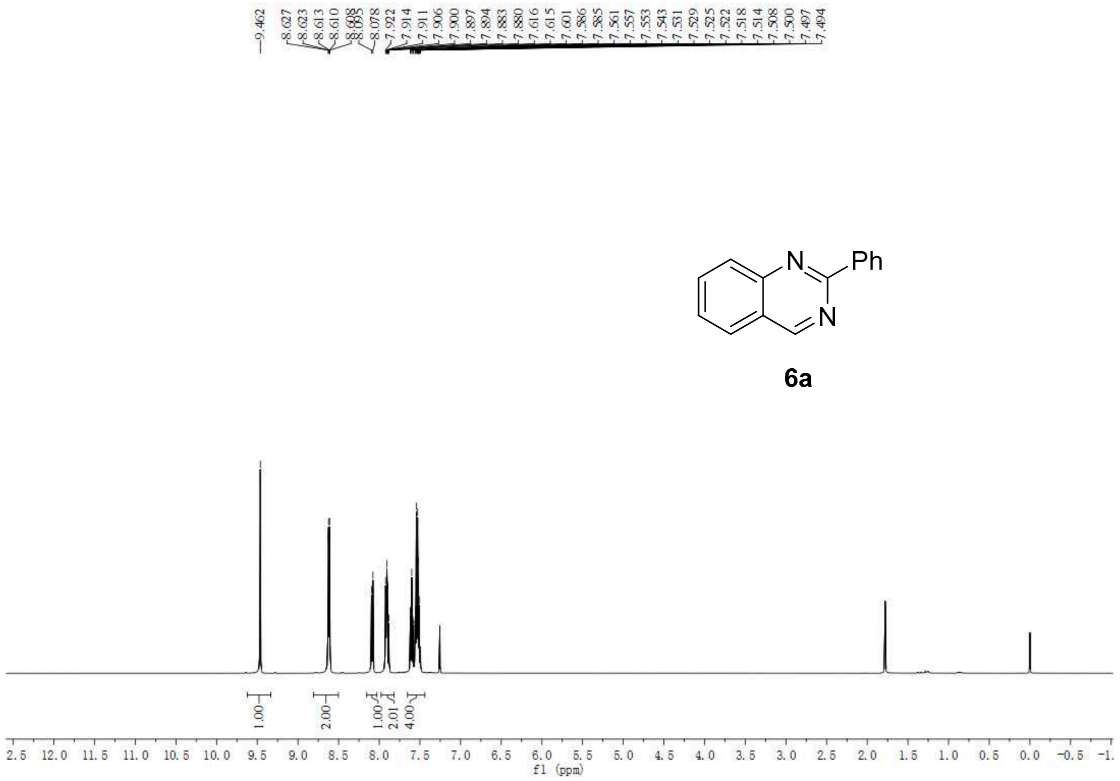

^{13}C {^{1}H} NMR (100 MHz, $CDCl_3$) spectrum of **6a**

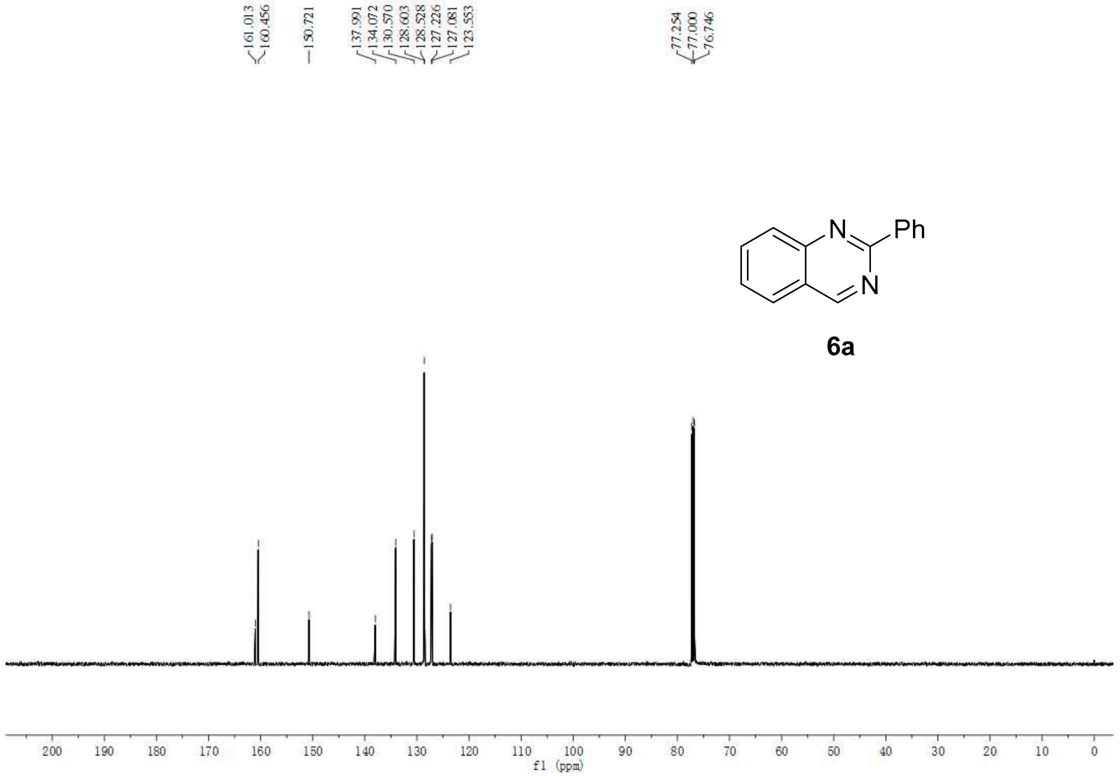

^{1}H NMR (400 MHz, $CDCl_3$) spectrum of **7a**

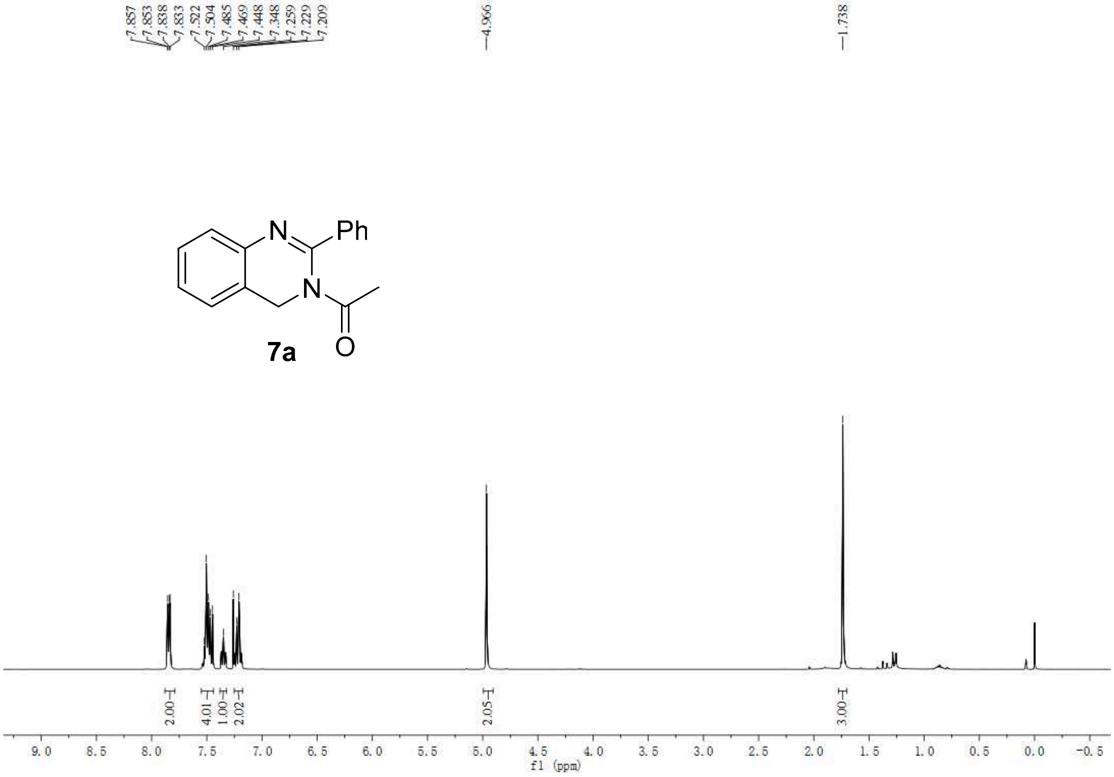

^{13}C {^{1}H} NMR (100 MHz, $CDCl_3$) spectrum of **7a**

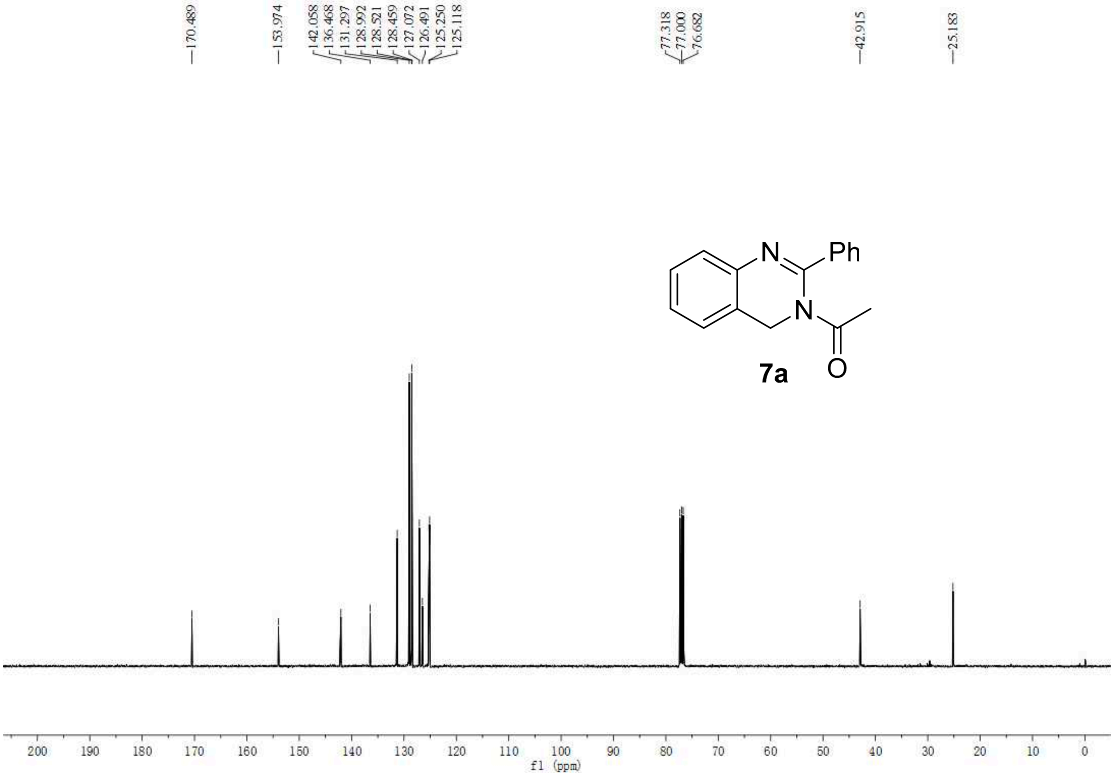

^{1}H NMR (400 MHz, $CDCl_3$) spectrum of **7b**

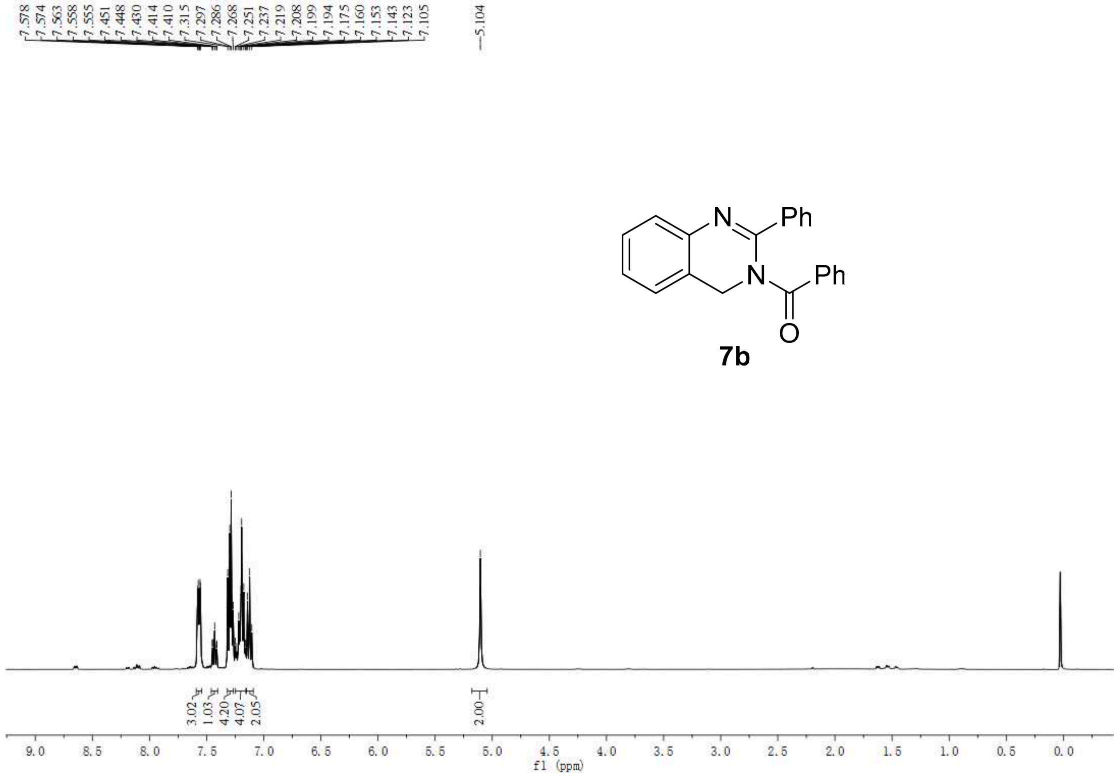

^{13}C {^{1}H} NMR (100 MHz, $CDCl_3$) spectrum of **7b**

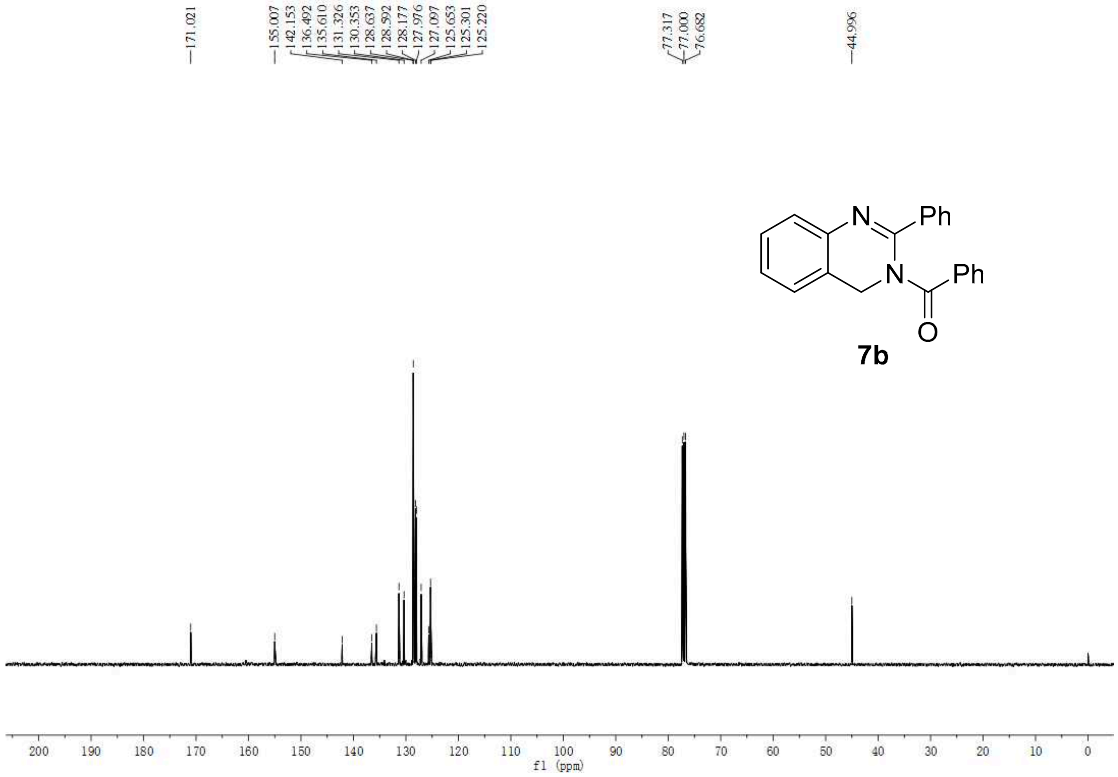

Supplement: Supplementary file 1 [file molecules-30-00350-s001.zip › molecules-3407738-supplementary.pdf]
